# Homocysteine and Multiple Health Outcomes: An Outcome-Wide Umbrella Review of Meta-analyses and Mendelian Randomization Studies

**DOI:** 10.1016/j.advnut.2025.100434

**Published:** 2025-04-25

**Authors:** Futao Zhou, Yue He, Xinhua Xie, Ning Guo, Wanjiao Chen, Yushi Zhao

**Affiliations:** 1 School of Basic Medicine, Gannan Medical University, Ganzhou City, Jiangxi Province, China; 2 Department of Dujiakan Outpatient, Jingnan Medical District of PLA General Hospital, Beijing, China

**Keywords:** umbrella review, meta-analysis, Mendelian randomization, interventional trial, colorectal cancer, stroke

## Abstract

Elevated levels of homocysteine (Hcy) are associated with various health outcomes. We aimed to systematically assess the credibility and certainty of evidence of associations of Hcy and Hcy-lowering therapies with various health outcomes. We retrieved observational meta-analyses examining the associations between Hcy and health outcomes, interventional meta-analyses investigating health outcomes related to Hcy-lowering treatments, and Mendelian randomization (MR) studies exploring the causal associations of Hcy with health outcomes to perform an umbrella review. A total of 135 observational meta-analyses, 106 MR studies, and 26 interventional meta-analyses were included. Among observational studies, 10 associations of diseases/outcomes were classified as highly suggestive; only 1 outcome (digestive tract cancer) was supported by convincing evidence (class I; odd ratio = 1.27, 95% confidence interval = 1.16, 1.40; *P* = 6.79 × 10^-7^; *I*^2^ = 0, 95% prediction interval excluding null, >1000 cases; *P* > 0.1 for tests of both small-study effects and excess significance bias). In MR studies, 5 outcomes associated with Hcy presented robust evidence (*P* < 0.01, power >80%). Among 25 outcomes explored by both observational meta-analyses and MR studies, 7 had consistent results, indicating that elevated Hcy is causally associated with an increased risk of these outcomes. The 3 types of studies collectively suggested that the association of stroke with Hcy was supported by observational studies, causally by MR studies, and further validated by intervention meta-analyses showing that Hcy-lowering with folic acid significantly reduced risk of stroke. For dementia and colorectal cancer, Hcy was significantly associated in meta-analyses of observational studies and folic acid decreased disease risks in interventional meta-analyses. The current umbrella review indicates that convincing evidence for a definitive role of Hcy exposure solely exists in the context of digestive tract cancer excluding bias; however, Hcy may not be causal for this disease. All the 3 types of studies collectively support that Hcy is a key causal risk factor, and Hcy-lowering (specifically with folic acid) may serve as an effective intervention for stroke.

This trial was registered at PROSPERO as CRD42024541335.

**Statement of significance**

Previous systematic reviews have not been summarized and appraised evidence of meta-analyses of observational and interventional studies, and Mendelian randomization (MR) studies on associations of homocysteine or homocysteine-lowering with a range of diseases (outcomes). Our umbrella review takes full advantage of the respective strengths of meta-analyses and MR studies by combining and comparing the findings to explore and assess the potential importance and implications of homocysteine for clinical practice and public health.

## Introduction

Homocysteine, a sulfur-containing amino acid derived from the methionine cycle, is metabolized via 2 key pathways: remethylation to methionine [dependent on folate/vitamin B_12_ and mediated by methylenetetrahydrofolate reductase (MTHFR)] or transsulfuration to cysteine (catalyzed by cystathionine β-synthase, CβS), with dysregulation in these pathways contributing to elevated plasma Hcy levels. Normal Hcy levels range from 5 to 15 μmol/L; hyperhomocysteinemia (HHcy) is defined as blood levels >15 μmol/L [1]. In 1969, Kilmer McCully first described the vascular pathology associated with homocystinuria [2]. Subsequently, numerous epidemiological reports have suggested that HHcy is an independent risk factor for various clinical conditions, including cardiovascular [3] and cerebrovascular [4] diseases, as well as dementia [5]. In China, the prevalence of HHcy was estimated to be 37.2% [6], with a gradual increase from 2013 to 2018, and higher among the elderly and men. Although clear associations exist between Hcy and cerebrovascular diseases, their causal relationships have not been firmly established [7]. Folate and vitamin B_12_ are important regulators in Hcy metabolism, and there exists an inverse relationship between folate and Hcy levels. Folic acid supplementation has been associated with a reduction of disease risk [[8], [9], [10]]. These observations suggest that folic acid supplementation holds promise as an effective measure for the prevention and treatment of these diseases [11].

The associations of Hcy with health outcomes explored in observational studies can be biased by confounding often from inaccurately measured, or unmeasured or even unknown sources. Therefore, the causal role of Hcy in these outcomes is widely questioned. Moreover, there exists a possibility that these associations may be representative of reverse causality. The credibility and certainty of associations between Hcy and disease outcomes remain to be determined. These inconclusive findings have resulted in a shift of interest away from Hcy, and asymptomatic HHcy has definitely not been considered an indication for Hcy-lowering treatment in patients. Furthermore, many interventional meta-analyses have focused on the effects of folic acid and other B vitamin supplementation or fortification on disease risk.

Given the potential importance of Hcy, assessing the credibility of the observed evidence may have profound implications for both clinical practice and public health. It is well recognized that different types of studies (observational, interventional and MR) have specific strengths and weaknesses that can complement each other. Although these evaluations are informative, quantitative assessments of bias are not perfect because they depend on reports from the original studies, and definite criteria are needed to determine the credibility of associations. An umbrella review, systematically collecting and evaluating evidence from multiple resources, might help clarify the complexity. To overcome these limitations, we carried out an outcome-wide umbrella review of observational and interventional [including both randomized controlled trials (RCTs) and non-RCTs] meta-analyses, and MR studies to summarize evidence regarding the effects of Hcy on multiple health outcomes. In particular, we have summarized the range of related health outcomes, the credibility, magnitude, direction, consistency, and significance of the associations and effects, assessed the potential biases, and identified which disease outcome(s) were causally affected by Hcy or HHcy, and confirmed whether Hcy was modifiable and the clinical implementation of Hcy-lowering was feasible for prevention or treatment of certain diseases.

## Methods

### Search strategy and study selection

Peer-reviewed relevant publications from 3 databases (PubMed, Embase, and Cochrane Database) were searched from inception to April 2024 using the following terms: ("meta-analysis" OR meta-analyses OR "Mendelian randomi∗") AND (homocysteine OR hyperhomocysteinemia) (details of search strategies are shown in Supplemental Table 1). Bibliographies of eligible studies and relevant meta-analyses were further hand-searched. Two researchers (FZ, YH) independently performed the literature search, study selection, and data extraction for this review. Discrepancies were resolved by a third investigator (WC). No language restrictions were imposed.

The health outcomes included a wide range of diseases and intermediate subtypes. Our inclusion criteria were as follows: *1*) systematic reviews with meta-analyses of observational studies examining associations between Hcy (or HHcy) and multiple health outcomes with a prospective cohort, cross-sectional or case-control design, with the meta-analytic summary estimates derived from ≥2 primary studies; *2*) meta-analyses of intervention or RCTs or quasi-RCTs that investigated health outcomes related to Hcy-lowering treatment (intervention with single or a combination of the B vitamins for lowering Hcy levels compared with placebo or no treatment); *3*) MR studies exploring the causal effects of Hcy on health outcomes using Hcy-related genetic instruments. Only formal quantitative meta-analyses or MR studies were considered.

**FIGURE 1:**
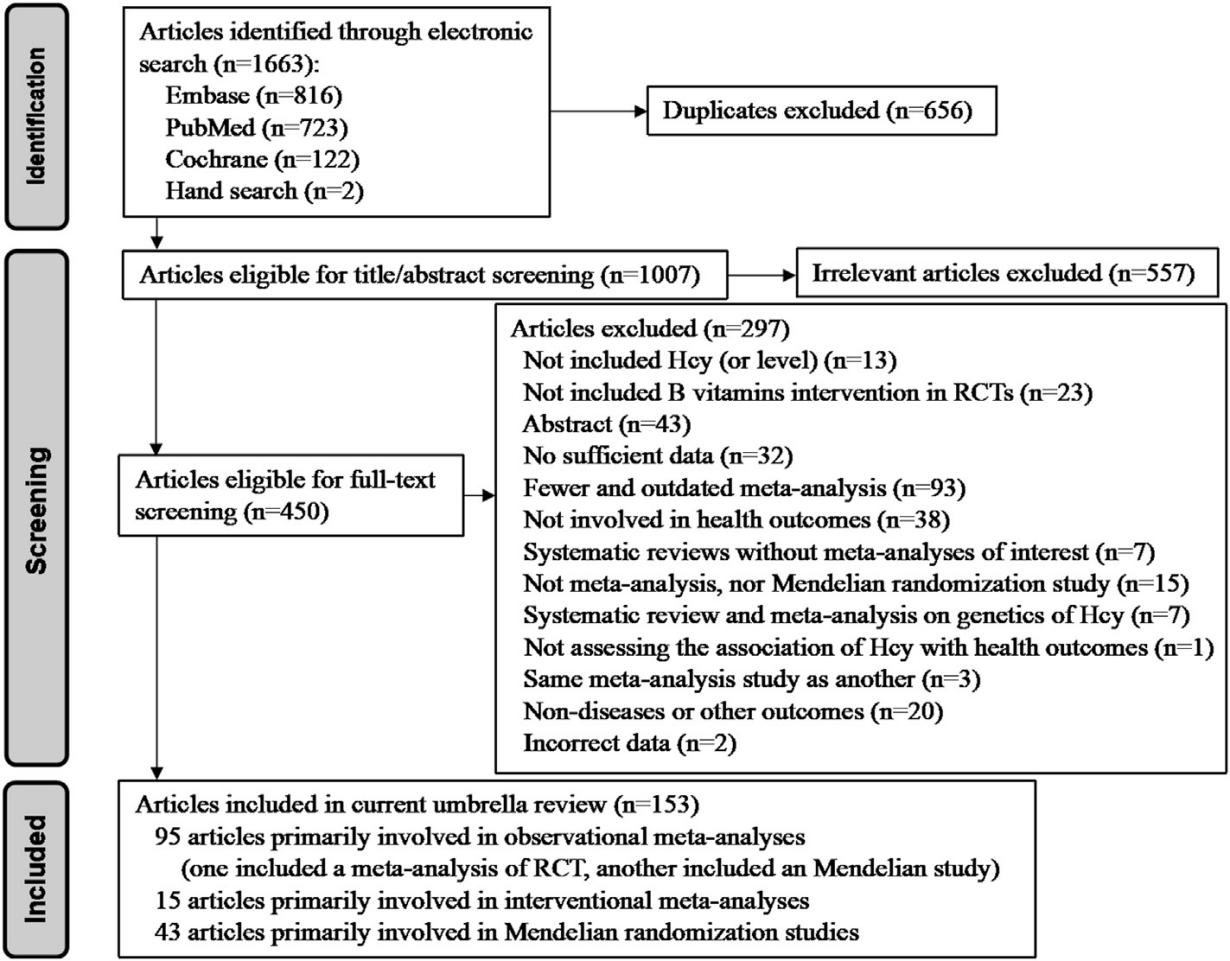
Screening and selection process of studies. RCT, randomized controlled trial.

Our primary exclusion criteria were as follows: *1*) systematic reviews without meta-analyses; *2*) when 2 or more meta-analyses presented overlapping data on the same association, only the one with the largest dataset was retained for the specific association; *3*) studies neither involving in health outcomes nor including Hcy (or its level); *4*) nondiseases, or other outcomes, such as lipid levels, carotid intima-media thickness, inflammatory markers, endothelial function, episodic memory, cognitive executive function or quality-of-life indicators, etc. Other exclusions were listed in Figure 1. We also excluded overlapping and outdated meta-analyses published earlier with fewer cohorts or datasets after comparison. For Hcy-lowering treatments with B vitamins, we included meta-analyses of RCTs investigating dietary or supplementary intake but excluded those analyzing blood (serum or plasma) levels of vitamins B.

### Data extraction

For each observational or interventional meta-analysis, we extracted first author's name (metareview’s author), year of publication, study population, number of studies included, health outcomes (diseases) investigated, number of cases and total participants (or controls), reported risk estimates [e.g. standardized mean difference, Hedges’ g, odds ratio (OR), or risk ratio] with the 95% confidence interval (CI), and primary study designs (e.g. cohort, case-control design) or Hcy-lowering agents for every initial studies. The quality of all eligible meta-analyses was assessed using the A Measurement Tool to Assess Systematic Reviews, 2nd edition (AMSTAR2) quality assessment tool [12].

For MR studies, we extracted data on the first author’s name, publication year, outcome population, number of participants and events, number of genetic instruments used, proportion variance of Hcy levels explained by the genetic instruments (*R*^2^), effect estimates (OR or regression coefficient *β*), level of exposure, and *P* value for effect size. The quality of all eligible MR studies was assessed based on the 3 core MR assumptions (relevance, independence, and exclusion).

### Data analysis

We utilized standardized methods for the umbrella review to evaluate findings on the associations of Hcy with multiple health outcomes [[13], [14], [15]]. Specifically, we re-estimated the summary effect size along with its 95% CI and *P* value for each meta-analysis using a random-effect model. We transformed each effect size into a comparable estimate [i.e. equivalent odds ratio (eOR)]. For between-study heterogeneity of effect sizes, we calculated the *I*^2^ statistic. Heterogeneity was assessed using *I*^2^ statistics. 95% prediction intervals (PIs) estimated expected effect size ranges for future studies [16]. Small-study effects were tested via Egger’s regression asymmetry test (significance threshold: *P* < 0.10) [17]. Excess significance bias was evaluated by comparing the observed number of significant studies (O) with the expected number (E), calculated by summing statistical power estimates of individual studies [18]. A chi-squared test identified excess significance (O > E).

For associations with class I–III evidence, we performed sensitivity analyses according to the study design of component studies. When there was >1 meta-analysis of observational studies investigating the identical outcome (disease), we restricted inclusion to prospective cohort or nested case-control studies (rather than case-control designs) to examine the temporality. This analysis assessed whether credibility levels changed.

For every MR study, we conducted a descriptive analysis. If all the necessary parameters for power calculations were provided (e.g. sample size, cases/controls, *R*^2^, effect estimates), we performed power calculations using the noncentrality parameter [19] via an online tool (https://sb452.shinyapps.io/power/). If *R*^2^ values were missing, we used conservative (1%) or approximate (3%) *R*^2^ estimates from MR studies using identical genetic instruments to crudely estimate statistical power. When multiple MR studies addressed the same outcome, we compared concordance in direction and significance of causal associations, and retained the study with the largest sample size or number of IVs [14].

### Assessment of evidence credibility

Following established umbrella review methodology [20], we categorized evidence strength from meta-analyses into 5 levels: convincing (class I), highly suggestive (class II), suggestive (class III), weak (class IV), and nonsignificant. Criteria included: *P* value for statistical significance, number of cases (or participants), *I*^2^, evidence of small-study effects and excess significance bias (*P* < 0.10), 95% PI excluding the null, and significance of the largest study. For example, convincing evidence required: >1000 cases (or >20,000 participants), summary effect *P* <10^–6^, 95% PI excluding the null, and *I*^2^ < 50%, the largest study *P* < 0.05, no small-study effects (*P* > 0.10), no excess significance bias (*P* > 0.10).

For MR studies, evidence robustness was categorized into 4 levels (robust, probable, suggestive, and insufficient evidence) [21]. Evidence was designated robust if the MR estimate had *P* < 0.01 and statistical power >80%.

### Assessment of consistency between observational meta-analyses and MR studies

To enhance interpretability, we compared effect size from observational meta-analyses and corresponding MR studies for the same disease. A ratio of odds ratios was calculated to quantify the MR-to-observational meta-analysis ratio. Log-OR differences and 95% CIs were derived under approximate normality assumptions, back-transformed to the raw scale [22,23]. A *z*-test assessed consistency; *P* < 0.05 indicated significant disagreement between study types [15,22].

A statistical association does not necessarily imply causality. When the associations of Hcy derived from observational meta-analyses and MR studies were strong and consistent, it could be inferred that Hcy was a causal factor for the outcome(s). Furthermore, we compared prior results of meta-analyses of observational studies and MR studies to assess the level of consistency with the data of meta-analyses of RCTs for the same disease (outcome). If meta-analyses of RCTs on Hcy had a high level of evidence, it could be inferred that Hcy was not only a causal but also a modifiable risk factor for the outcome. In contrast, if they were insignificant and inconsistent, Hcy was not believed to be a causal factor for the outcome due to confounding and reverse causality [15].

Analyses were performed in R software, version 4.1.2, available as an online version of the R statistical package called metaumbrella (https://metaumbrella.org/app) [24].

## Results

### Literature collection

A total of 1663 publications were identified across the 3 databases (PubMed, Embase, and Cochrane Database). After removing 656 duplicates and 557 irrelevant publications by reading the titles and abstracts of the articles, we further screened 450 publications by reading the full texts. Finally, 297 publications were excluded based on the exclusion criteria, leaving 153 articles that met the inclusion criteria (95 publications for observational meta-analyses, 15 for interventional meta-analyses, and 43 for MR studies). Of note, 1 publication [25] reported both an observational meta-analytic study and an MR study, whereas another [26] included both an observational meta-analytic study and an interventional meta-analysis. Additionally, a meta-analysis investigating the association of B vitamins (folate, vitamin B_6_, and vitamin B_12_) intake with risk of incident dementia (not Alzheimer’s disease) erroneously extracted Alzheimer’s disease data from Nelson et al.’s primary study [27]; we corrected this in our metareview. In total, the final analysis included 135 meta-analyses of observational studies (94 unique outcomes) from 95 articles, 26 interventional meta-analyses (20 outcomes) from 15 articles, and 106 MR studies from 43 articles (Figure 1).

### Meta-analyses of observational studies

A total of 135 unique meta-analyses were identified after the removal of overlapping meta-analyses (defined as those conducted in the same population, with the same outcome, and study design). These meta-analyses reported diverse health outcomes. The median values were 10 studies per meta-analysis (range: 2–128), 776 cases (range: 54–14,834), and 118 participants (range: 2532–86,177).

As shown in Supplemental Table 2, 117 meta-analyses (86.7%) reported statistically significant summary results (*P* < 0.05). There were 26 (19.3%) meta-analyses for cardiovascular disease (CVD) cohorts [3,[28], [29], [30], [31], [32], [33], [34], [35], [36], [37], [38], [39], [40], [41], [42], [43], [44], [45], [46], [47], [48]], 26 (19.3%) for neurocognitive disorder cohorts [5,25,[49], [50], [51], [52], [53], [54], [55], [56], [57], [58], [59], [60], [61], [62], [63], [64], [65], [66], [67], [68], [69]], 20 (14.8%) for obesity and metabolic disorders or cohorts [[70], [71], [72], [73], [74], [75], [76], [77], [78], [79], [80], [81], [82]], 13 (9.6%) for cancer and cause-specific mortality or cohorts [26,44,[83], [84], [85], [86], [87]], 6 (4.4%) for digestive orders or cohorts [[88], [89], [90], [91]], 7 (5.2%) for sense organ-related disorders [[92], [93], [94], [95], [96], [97]], 9 (6.7%) for reproductive and congenital disorders [[98], [99], [100], [101], [102], [103], [104], [105], [106]], and 16 (11.9%) for other outcomes [26,[107], [108], [109], [110], [111], [112], [113], [114], [115], [116]].

We then applied to the predefined evidence classification criteria. Forty-two (31.1%) meta-analyses had *P* <10^–6^, 15 (11.1%) had 95% PIs that excluded the null, 57 (42.2%) included >1000 cases, 46 (34.1%) exhibited low heterogeneity (*I*^2^ < 50%), and 67 (49.6%) had no evidence of small-study effects and excess significance bias. On the basis of these criteria, as shown in Supplemental Table 2, only 1 of 135 (0.7%) outcomes presented convincing evidence (class I: digestive tract cancer), 16 (11.9%) highly suggestive evidence [class II: first-time stroke, cerebral small-vessel disease, ischemic heart disease, coronary artery disease, peripheral arterial disease, Alzheimer’s disease, Parkinson’s disease, schizophrenia, type 2 diabetes (cross-sectional), Behçet’s syndrome, all-cause mortality in general population, all-cause mortality in patients with acute ischemic stroke, ulcerative colitis, polycystic ovary syndrome, rheumatoid arthritis, chronic kidney disease], 23 (17%) suggestive evidence, and 77 (57%) weak evidence (class IV). The remaining 18 (13.3) had insignificant evidence.

**FIGURE 2:**
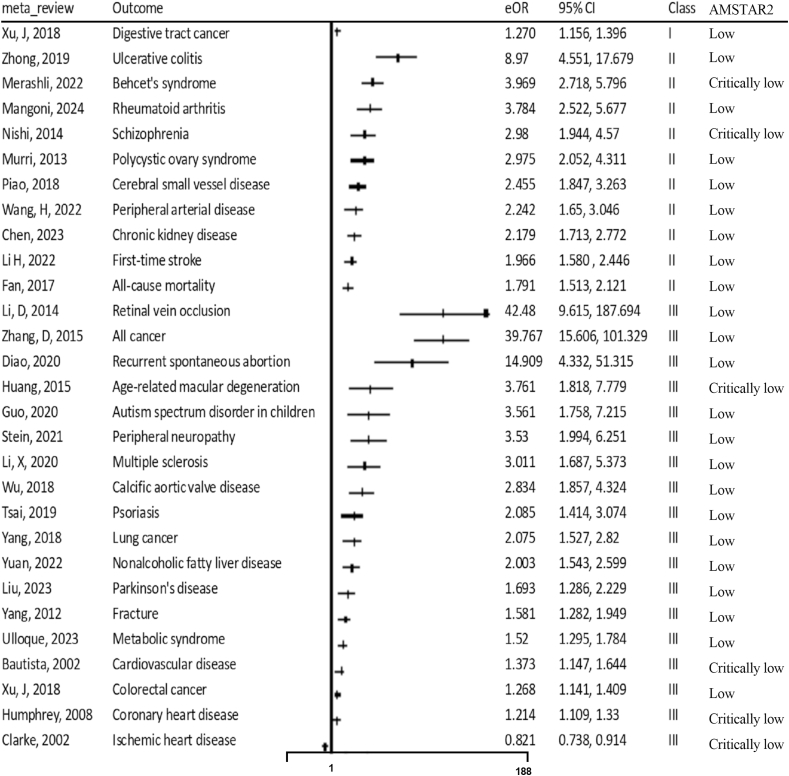
Forest plot of summary estimates from observational studies reporting associations of Hcy with multiple health outcomes, stratified by evidence classes I–III. Class I: >1000 cases or >20,000 participants, summary effect *P* < 10^–6^, 95% PI excluding the null, and *I*^2^ < 50%, the largest study *P* < 0.05, no small-study effects (*P* > 0.10), no excess significance bias (*P* > 0.10). Class II: >1000 cases or >20,000 participants, summary effect *P* < 10^–6^, the largest study *P* < 0.05. Class III: >1000 cases or >20,000 participants, summary effect *P* < 10^–3^, the largest study *P* > 0.05. AMSTAR2, A Measurement Tool to Assess Systematic Reviews, 2nd edition; CI, confidence interval; eOR, equivalent odds ratio; Hcy, homocysteine; PI, prediction interval.

**FIGURE 3:**
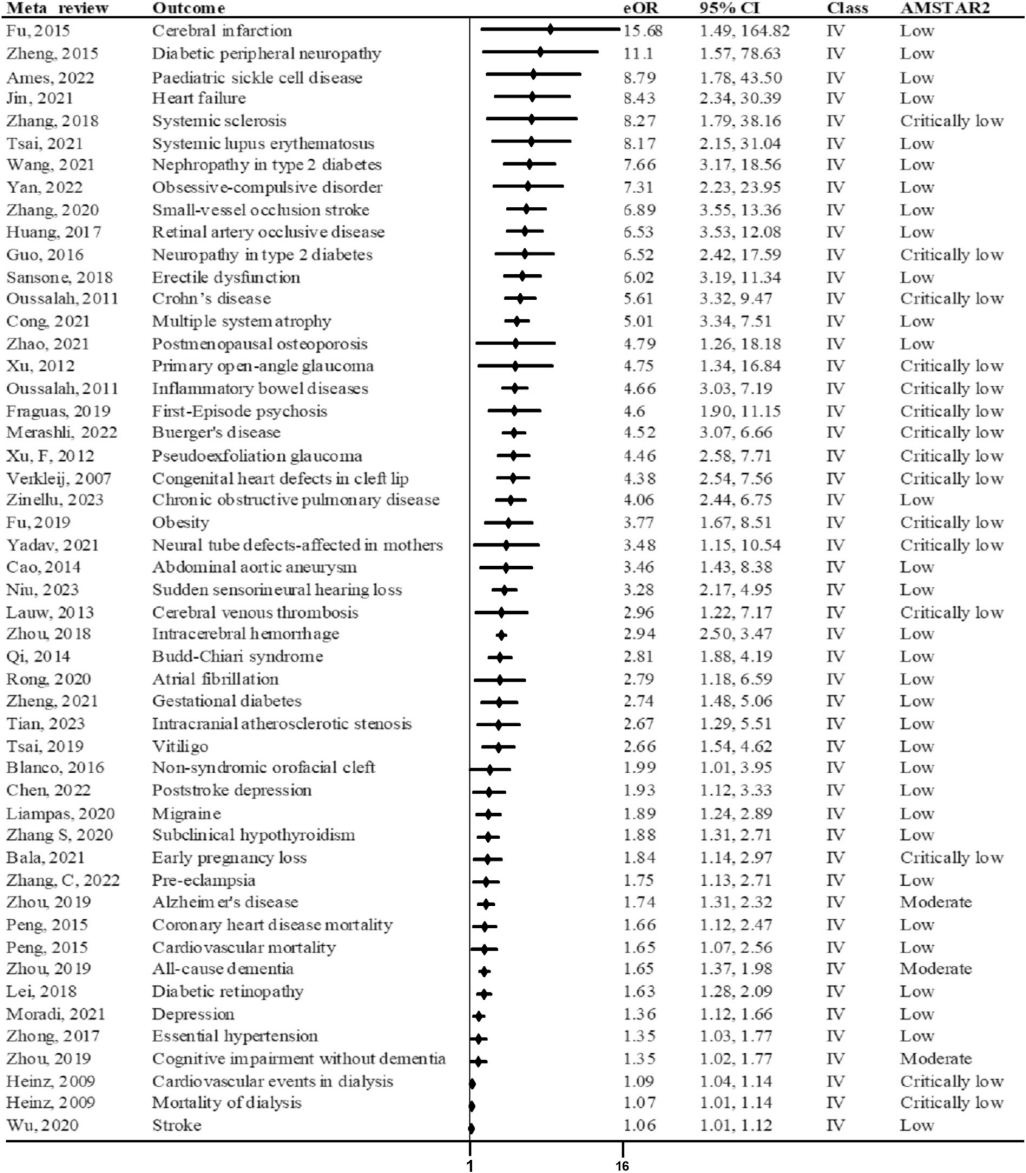
Forest plot of summary estimates from observational studies reporting associations of Hcy with multiple health outcomes, stratified by evidence classes I–III. Class I: >1000 cases or >20,000 participants, summary effect *P* < 10^–6^, 95% PI excluding the null, and *I*^2^ < 50%, the largest study *P* < 0.05, no small-study effects (*P* > 0.10), no excess significance bias (*P* > 0.10). Class II: >1000 cases or >20,000 participants, summary effect *P* < 10^–6^, the largest study *P* < 0.05. Class III: >1000 cases or >20,000 participants, summary effect *P* < 10^–3^, the largest study *P* > 0.05. AMSTAR2, A Measurement Tool to Assess Systematic Reviews, 2nd edition; CI, confidence interval; eOR, equivalent odds ratio; Hcy, homocysteine; PI, prediction interval.

For the same diseases investigated by meta-analyses based on different study designs (prospective cohort, case-control, or cross-sectional), we performed sensitivity analyses and confined the meta-analyses to prospective cohort studies. The evidence was downgraded as follows: stroke from class II to IV, ischemic stroke from class III to IV, ischemic heart disease from class II to III, and coronary artery disease from class II to III. Additionally, when extending a single-sex population (male or female) to a mixed-sex population, the evidence for schizophrenia (class IV) was upgraded to class II. Furthermore, we removed these associations for the same diseases/outcomes and ultimately included 93 diseases/outcomes (Supplemental Table 2). Overall, only outcome (digestive tract cancer) out of 93 (1.1%) presented convincing evidence, 10 (10.8%) were highly suggestive (class II: ulcerative colitis, Behçet’s syndrome, rheumatoid arthritis, schizophrenia, polycystic ovary syndrome, cerebral small-vessel disease, peripheral arterial disease, chronic kidney disease, first-time stroke, all-cause mortality), 18 (19.4%) were suggestive (class III), and 50 (53.8%) were weak (class IV), as shown in FIGURE 2, FIGURE 3 and Supplemental Table 3.

For the meta-analytic association of digestive tract cancer (convincing evidence) reported only by an article [87], we performed a thorough examination of the original studies included in the meta-analysis, and confirmed that the data (highest compared with lowest categories, number of cases and controls) were accurate in each individual study. The authors performed an overall analysis on digestive tract cancer and several subgroup analyses, including gastric cancer and colorectal cancer (CRC) subgroups. Our umbrella review specifically analyzed CRC data and found that its association had the same effect size as digestive tract cancer, but the former was classified as class III evidence (Figure 2).

### MR studies

A total of 106 primary MR analyses were identified from 46 publications covering 81 distinct outcomes and phenotypes (Supplemental Table 4). Two-sample MR analyses were performed in the majority of MR studies. The most commonly used single-nucleotide polymorphisms serving as IVs were MTHFR-C677T (*n* = 15; 14.2%). These MR studies explored the following outcomes: cardiovascular outcomes [[117], [118], [119], [120], [121], [122], [123], [124], [125], [126], [127], [128], [129], [130]] (*n* = 29), neurocognitive disorders [25,125,[131], [132], [133], [134], [135], [136], [137], [138]] (*n* = 14), obesity and metabolic disorders [[139], [140], [141], [142]] (*n* = 3), digestive disorders [90,143,144] (*n* = 3), cancer and cause-specific mortality [[145], [146], [147], [148], [149]] (*n* = 8), bone and joint disorders [[150], [151], [152], [153]] (*n* = 16), and others [[154], [155], [156], [157], [158]] (*n* = 8). The median number of participants was 7158 (range 828–1,146,185), and the number of cases was 2225 (99–139,364) in outcome samples. The proportion of exposure variance (*R*^2^) explained by IVs ranged from 1% to 6%. Multiple MR studies investigated the same diseases/outcomes as follows: ischemic stroke (*n* = 3), intracranial aneurysm (*n* = 2), Alzheimer’s disease (*n* = 6), multiple sclerosis (*n* = 2), nonalcoholic fatty liver disease (*n* = 2), and gastric cancer (*n* = 2). On the basis of our selection criteria, we further excluded some MR studies, and ultimately identified 81 unique disease-association pairs (Table 1) [109,[117], [118], [119],^121^,[124], [125], [126], [127], [128], [129],^133^,^134^,[136], [137], [138],[140], [141], [142], [143], [144], [145],[147], [148], [149], [150], [151], [152], [153], [154],[156], [157], [158]]. Among 81 pairs, 74 (91%) demonstrated good reporting quality adherence to MR core assumptions.

**TABLE 1:** Characteristics and statistical power of the eligible MR studies investigating putative homocysteine-outcome relationships.

Author/year	Outcome or phenotype	Outcome popul.	No./no. of events	No. IVs	*R*^2^ (%)	Metric	Estimate of effect (95% CI)	*P* value	Power	Level of exposure	Core summp (Rel/Ind/Ex)
Cardiovascular outcomes											
Yuan et al., 2021 [118]	Coronary artery disease	Mixed	724,160/139,364	27	6	OR	1.05 (0.96, 1.15)	0.264	0.98	per 1 SD inc	Y/Y/Y
Miao et al., 2021 [119]	Acute myocardial infarction	Euro	NR/181,875	9	NR	OR	1.04 (0.93, 1.14)	0.499	NA	per 1-unit inc	Y/Y/Y
Xu et al., 2021 [121]	Coronary artery disease in DM	Euro	15,666/3968	9	NR	OR	1.14 (0.82, 1.58)	0.43	NA	NR	Y/Y/Y
Yuan et al., 2021 [118]	Heart failure	Euro	1,146,185/56,885	27	6	OR	0.96 (0.88, 1.05)	0.372	0.642	per 1 SD inc	Y/Y/Y
Yuan et al., 2021 [118]	Atrial fibrillation	Euro	1,145,375/77,945	27	6	OR	0.96 (0.91, 1.01)	0.098	0.769	per 1 SD inc	Y/Y/Y
Wang et al., 2023 [124]	Congestive heart failure	Euro	456,348/897	3	NR	OR	1.75 (0.67, 4.56)	0.25	NA	per 1 SD inc	Y/Y/Y
Wang et al., 2023 [124]	Cardiomyopathy	Euro	159,811/3100	12	NR	OR	0.81 (0.58, 1.11)	0.189	NA	per 1 SD inc	Y/Y/Y
Wang et al., 2023 [124]	Non-ischemic cardiomyopathy	Euro	1,763,152/11,400	12	NR	OR	1.06 (0.93, 1.22)	0.379	NA	per 1 SD inc	Y/Y/Y
Yuan et al., 2021 [118]	Aortic valve stenosis	Euro	367,561/3528	14	6	OR	1.14 (0.86, 1.5)	0.356	0.475	per 1 SD inc	Y/Y/Y
Yuan et al., 2021 [118]	Aortic aneurysm	Euro	5,373,323/4180	27	6	OR	1.11 (0.92, 1.35)	0.286	0.379	per 1 SD inc	Y/Y/Y
Yuan et al., 2021 [118]	Stroke	Mixed	961,455/66792	41	6	OR	1.11 (1.03, 1.20)	0.008	1	per 1 SD inc	Y/Y/Y
Yuan et al., 2021 [118]	Subarachnoid hemorrhage	Euro	243,956 /8514	26	6	OR	1.26 (1.05, 1.51)	0.013	0.999	per 1 SD inc	Y/Y/Y
Liu et al., 2021 [125]	Ischemic stroke	Euro	440,328/17,265	13	5.9	OR	1.10 (0.98, 1.23)	0.107	0.833	NR	Y/Y/Y
Yuan et al., 2021 [118]	Intracerebral hemorrhage	Euro	539,266/5951	39	6	OR	1.09 (0.89, 1.34)	0.411	0.367	per 1 SD inc	Y/Y/Y
Ma et al., 2022 [126]	Aneurysmal subarachnoid hemorrhage	Euro	77,074/5140	9	NR	OR	1.10 (0.88, 1.39)	0.398	NA	NR	Y/Y/Y
Liu et al., 2021 [125]	Large artery atherosclerosis stroke	Euro	440,328/4373	13	5.9	OR	1.09 (0.88, 1.31)	0.424	0.295	NR	Y/Y/Y
Liu et al., 2021 [125]	Cardioembolism stroke	Euro	440,328/7193	13	5.9	OR	0.92 (0.79, 1.08)	0.308	0.399	NR	Y/Y/Y
Liu et al., 2021 [125]	Small artery occlusion stroke	Euro	440,328/5386	13	5.9	OR	1.33 (1.00, 1.76)	0.048	0.999	NR	Y/Y/Y
Larsson et al., 2019 [117]	Small-vessel stroke	Euro	410,016/5386	18	5.9	OR	1.34 (1.13, 1.58)	6.7E-04	0.998	per 1 SD inc	Y/Y/Y
Larsson et al., 2019 [117]	Large artery stroke	Euro	409,003/4373	18	5.9	OR	1.01 (0.84, 1.21)	0.89	0.035	per 1 SD inc	Y/Y/Y
Larsson et al., 2019 [117]	Cardioembolic stroke	Euro	411,823/7193	18	5.9	OR	0.94 (0.81, 1.07)	0.35	0.056	per 1 SD inc	Y/Y/Y
Cao et al., 2021 [127]	Lacunes	Chinese	1023/139	1	1	OR	2.14 (1.4, 3.27)	<0.00001	0.13	NR	Y/Y/Y
Wen et al., 2023 [128]	Intracranial aneurysm	Euro	79,429/7495	9	NR	OR	1.38 (1.07, 1.79)	0.018	NA	per 1 SD inc	Y/Y/Y
Yuan et al., 2021 [118]	Transient ischemic attack	Euro	538,576/11,542	28	6	OR	1.15 (0.99, 1.33)	0.066	0.953	per 1 SD inc	Y/Y/Y
Ma et al., 2022 [126]	Unruptured intracranial aneurysm	Euro	74,004/2070	7	NR	OR	1.13 (0.68, 1.86)	0.644	NA	NR	Y/Y/Y
Yuan et al., 2021 [118]	Venous thromboembolism	Euro	544,460/23,325	27	6	OR	1.05 (0.94, 1.16)	0.392	0.431	per 1 SD inc	Y/Y/Y
Yuan et al., 2021 [118]	Peripheral arterial disease	Euro	540,727/9916	27	6	OR	1.06 (0.91, 1.23)	0.486	0.291	per 1 SD inc	Y/Y/Y
Fu et al., 2019 [129]	Hypertension	Mixed	40,173/14,378	1	1	OR	1.32 (1.22, 1.49)	NR	0.76	per 5-unit inc	Y/N/N
Li et al., 2019 [130]	Hypertension in pregnancy	Chinese	2188/1077	1	1	OR	3.21 (2.36, 4.07)	7.4E-04	0.779	per 1 SD inc	Y/N/N
Neurocognitive disorders											
Liu et al., 2021 [125]	Alzheimer' disease	Euro	63,926/21,982	13	5.9	OR	1.08 (0.96, 1.22)	0.198	0.623	NR	Y/Y/Y
Liu et al., 2021 [125]	Frontotemporal dementia	Euro	3024/515	13	5.9	OR	1.27 (0.42, 3.86)	0.676	0.221	NR	Y/Y/Y
Wu et al., 2017 [133]	Vascular dementia	Mixed	1880/722	1	1	OR	4.29 (1.11, 16.57)	0.034	0.867	per 1 SD inc	Y/N/N
Zhao et al., 2021 [109]	Parkinson's disease	Euro	482,730/33,674	14	NR	OR	1.01 (0.88, 1.16)	0.868	NA	per 1 SD inc	Y/Y/Y
Zhao et al., 2021 [109]	Age at onset in PD	Euro	467,052/17,996	14	NR	beta	–0.65 (–1.7, 0.4)	0.222	NA	per 1 SD inc	Y/Y/Y
Liu et al., 2021 [125]	Amyotrophic lateral sclerosis	Euro	80,610/20,806	13	5.9	OR	1.09 (0.95, 1.24)	0.235	0.692	NR	Y/Y/Y
Peng et al., 2021 [134]	Multiple sclerosis	Euro	115,803/47,429	14	6	OR	0.78 (0.64, 0.94)	0.0106	1	per 1 SD inc	Y/Y/Y
Yu J et al., 2022 [136]	Schizophrenia	Euro	161,405/67,390	10	22	OR	1.11 (1.03, 1.20)	2.7E-03	0.842	NR	Y/Y/Y
Yu J et al., 2022 [136]	Bipolar disorder	Euro	413,466/41,917	11	NR	OR	1.08 (1.00, 1.17)	0.054	NA	NR	Y/Y/Y
Yu J et al., 2022 [136]	BD-I type	Euro	475,038/25,060	13	3^2^	OR	1.13 (1.03, 1.25)	9.4E-03	0.915	NR	Y/Y/Y
Yu J et al., 2022 [136]	BD-II type	Euro	370,856/6781	13	NR	OR	0.98 (0.83, 1.15)	0.773	NA	NR	Y/Y/Y
Jin et al., 2024 [137]	Autism spectral disorder	Euro	46,351/18,382	13	NR	OR	1.03 (0.92, 1.15)	0.63	NA	NR	Y/Y/Y
Yu J et al., 2022 [136]	Major depressive disorder	Euro	42,455/16,823	13	NR	OR	0.95 (0.89, 1.01)	0.115	NA	NR	Y/Y/Y
Gao et al., 2024 [138]	Brain atrophy	Caucasian British	7916/NR	9	NR	OR	0.96 (0.81, 1.14)	NR	NA	NR	Y/Y/Y
Obesity and metabolic disorders											
Cheng et al., 2022 [140]	T2DM	Euro	898,130/74,124	14	6	OR	1.08 (0.95, 1.21)	0.249	0.998	per 1 SD inc	Y/Y/Y
Ma et al., 2019 [141]	Diabetic kidney disease	Chinese	1107/547	1	1	OR	3.86 (1.21, 2.05)	<0.001	0.613	per 5-unit inc	Y/N/N
Lee et al., 2021 [142]	Metabolic syndrome	Korea	5902/2090	5	NR	beta	0.723 (0.50, 0.94)	<0.001	0.87	per 1 SD inc	Y/Y/Y
Digestive disorders											
Fu et al., 2023 [143]	NAFLD	Euro	797,878/9917	9	6	OR	1.25 (1.05, 1.45)	0.008	1	per 1 SD inc	Y/Y/Y
Chen et al., 2022 [144]	Nonalcoholic steatohepatitis	Euro	30,9154/99	12	4^2^	OR	1.89 (0.51, 7.02)	0.341	0.244	NR	Y/Y/Y
Chen et al., 2022 [144]	NAFLD-related cirrhosis	Euro	306,971/826	12	4^2^	OR	0.81 (0.50, 1.32)	0.401	0.224	NR	Y/Y/Y
Cancer and cause-specific mortality											
Wang et al., 2020 [145]	Gastric cancer	Chinese Han	7004/2631	15	6	OR	1.07 (1.01, 1.12)	0.011	0.099	per 1-unit inc	Y/Y/Y
He et al., 2021 [147]	Breast cancer	Euro	267,173/133,384	15	NR	OR	0.97 (0.90, 1.06)	0.543	NA	NR	Y/Y/Y
He et al., 2021 [147]	Prostate cancer	Euro	140,254/79,148	15	NR	OR	1.01 (0.93, 1.11)	0.774	NA	NR	Y/Y/Y
He et al., 2021 [147]	Renal cell carcinoma in men	Euro	8143/3227	15	NR	OR	0.99 (0.73, 1.34)	0.929	NA	NR	Y/Y/Y
He et al., 2021 [147]	Renal cell carcinoma in women	Euro	5087/1992	15	NR	OR	0.89 (0.61, 1.31)	0.563	NA	NR	Y/Y/Y
Xuan et al., 2016 [148]	Multiple myeloma	Mixed	7046/2092	1	1	OR	2.67 (1.12, 6.38)	0.027	0.965	per 1 SD inc	Y/N/N
Choi et al., 2023 [149]	All-cause mortality	Mixed	10,005/1691	1	3.9	RR	0.99 (0.62, 1.57)	NR	0.03	per 2-fold inc	Y/N/N
Choi et al., 2023 [149]	CVD mortality	Mixed	10,005/240	1	3.9	RR	1.76 (0.54, 5.77)	NR	0.401	per 2-fold inc	Y/N/N
Bone and Joint disorders											
Wang et al., 2021 [150]	Bone fracture	Mixed	NR/426,795	5	1.78	OR	0.97 (0.88, 1.07)	0.562	NA	per 1-unit inc	Y/Y/Y
Hong et al., 2023 [151]	Overall osteoarthritis	>99% Euro	826,690/17,7517	11	1^1^	OR	1.10 (1.04, 1.16)	0.001	0.937	NR	Y/Y/Y
Hong et al., 2023 [151]	Hip osteoarthritis	Euro	353,388/36,445	11	1^1^	OR	1.17 (1.03, 1.33)	0.015	0.814	NR	Y/Y/Y
Hong et al., 2023 [151]	Spine osteoarthritis	>98% Euro	333,950/28,372	11	3^2^	OR	1.11 (1.02, 1.22)	0.02	0.842	NR	Y/Y/Y
Hong et al., 2023 [151]	Hand osteoarthritis	Euro	303,782/20,901	11	NR	OR	1.04 (0.87, 1.24)	0.657	NA	NR	Y/Y/Y
Hong et al., 2023 [151]	Thumb osteoarthritis	Euro	247,455/10,536	11	NR	OR	1.06 (0.86, 1.29)	0.592	NA	NR	Y/Y/Y
Fu et al., 2022 [152]	Knee osteoarthritis	Euro	455,221/76,932	14	6	OR	1.12 (1.03, 1.21)	0.007	1	per 1 SD inc	Y/Y/Y
Fu et al., 2022 [152]	Hospital-diagnosed osteoarthritis	Euro	327,918/30,824	14	6	OR	1.18 (1.01, 1.37)	0.034	1	per 1 SD inc	Y/Y/Y
Fu et al., 2022 [152]	Osteoporosis with pathological fracture	Euro	173,619/868	13	6	OR	1.60 (1.04, 2.46)	0.034	0.921	per 1 SD inc	Y/Y/Y
Fu et al., 2022 [152]	Soft tissue disorder	Euro	218,792/115,741	13	6	OR	1.07 (1.00, 1.14)	0.045	0.968	per 1 SD inc	Y/Y/Y
Wang et al., 2021 [150]	Forearm bone mineral density	Mixed	10,805/NR	8	1.78	beta	–0.111 (0.076)	0.153	NA	per 1-unit inc	Y/Y/Y
Wang et al., 2021 [150]	Femoral neck bone mineral density	Mixed	49,988/NR	5	1.78	beta	–0.02 (0.058)	0.731	NA	per 1-unit inc	Y/Y/Y
Wang et al., 2021 [150]	Lumbar spine bone mineral density	Mixed	44,731/NR	5	1.78	beta	–0.001 (0.068)	0.989	NA	per 1-unit inc	Y/Y/Y
Wang et al., 2021 [150]	Estimated heel bone mineral density	Mixed	426,824/NR	5	1.78	beta	0.028 (0.0398)	0.468	NA	per 1-unit inc	Y/Y/Y
Wang et al., 2023 [153]	Forearm bone mineral density	Euro	8143/NR	8	NR	OR	0.96 (0.77, 1.19)	0.69	NA	per 1 SD inc	Y/Y/Y
Wang et al., 2023 [153]	Lumbar bone mineral density	Euro	28,498/NR	7	NR	OR	0.86 (0.72, 1.02)	0.077	NA	per 1 SD inc	Y/Y/Y
Wang et al., 2023 [153]	Heel bone mineral density	Euro	142,487/NR	8	NR	OR	0.96 (0.93, 0.99)	0.011	NA	per 1 SD inc	Y/Y/Y
**Others**											
Hu et al., 2023 [154]	COPD-related chronic infections	Euro	186,957/234	14	NR	OR	1.50 (0.57, 3.99)	0.41	NA	NR	Y/Y/Y
Hu et al., 2023 [154]	COPD/asthma/ILD-related pneumonia or pneumonia-derived septicemia	Euro	187,582/27,715	14	NR	OR	0.93 (0.86, 1.02)	0.13	NA	NR	Y/Y/Y
Hu et al., 2023 [154]	COPD-related respiratory insufficiency	Euro	187,754/1031	14	NR	OR	1.00 (0.70, 1.44)	0.99	NA	NR	Y/Y/Y
Hu et al., 2023 [154]	COPD hospital admissions	Euro	218,792/6500	14	NR	OR	1.06 (0.91, 1.24)	0.42	NA	NR	Y/Y/Y
Hu et al., 2023 [154]	Asthma/COPD	Euro	208,167/21,444	14	NR	OR	0.97 (0.89, 1.06)	0.55	NA	NR	Y/Y/Y
Xiong et al., 2022 [156]	Chronic kidney disease	Mixed	530,537/27,900	NR	NR	OR	1.24 (1.07, 1.44)	<0.05	NA	per 1 SD inc	Y/Y/Y
Kjaergaard et al., 2022 [157]	Pregnancy loss	Euro	194,174	18	5.9	beta	−0.00 (−0.04, 0.03)	NR	NA	per 1 SD inc	Y/Y/Y
Chen et al., 2023 [158]	Psoriasis	Euro	373,338/9267	11	NR	OR	1.00 (0.86, 1.15)	0.941	NA	NR	Y/Y/Y

Abbreviations: BD, bipolar disorder; CI, confidence interval; COPD, chronic obstructive pulmonary disease; CVD, cardiovascular disease; DM, diabetes mellitus; Euro, European; ILD, interstitial lung disease; inc, increase; IV, instrumental variable; MR, Mendelian Randomization; NA, not applicable; NAFLD, Nonalcoholic fatty liver disease; NR, not report; OR, odds ratio; PD, Parkinson's disease; T2DM, type 2 diabetes mellitus.

Statistical power was not calculated (NA) if MR studies lacked required data (e.g. *R*^2^, sample size, cases). Population labels (e.g. “Euro,” “Caucasian”) retain original authors’ terms, and “Euro” = European ancestry populations with genetic confirmation, "Caucasian" = Used exclusively when explicitly defined in source publications, "Mixed" = undifferentiated cohorts with ≥3 ethnicities represented. For study quality assessment, record whether each of 3 core assumptions—Relevance (Rel), Independence (Ind), and Exclusion (Ex)—was addressed, and note the response as yes (Y) or no (N).

1 Indicates a more conservative value.

2 Indicates an approximate value.

In contrast to the results of observational meta-analyses, which demonstrated significant associations with most outcomes (84.8%), the majority of the MR studies (87.7%) were neither statistically significant nor had high statistical power. Of the 81 outcomes in MR studies, 25 presented both statistical significance (*P* < 0.05) and statistical power >80%. Notably, 12 outcomes (stroke, small-vessel stroke, lacunes, hypertension, hypertension in pregnancy, schizophrenia, bipolar disorder I type, diabetic nephropathy, metabolic syndrome, nonalcoholic fatty liver disease, overall osteoarthritis, and knee osteoarthritis) had *P* values <0.01. Of these 12 outcomes, 7 (stroke, small-vessel stroke, schizophrenia, bipolar disorder I type, metabolic syndrome, nonalcoholic fatty liver disease, overall osteoarthritis, and knee osteoarthritis) were characterized by statistical powers of 80% or more, indicating that strong evidence for the causal effects of Hcy on the 7 outcomes.

### Interventional meta-analyses

We identified 26 meta-analyses of intervention studies (or RCTs) on Hcy-lowering treatment with B vitamin complex, or a single or various combinations of vitamin B components from 16 publications. The eligible meta-analyses of RCTs were published between 2009 and 2022. The median number of studies included in the meta-analyses was 5 (range: 2–25) and of participants was 10,539 (710–6,165,894) as shown in Table 2 [9,10,26,[159], [160], [161], [162], [163], [164],[166], [167], [168], [169], [170], [171]].

**TABLE 2:** Characteristics and quantitative synthesis of meta-analyses on homocysteine-lowering interventions across diverse health outcomes.

Metareview	Outcome	Population	Hcy-lowering treatment	Study (*N*)	Participants (*N*)	Metric	eOR (95% CI)	*P* value	*I*^2^	*P* Egg	*P* for ESB	95% PI	LSS	Level	AMSTAR2
Li et al., 2016 [159]	Stroke	Patients with CKD, CVD or stroke, CAD and MI, and so on	Folic acid	20	77,816	RR	0.89 (0.81, 0.97)	0.0122	30.4	0.55	0.35	0.71, 1.10	0.69, 0.92	IV	Low
Zhang et al., 2013 [160]	Stroke	Patients with CKD, CVD or stroke, colorectal adenomas and no previous invasive large intestine carcinoma, esophageal dysplasia or healthy individuals	Folic acid/vitamin B_12_/B_6_	18	54,153	RR	0.92 (0.84, 1.01)	0.0632	24.6	0.49	0.28	0.79, 1.08	0.86, 1.20	NS	Low
Park et al., 2016 [10]	Stroke	Individuals not taking antiplatelet agents	B vitamins	3	4643	HR	0.71 (0.57, 0.89)	0.00254	8.7	0.49	0.1	0.13, 3.88	0.62, 1.19	IV	Critically low
Dai et al., 2017 [161]	Recurrent stroke	Stroke patients	B vitamins	8	10,746	RR	0.63 (0.46, 0.87)	0.00488	63	0.01	0.001	0.26, 1.57	0.81, 1.06	IV	Low
Li et al., 2016 [159]	CAD	Patients with CKD, CVD or stroke, CAD and MI, and so on	Folic acid	25	78,192	RR	1.04 (0.99, 1.09)	0.16	0	0.38	0.53	0.98, 1.09	0.60, 1.82	NS	Low
Li et al., 2016 [159]	CVD	Patients with CKD, CVD or stroke, CAD and MI, and so on	Folic acid	22	74,343	RR	0.94 (0.89, 0.99)	0.0191	20.3	0.04	0.8	0.82, 1.08	0.69, 0.92	IV	Low
Clarke et al., 2011 [162]	All-cause mortality	People with prior CAD, stroke, or end-stage renal disease	B vitamins	8	37,514	RR	1.02 (0.95, 1.09)	0.584	0	0.71	0.71	0.94, 1.11	0.92, 1.18	NS	Critically low
Miller et al., 2010 [163]	All-cause mortality	Pre-existing diseases	Folic acid	12	33,432	RR	1.01 (0.95, 1.06)	0.847	0	0.17	0.72	0.94, 1.07	0.95, 1.12	NS	Critically low
Wang et al., 2015 [164]	Major vascular events	Acute stroke patients	B vitamins	3	11,409	OR	0.87 (0.79, 0.96)	0.0065	0	0.58	0.5	0.46, 1.65	0.79, 1.001	IV	Low
Qin et al., 2011 [165]	Primary cardiovascular outcome	End-stage renal disease or advanced chronic kidney disease	Folic acid	7	3886	RR	0.85 (0.76, 0.96)	0.0091	0	0.85	0.88	0.73, 0.998	0.71, 1.04	IV	Critically low
Fu et al., 2023 [9]	Colorectal cancer	General population	Folic acid	24	6,165,894	RR	0.88 (0.83, 0.92)	3.1E–07	33.4	0.27	0.29	0.76, 1.01	0.76, 1.32	III	Low
Clarke et al., 2011 [162]	Cancer	People with prior CAD, stroke, or end-stage renal disease	B vitamins	5	29,829	RR	1.08 (0.96, 1.20)	0.188	0	0.4	0.75	0.9, 1.29	0.92, 1.24	NS	Critically low
Nigwekar et al., 2016 [166]	Stroke	Dialysis patients	Folic acid	4	1510	RR	0.89 (0.57, 1.40)	0.613	0	0.8	0.66	0.33, 2.39	0.34, 1.55	NS	High
Nigwekar et al., 2016 [166]	All-cause mortality	Dialysis patients	Folic acid	6	2447	RR	1 (0.89, 1.12)	0.984	0	0.11	0.65	0.85, 1.17	0.87, 1.21	NS	High
Nigwekar et al., 2016 [166]	Cardiovascular mortality	Dialysis patients	Folic acid	4	1186	RR	0.93 (0.70, 1.22)	0.585	0	0.27	0.67	0.51, 1.69	0.68, 1.5	NS	High
Qin et al., 2013 [167]	Composite cardiovascular events	Patients with CKD	Folic acid	14	11,323	RR	0.93 (0.87, 0.99)	0.0318	30.7	0.73	0.55	0.87, 1.001	0.85, 1.15	IV	Low
Nigwekar et al., 2016 [166]	Adverse events	Dialysis patients	Folic acid	3	1248	RR	1.12 (0.51, 2.47)	0.774	0	0.69	0.63	0.01, 187	0.41, 3.08	NS	High
Nigwekar et al., 2016 [166]	Myocardial infarction	Dialysis patients	Folic acid	4	1510	RR	1.04 (0.67, 1.62)	0.865	0	0.67	0.64	0.39, 2.77	0.57, 1.91	NS	High
Heinz et al., 2009 [26]	CVD	Dialysis patients	B vitamins	5	710	HR	0.92 (0.75, 1.12)	0.386	51.9	0.99	0.43	0.51, 1.66	0.67, 1.01	NS	Critically low
Jardine et al., 2012 [168]	Composite cardiovascular events	End-stage kidney disease	Folic acid	4	1608	RR	0.91 (0.78, 1.05)	0.178	0	0.59	0.75	0.66, 1.24	0.65, 1.07	NS	Low
Wang et al., 2022 [169]	Dementia	Healthy, MCI, and/or dementia populations	Folate	5	10,514	RR	0.59 (0.45, 0.77)	0.0001	8.9	0.11	0.18	0.32, 1.1	0.35, 1.09	IV	Moderate
Wang et al., 2022 [169]	Dementia	Healthy, MCI, and/or dementia populations	Vitamin B_6_	5	10,525	RR	0.93 (0.72, 1.19)	0.542	0	0.7	0.69	0.62, 1.39	0.53, 1.87	NS	Moderate
Wang et al., 2022 [169]	Dementia	Healthy, MCI, and/or dementia populations	Vitamin B_12_	5	10,539	RR	1.04 (0.83, 1.30)	0.75	0	0.84	0.66	0.72, 1.49	0.81, 2.43	NS	Moderate
Garcia et al., 2018 [170]	Hip fracture	Patients with CVD or colorectal adenomas	Folic acid/vitamin B_12_	4	18,686	RR	1 (0.81, 1.24)	0.989	0	0.29	0.63	0.63, 1.59	0.59, 1.5	NS	Low
Garcia et al., 2018 [170]	Any fracture	Patients with CVD or colorectal adenomas	Folic acid/vitamin B_12_	2	3940	RR	0.86 (0.66, 1.12)	0.259	0	–	0	–	0.58, 1.13	NS	Low
Ruan et al., 2015 [171]	Osteoporotic fracture	Patients with vascular disease	B vitamins	4	26,378	RR	0.75 (0.44, 1.30)	0.308	78.6	0.03	0.59	0.06, 9.53	0.88, 1.24	NS	Critically low

Abbreviations: AMSTAR2, A Measurement Tool to Assess Systematic Reviews version 2; CAD, coronary artery disease; CI, confidence interval; CKD, chronic kidney disease; CVD, cardiovascular disease; eOR, equivalent OR; ESB, excess significance bias; Hcy, homocysteine; HR, hazard ratio; MCI, mild cognitive impairment; MI, myocardial infarction; NS, not significant; OR, odds ratio; PI, prediction interval; RCTs, randomized controlled trials; RR, risk ratio.

Folic acid was specifically evaluated in 13 meta-analyses, whereas vitamins B_6_ or B_12_, or B vitamin complex were analyzed in others. These 26 unique interventional meta-analyses examined the following outcomes including stroke (*n* = 5), coronary artery disease (*n* = 1), CVDs (*n* = 2), composite cardiovascular events in chronic kidney disease (*n* = 1), all-cause mortality (*n* = 2), cardiovascular mortality (*n* = 1), major vascular events (*n* = 2), myocardial infarction (*n* = 1), CRC (*n* = 1), adverse events (*n* = 1), dementia (*n* = 3), fracture (*n* = 3), cancer (*n* = 1), and primary cardiovascular outcome (*n* = 2).

According to the classification criteria for evidence, 23 (88.5%) meta-analyses showed no large heterogeneity (*I*^2^ <50%), and 22 (84.6%) meta-analyses showed neither small-study effects nor excess significant bias. Among these, 8 (30.8%) statistically significant meta-analytical associations met the weak criteria (IV). Table 2 summarizes the results of the interventional meta-analyses. Nine (35%) reported nominally significant summary results at *P* < 0.05 (2 had *P* < 0.001). Only 1 outcome (CRC) was classified as suggestive (class III; *P*=3.11×10^–7^, participants > 20,000, no evidence of small-study effects and excess significant bias, small heterogeneity, but 95% PI including the null and nonsignificance in the largest study). No evidence of classes II or I was observed for the interventional meta-analyses.

### Comparison of findings across the 3 types of studies

AMSTAR II rated most reviews as low or critically low in quality. Critical domains related to study exclusion were inadequately addressed in most reviews. The quality of included meta-analyses, as assessed by AMSTAR2, was high in 1 meta-analysis, moderate in 2, low in 60, and critically low in 53 (Supplemental Table 5).

For the same outcomes, comparisons between observational and MR studies were limited for many outcomes due to data unavailability. A total of 25 outcomes were reported in both study types (Table 3). Among these, 3 outcomes (ischemic stroke, type 2 diabetes, and amyotrophic lateral sclerosis) were not significant in both observational meta-analyses and MR studies. Of the 25 outcomes, 6 MR studies (essential hypertension, small-vessel occlusion stroke, stroke, schizophrenia, type 2 diabetic nephropathy, metabolic syndrome) showed significance (*P* < 0.01), and 9 (coronary artery disease incidence, ischemic stroke, small-vessel occlusion stroke, stroke, multiple sclerosis, schizophrenia, type 2 diabetes, metabolic syndrome, nonalcoholic fatty liver disease) had statistical power >80%. Collectively, 4 outcomes (stroke, small-vessel occlusion stroke, schizophrenia, and metabolic syndrome) demonstrated both *P* < 0.01 and high statistical power (>80%), suggesting that Hcy is a key causal risk factor, supported by observational and MR studies, for stroke, small-vessel occlusion stroke, schizophrenia, and metabolic syndrome.

**TABLE 3:** Summary of evidence grading and comparison of outcomes across 2 or more study types for diseases.

Outcomes	Observational meta-analyses			MR studies		OM-MR concordance		Interventional meta-analyses			
	ES (95% CI)	*P* value	Lev	ES (95% CI)	Evidence^2^	*P*	Significance	ES (95% CI)	*P* value	Lev	Treatment
Calcific aortic valve disease	2.83 (1.86, 4.32)	1.4E-06	III	1.14 (0.86, 1.5)	*P* = 0.36, power = 0.475	<0.001	MR: weak				
Heart failure	8.43 (2.34, 30.39)	0.0011	IV	0.96 (0.88, 1.05)	*P* = 0.37, power = 0.642	0.001	MR: weak				
Atrial fibrillation	2.79 (1.18, 6.59)	0.019	IV	0.96 (0.91, 1.01)	*P* = 0.098, power = 0.769	0.015	MR: weak				
Coronary artery disease	1.21 (1.11, 1.33)	2.8E-05	III	1.05 (0.96, 1.15)	*P* = 0.26, power = 0.98	0.026	MR: weak	1.04 (0.99, 1.09)	0.16	NS	Folic acid
Essential hypertension	1.35 (1.03, 1.77)	0.0287	IV	1.32 (1.22, 1.49)	*P* = 2.2E^-04^,^1^ power = 0.76	0.874	MR: weak				
Abdominal aortic aneurysm	3.46 (1.43, 8.38)	0.006	IV	1.11 (0.92, 1.35)	*P* = 0.29, power = 0.379	0.014	MR: weak				
Ischemic stroke	1.06 (1.00, 1.12)	0.063	NS	1.10 (0.98, 1.23)	*P* = 0.11, power = 0.833	0.547	Meta and MR: weak				
Intracerebral hemorrhage	2.94 (2.45, 3.47)	8.9E-38	IV	1.09 (0.89, 1.34)	*P* = 0.41, power = 0.367	<0.001	MR: weak				
Small-vessel occlusion stroke	6.89 (3.55, 13.36)	1.1E-08	IV	1.34 (1.13, 1.58)	*P* = 6.7E-04, power = 0.998	<0.001	Both sig.; direction is inconsistent				
Stroke	1.06 (1.01, 1.12)	0.025	IV	1.11 (1.03, 1.20)	*P* = 0.008, power = 1	**0.35**	Both sig.; direction is consistent	0.89 (0.81, 0.97)	0.012	IV	Folic acid
Peripheral arterial disease	2.24 (1.65, 3.05)	2.4E-07	II	1.06 (0.91, 1.23)	*P* = 0.49, power = 0.291	<0.001	MR: weak				
Alzheimer’s disease	1.74 (1.31, 2.32)	0.00012	IV	1.08 (0.96, 1.22)	*P* = 0.198, power = 0.623	0.002	MR: weak				
Parkinson’s disease	4.19 (3.03, 5.78)	3.1E-18	II	0.99 (0.85, 1.14)	*P* = 0.837, power = 0.095	<0.001	MR: weak				
Amyotrophic lateral sclerosis	2.47 (0.91, 6.74)	0.077	NS	1.09 (0.95, 1.24)	*P* = 0.235, power = 0.692	0.11	Meta and MR: weak				
Multiple sclerosis	3.01 (1.69, 5.37)	1.9E-04	III	0.78 (0.64, 0.94)	*P* = 0.011, power = 1	<0.001	Both sig.; direction is inconsistent				
Schizophrenia	2.98 (1.94, 4.57)	5.5E-07	II	1.11 (1.03, 1.20)	*P* = 0.0027, power = 0.832	<0.001	Both sig.; direction is inconsistent				
Autism spectrum disorder	3.56 (1.76, 7.22)	0.0004	III	1.03 (0.92, 1.15)	*P* = 0.63	0.001	MR: weak				
Type 2 diabetes	6.58 (0.56, 77.75)	0.135	NS	1.08 (0.95, 1.21)	*P* = 0.25, power = 0.998	0.152	Meta and MR: weak				
Type 2 diabetic nephropathy	7.66 (3.17, 18.56)	6.4E-06	IV	3.86 (1.21, 2.05)	*P*<0.001, power = 0.613	0.145	MR: weak				
Metabolic syndrome	1.52 (1.30, 1.78)	2.9E-07	III	0.72 (0.50, 0.94)	*P*<0.001, power = 1	<0.001	Both sig.; direction is inconsistent				
Nonalcoholic fatty liver disease	2.00 (1.54, 2.60)	1.8E-07	III	1.21 (1.01, 1.43)	*P* = 0.041, power = 0.989	0.003	Both sig.; direction is inconsistent				
Cardiovascular mortality	1.32 (1.09, 1.60)	0.00533	IV	1.76 (0.54, 5.77)	*P*>0.05, power = 0.401	0.638	MR: weak				
All-cause mortality	1.79 (1.51, 2.12)	1.3E-11	II	0.99 (0.62, 1.57)	*P* > 0.05, power = 0.03	0.019	MR: weak	1.01 (0.95, 1.06)	0.85	NS	B vitamins
Fracture	1.58 (1.28, 1.95)	1.8E-05	III	0.97 (0.88, 1.07)	*P* > 0.05, *P* = 0.562	<0.001	MR: weak	0.86 (0.66, 1.12)	0.26	NS	Folic acid/vit B_12_
Chronic kidney disease	2.18 (1.71, 2.77)	2.3E-10	II	1.24 (1.07, 1.44)	*P* < 0.05	<0.001	MR: uncertain				
Dementia	1.65 (1.37, 1.98)	7.5E-08	IV					0.59 (0.45, 0.77)	1.4E-04	IV	Folic acid
Colorectal cancer	1.27 (1.14, 1.41)	1E-05	III					0.88 (0.83, 0.92)	3E-07	III	Folic acid

Abbreviations: CI, confidence interval; ES, effect size; Lev, level; MR, Mendelian randomization; NS, not significant; sig, significant; OM, observational meta-analysis.

1 Indicates an estimated value due to no report in original study.

2 Strong: *P* < 0.01 and power >0.8.

To enhance the credibility and interpretability of the findings, we performed consistency tests between observational and MR studies. Seventeen outcomes showed *P* values <0.05, indicating significant inconsistency between the parallel studies. These outcomes included calcific aortic valve disease, heart failure, atrial fibrillation, coronary artery disease, abdominal aortic aneurysm, intracerebral hemorrhage, small-vessel occlusion stroke, peripheral arterial disease, Alzheimer's disease, Parkinson's disease, multiple sclerosis, schizophrenia, autism spectrum disorder, metabolic syndrome, nonalcoholic fatty liver disease, all-cause mortality, and fracture (Table 3 and Supplemental Figure 1). The interaction analyses testing for differences in estimates between parallel studies revealed 7 outcomes with directionally consistent results (*P* for interaction >0.05). Among these, stroke was the only outcome that demonstrated consistency between observational meta-analysis and MR study while also showing statistically significant and strong effect sizes (meta-analysis: OR = 1.06, 95% CI = 1.01, 1.12, class IV; MR study: OR = 1.11, 95% CI = 1.03, 1.20, *P* = 0.008, power = 1; *P* for interaction = 0.35). This indicates that Hcy is a consistently identified causal risk factor for stroke, but not for other disease outcomes when comparing observational and MR studies.

Next, we compared the consistent results from observational studies and MR studies to those from meta-analyses of RCTs. Four disease outcomes (coronary artery disease, stroke, all-cause mortality, and fracture) were examined across the 3 types of studies. Only stroke demonstrated identical conclusions (effect and level of statistical significance/direction) across all 3 study types, further supporting that Hcy, an effective modifiable intervention factor, is causally associated with stroke, and that Hcy-lowering treatment with folic acid helps reduce the risk of stroke.

Although MR studies were unavailable on dementia and CRC, observational and interventional meta-analyses were conducted for both outcomes. Unexpectedly, Hcy showed significant associations with both dementia and CRC risks (*P =* 7.5 × 10^–8^, 1 × 10–^5^, respectively). Furthermore, Hcy-lowering treatment with folic acid reduced risks for both diseases (class IV and III evidence, respectively), which seems to imply the causal effects of Hcy on both the diseases.

## Discussion

### Main findings and possible explanations

The present umbrella review synthesized an extensive body of literature on Hcy, including 135 meta-analyses testing observational associations between Hcy and 93 unique physical disease outcomes, 106 MR studies investigating causal associations with 81 unique outcomes, and 26 interventional meta-analyses examining the effects of Hcy-lowering treatments. This umbrella review is the first to evaluate the broad impact of Hcy and Hcy-lowering interventions across diverse health outcomes by integrating evidence from observational, interventional meta-analyses, and MR studies, using established grading criteria controlling for bias.

Although most associations were statistically significant (*P* < 0.05) under random-effect models in observational meta-analyses, only the association for digestive tract cancer met criteria for convincing (class I). Ten outcomes showed highly suggestive evidence (class II), including ulcerative colitis, Behçet’s syndrome, rheumatoid arthritis, schizophrenia, polycystic ovary syndrome, cerebral small-vessel disease, peripheral arterial disease, chronic kidney disease, first-time stroke, all-cause mortality (ranked by effect size from largest to smallest).

Notably, a large proportion (80%) of these meta-analyses displayed substantial heterogeneity (*I*^2^ > 50%), and one-third showed evidence of small-study effects and/or excess significance bias. Heterogeneity may arise from biased results but could also reflect true variations across studies, such as differences in study design or categorization of Hcy levels (tertiles/quartiles/quintiles/sextiles). Thus, caution is warranted when interpreting these associations, particularly when heterogeneity is large or small-study effects are present. Given evidence from prospective compared with case-control study comparisons and established biological pathways where disease may elevate Hcy levels, some associations initially classified as class II (e.g. Alzheimer’s disease) were no longer highly suggestive when accounting for reverse causality.

The comparison of the findings from MR studies and meta-analyses of observational studies with interventional meta-analyses indicated a strong association between Hcy (or Hcy-lowering) with stroke. The direction of the effects of Hcy and Hcy-lowering treatment on stroke is consistent across the 3 types of studies, though credibility assessments of Hcy and Hcy-lowering treatment (with B vitamins supplementation) for stroke yielded low-grade evidence. These findings suggest that Hcy is a key causal risk factor for stroke, and Hcy-lowering treatment confers long-term benefits in the prevention of this disease. In addition, significant associations of Hcy with small-vessel occlusion stroke, schizophrenia, and metabolic syndrome were observed in both observational meta-analyses and MR studies; however, interventional meta-analyses demonstrated minimal or no preventive/therapeutic effects of Hcy-lowering interventions for these diseases, highlighting the need for future RCTs.

The idea of whether or not lowering Hcy can prevent further stroke in patients who have already suffered cardiovascular events has been yet controversial. To be disappointing, trials such as Vitamin Intervention for Stroke Prevention (VISP) [4], Heart Outcomes Prevention Evaluation (HOPE) [172], and Norwegian Vitamin Trial [173] concluded that Hcy-lowering could not prevent secondary strokes and other CVD events. A meta-analysis [10] pooling data from 3 trials (VISP, HOPE-2, VITAmins TO Prevent Stroke (VITATOPS)) found an ∼30% reduction in stroke risk among 4643 vascular patients taking B vitamins but not taking antiplatelet drugs. This suggests potential attenuation of B vitamin benefits in those receiving antiplatelet agents or lipid-lowering medications, with additional evidence indicating the therapeutic efficacy of B vitamins may be modulated by omega-3 fatty acid status and adequate micronutrient supplementation [174,175]. The China Stroke Primary Prevention Trial (CSPPT) trial [176], the only large-scale primary prevention trial of folic acid-based Hcy-lowering therapy, was conducted in China. In hypertensive adults without a history of stroke or myocardial infarction, enalapril combined with folic acid significantly reduced risk of first stroke compared with enalapril alone. A post hoc CSPPT analysis further linked greater Hcy reduction to lower first-stroke risk [177]. A prior umbrella review [178] also suggested folic acid’s protective effect against stroke (low certainty evidence), aligning with the causal role of Hcy-lowering in stroke prevention. This umbrella review comprehensively evaluated Hcy’s association with CVDs across 3 study types, confirming stronger evidence for the causal effect of Hcy on stroke (based on the concordant effect direction and statistical significance) than on coronary artery disease or other cardiovascular events.

HHcy is multifactorial [179], and high plasma levels of total Hcy are derived from the interaction between genetic and environmental factors. Genetic abnormalities are involved in several enzymes of Hcy metabolism, such as those causing CβS deficiency, or polymorphisms of MTHFR-C677T [180]. Marked elevations are observed in homozygous CβS deficiency, whereas more moderate increases occur in heterozygous CβS deficiency and MTHFR C677T, which are usually associated with mild HHcy [181]. In addition to genetic and environmental factors, physiological conditions must be considered. Clinical studies have documented elevated Hcy levels in 85% of patients with chronic kidney disease [182] and in the euthyroid population with impaired sensitivity to thyroid hormones [183]. The detrimental effect of HHcy is significantly influenced by the overall cardiovascular redox state, particularly its antioxidant capacity, as measured by glutathione peroxidase-1. Several mechanisms have been proposed for Hcy’s role in vascular disease pathogenesis. Hcy can cause endothelial injury, DNA dysfunction, smooth muscle cell proliferation, oxidative stress, reduced glutathione peroxidase activity, impaired nitric oxide synthase function, and inflammation [184]. Our umbrella review supports the beneficial effects of Hcy-lowering treatments (e.g. folic acid) on stroke, which can largely be attributed to the detrimental role of excessive Hcy in endothelial dysfunction [185,186] and prothrombosis [187,188].

Digestive tract cancer was the only outcome with convincing evidence. The association between Hcy and digestive tract cancer was positive but had a mild effect (eOR = 1.27). For CRC, a subtype of digestive tract cancer, no between-study heterogeneity was observed, and no evidence of small-study effects or excess significance bias was identified (class III); only the *P* value did not meet the threshold of convincing evidence (*P* = 0.0000103>10^–6^). Additionally, neither the risk of esophagogastric nor gastric cancers was significantly associated with Hcy, but the effect size for CRC matched that of digestive tract cancer (both eORs = 1.27). This implies that this convincing evidence might specifically reflect the association of CRC [189].

Several mechanisms explain the convincing association of Hcy with digestive tract cancer (causality cannot be inferred due to a lack of MR studies). Cancer cells highly depend on the methionine cycle, resulting in the production of large amounts of Hcy [190]. Most chemotherapy drugs targeting folate metabolism [191] are anti-folate agents, and folate deficiency can elevate Hcy levels in these patients [192]. Patients undergoing chemotherapy exhibit increased blood levels of Hcy. The findings suggest that HHcy is closely linked to cancer, but observational studies may reflect reverse causality. Hcy might exert a causal effect on digestive tract cancer, in particular CRC, which is supported by our umbrella review on RCTs (folic acid decreases risk of CRC, suggestive evidence). Site-specific (colorectal) mechanisms are supported by the following. The association between Hcy and digestive tract cancer varies by anatomical site. It has been proposed that an elevated Hcy level is responsible for many pathological mechanisms, such as oxidative stress [193], endothelial dysfunction, and colorectal polyp risk [194]. In the colon, Hcy potentiates hydrogen sulfide (H_2_S)-driven carcinogenesis, a process amplified by sulfate-reducing bacteria abundant in this region [195]. Hcy-linked ulcerative colitis increases CRC risk by 2- to 3-fold, establishing a pathogenic cascade unique to the colon and absent in upper gastrointestinal (GI) cancers [196]. Folic acid supplementation reduces CRC but no other GI cancers, further suggesting site-specific Hcy biology. The largest effect estimate among class II evidence was observed for ulcerative colitis, further implicating Hcy in CRC pathogenesis through inflammatory precursor pathways. However, future experimental and MR studies are needed to confirm Hcy’s potential causal role.

The first umbrella review on Hcy was published in 2021 [197]. Li et al. assessed the relationship between serum Hcy and primary glaucoma risk and reported weak evidence (class IV), which is consistent with the findings of the present review on glaucoma. Zhang et al. [198] reported that Hcy was associated with all-cause dementia (class II), but the umbrella review included case-control rather than prospective cohort studies. For Alzheimer’s disease, a recent review of meta-analyses from case-control studies revealed highly suggestive evidence (class II) [199].

### Clinical implications and future research

Given the clinically highly relevant findings, the prevention and/or treatment of HHcy has great potential to improve overall health and outcomes based on a large number of positive associations of Hcy with health outcomes with robust evidence (ranging from class I to III). Our umbrella review directly informs the prioritization of these approaches and associated resources according to evidence-based potential preventive gains. Because a wide range of health outcomes have been associated with high Hcy levels, there is a renewed interest in whether individuals with asymptomatic HHcy should receive treatment or be monitored for the prevention of these diseases. Current guidelines suggest that drug-based prevention/treatment of HHcy is strongly related to stroke prevention.

We identified only 1 convincing association from observational studies (with digestive tract cancer). Although CRC lacked causal evidence from MR studies, the observed association between HHcy and CRC in observational studies likely reflects HHcy’s role as a biomarker of folate deficiency—a well-established risk factor for colorectal carcinogenesis. This interpretation aligns with evidence from RCTs showing that Hcy-lowering interventions via folic acid supplementation [9] reduce CRC risk, suggesting that the benefits of such interventions may stem from correcting folate status rather than modulating Hcy per se. Consequently, although Hcy-lowering strategies (e.g. folate fortification) hold promise for CRC prevention, this effect is mediated through folate restoration rather than direct causality of HHcy.

For future research, more efforts are required to address some concerns. Whether a causal effect of Hcy on CRC exists has not been comprehensively investigated in MR studies. How Hcy affects the progression and pathogenesis of CRC, as well as the underlying mechanisms, might be worth further investigation. In addition, in view of the largely discordant evidence across the 3 types of studies, better study design coordinated by large international consortia might assist in deciding whether the lack of replication of highly suggestive findings is owing to low power to detect moderate-to-small effects or owing to actual null effects. It has been shown that folic acid supplementation can lower Hcy levels by 25% [172]. There is reasonable biological plausibility for the effect of folic acid independent of Hcy-lowering. Thus, efforts to investigate whether other Hcy-lowering agents or measures have the same effect as folic acid will help determine whether these effects are truly due to the reduction of Hcy itself rather than other properties of the agents (e.g. folate also stimulates cell proliferation and might promote the progression of atherosclerosis) [200].

### Strengths and limitations of this review

There are several strengths in the present umbrella review worth mentioning. First, the associations between Hcy and a wide spectrum of health outcomes were systematically and thoroughly assessed by incorporating data from observational and interventional meta-analyses, and MR studies. We calculated some additional metrics and applied well-defined criteria to assess the credibility of the associations and the statistical power of MR studies. Second, the present umbrella review integrated the results of MR studies and observational studies to avoid the inevitable bias or reverse causality of observational studies. The results across 3 types of studies consistently suggest that Hcy is a causal and modifiable risk factor for stroke. Therefore, we consider that the apparent beneficial effect of B vitamin supplementation (with folic acid having a more definite effect [49]) on stroke likely represents neither an overestimate of the real effect nor a spurious result due to the play of chance [201]. Third, an additional strength was the in-depth screening of primary studies included in each meta-analysis to selectively further analyze only data reflecting prospective observational associations. This approach mitigated the reverse causality bias and ensured the temporality of the examined associations, where exposures always preceded the event investigated.

We acknowledged several limitations of our umbrella review when interpreting these findings. First, the inherent limitations are subjected to evidence from existing reviews, and residual confounding cannot be ruled out despite including some large sample, high-quality cohort studies. Some reviews may have flaws in design, data extraction, or analysis, which could affect the reliability of the umbrella review’s conclusions. If an included meta-analysis contains incorrect data, the umbrella review incorporating it may also yield erroneous inferences, making it challenging to accurately combine and compare results [202]. Additionally, although the outcomes with class I or II evidence met the criteria for credibility assessment in observational meta-analyses, it would be inadvisable to conclude causation on this basis alone, due to the inherent limitations of unmeasured confounding, undetected bias, or reverse causality in observational studies. Second, the findings of this umbrella review may not apply to all populations or settings, as the included studies were conducted in specific geographical locations, with particular patient groups, or under certain conditions. For example, analyses of clinical trials on Hcy-lowering treatments might only include studies from developed countries, limiting generalizability to populations with different genetic profiles, healthcare systems, and patient characteristics. Furthermore, some included studies may be outdated by the time an umbrella review is published, and ongoing and future research could alter its conclusions. Third, most genetic studies to date have focused on European populations. Designing arrays based on more globally diverse populations will be crucial to reducing systematic European bias. Whether these results are generalizable remains unknown, and future genetic studies should prioritize diverse ancestries to resolve this bias. MR analyses also have low statistical power when genetic variants explaining a risk factor account for only a small percentage of variability, as is often the case.

### Conclusion

Despite hundreds of systematic reviews, meta-analyses, and MR studies exploring multiple health outcomes, the most convincing evidence for a clear role of Hcy level exists only for digestive tract cancer without bias or other confounding factors. Concordant evidence between observational meta-analyses and MR studies with significant effects exists for stroke, and interventional trials further confirm a definite causal role of Hcy levels in stroke. Prevention of stroke, particularly by targeting HHcy, can reduce the incidence and recovery of adverse clinical outcomes in physical diseases. However, considering the existence of high risk bias in original meta-analyses, the finding for stroke may not be robust enough, and needs confirmation in future studies. Our comprehensive umbrella review will help prioritize health outcomes related to Hcy levels for future research and clinical management.

## Author contributions

The authors’ responsibilities were as follows – YH, YZ, FZ: performed the literature search, screening, and data extraction; XX, NG: conducted the data analysis; FZ, WC, YZ: designed the figures and tables and drafted the initial manuscript; FZ, YH: finalized the writing; FZ: responsible for the submission decision; and all authors: full access to all study data, participated in data interpretation, critically revised the manuscript for intellectual content, and approved the final version

## Data availability

All data included in this umbrella review were extracted from publicly available systematic reviews.

## Funding

The work was supported by the National Natural Science Foundation of China (no. 32160212).

## Conflict of interest

The authors report no conflicts of interest.
