# Supplementary material for: Homocysteine and Multiple Health Outcomes: An Outcome-Wide Umbrella Review of Meta-analyses and Mendelian Randomization Studies
Source: Adv Nutr. 2025 Apr 25;16(6):100434. doi: 10.1016/j.advnut.2025.100434 (PMC12144516; doi:10.1016/j.advnut.2025.100434)
Supplement: multimedia component 1 [file mmc1.docx]

**Homocysteine and multiple health outcomes: an outcome-wide umbrella review of meta-analyses and Mendelian randomization studies**

**Futao Zhou**

**Supplementary figure and table legends**

**Supplementary Table 1.** Keywords and search strategy used in the umbrella review.

**Supplementary Table 2.** Characteristics and quantitative synthesis of meta-analyses of observational studies reporting associations of Hcy with health outcomes.

**Supplementary Table 3.** Summary of credibility assessment for characteristics and quantitative synthesis of meta-analyses of observational studies reporting associations of Hcy with multiple health outcomes.

**Supplementary Table 4**. Characteristics and statistical power of primary MR studies reporting causal associations of Hcy with multiple health outcomes.

**Supplementary Table 5**. Methodological quality assessment of included studies using AMSTAR2.

**Supplementary Figure 1.** Consistency between meta-analyses of observational studies and MR studies for the same health outcome. The P value is for interaction test between study types.

**Supplementary Table 1.** Keywords and search strategy used in the umbrella review (Pubmed, EMBASE and Cochrane library databases from inception to May 7, 2024)

| Items | Terms | |
| --- | --- | --- |
| Homocysteine terms | homocysteine[Title/Abstract] | hyperhomocysteinemia[Title/Abstract] |
| Review terms | meta-analysis[Title/Abstract] | meta-analyses[Title/Abstract] |
|  | “systematic review”[Title/Abstract] | “Mendelian Randomization”[Title/Abstract] |
|  | “Mendelian Randomi*”[Title/Abstract] | “systematic review”[Title/Abstract] |

**Supplementary Table 2. Characteristics and quantitative synthesis of meta-analyses of observational studies reporting associations of Hcy with health outcomes.**

| **Meta-review** | **Outcome** | **Population** | **Study design** | **Comparison** | **n_st** | **n_cases** | **n_partic** | **Metric** | **Original estimate** | | **eOR (95% CI)** | | **P-value** | **PI** | ***I*^2^** | **SSE_p** | **ESB_p** | **Level** | **Selection** |
| --- | --- | --- | --- | --- | --- | --- | --- | --- | --- | --- | --- | --- | --- | --- | --- | --- | --- | --- | --- |
| **Cardiocelebreovascular diseases** | | | |  |  |  |  |  |  |  |  |  |  |  |  |  |  |  |  |
| Li H, 2022 [1] | First-time stroke | General | Case–ctrl or cohort | Patients vs ctrls | 21 | 2821 | 38016 | OR | 2.0 (1.6, 2.4) | | 1.97 (1.58, 2.45) | | 1.3E-09 | 0.9, 4.3 | 55.3 | 0.56 | 0.07 | II | Yes |
| Clarke, 2002 [2] | Stroke | General | Prospective | 25% lower Hcy | 8 | 463 | 2063 | OR | 0.8 (0.6, 0.96) | | 0.76 (0.60, 0.96) | | 0.0215 | 0.4, 1.9 | 47 | 0.42 | 0.18 | IV | No |
| Clarke, 2002 [2] | Stroke | General | Retrospective | 25% lower | 3 | 344 | 944 | OR | 0.9 (0.7, 1.0) | | 0.86 (0.73, 1.01) | | 0.0611 | 0.3, 2.4 | 21.9 | 0.96 | 0.80 | NL | No |
| Wu, 2020 [3] | Stroke | General | Prospective | Highest vs lowest | 7 | 644 | 11705 | RR | 1.6 (1.2, 2.2) | | 1.63 (1.21, 2.21) | | 1.5E-3 | 0.8, 3.4 | 38.8 | 0.21 | 0.23 | IV | No |
| Wu, 2020 [3] | Stroke | General | Prospective | 1-unit increase | 6 | 523 | 10914 | RR | 1.1 (1.0, 1.1) | | 1.06 (1.01, 1.12) | | 0.0247 | 0.9, 1.2 | 59 | 0.30 | 0.04 | IV | Yes |
| Zhang, H, 2021 [4] | Recurrent stroke | Acute ischemic stroke patients | Prospective | Highest vs lowest | 5 | 3004 | 17866 | RR | 1.3 (0.96, 1.8) | | 1.30 (0.96, 1.77) | | 0.09 | 0.5, 3.6 | 63.3 | 0.33 | 0.63 | NL | Yes |
| Zhou, 2018 [5] | Intracerebral hemorrhage | General | Case-ctrl | Patients vs ctrls | 9 | 667 | 3167 | SMD | 0.6 (0.5, 0.7) | | 2.94 (2.50, 3.47) | | 8.91E-38 | 2.4, 3.6 | 0 | 0.53 | 0.40 | IV | Yes |
| Fu, 2015 [6] | Cerebral infarction | General | Case-ctrl | Patients vs ctrls | 13 | 1206 | 2408 | SMD | 1.5 (0.2, 2.8) | | 15.68 (1.49, 164.82) | | 0.0219 | 0.001, 276131.0 | 95.5 | 0.005 | 0.048 | IV | Yes |
| Lauw, 2013 [7] | Cerebral venous thrombosis | General | Case-ctrl | Patients vs ctrls | 4 | 212 | 972 | OR | 3.0 (1.2, 7.2) | | 2.96 (1.22, 7.17) | | 0.0165 | 0.06, 141.5 | 70.3 | 0.45 | 0.28 | IV | Yes |
| Zhang, 2020 [8] | Small-vessel occlusion stroke | Chinese | Case-ctrl | Patients vs ctrls | 13 | 781 | 1652 | SMD | 1.1 (0.7, 1.4) | | 6.89 (3.55, 13.36) | | 1.1E-08 | 0.5, 94.4 | 88.8 | 0.74 | 0.49 | IV | Yes |
| Pinzon, 2023 [9] | Ischemic stroke | General | Case–ctrl or cohort | Patients vs ctrls | 33 | 6946 | 12528 | SMD | 1.1 (0.6, 1.7) | | 7.72 (2.96, 20.14) | | 3E-05 | 0.02, 2462.6 | 97.4 | 0.002 | <.01 | III | No |
| Wu, 2020 [3] | Ischemic stroke | General | Prospective | Highest vs lowest | 7 | 595 | 11656 | RR | 1.6 (1.2, 2.1) | | 1.59 (1.18, 2.15) | | 2.5E-03 | 0.8, 3.1 | 35.7 | 0.28 | 0.12 | IV | No |
| Holmen, 2021 [10] | Ischemic stroke | General | Case-ctrl or cross-sectional | per 5-unit increase | 6 | 781 | 2081 | OR | 1.4 (1.3, 1.6) | | 1.43 (1.28, 1.61) | | 1.27E-09 | 1.2, 1.7 | 0 | 0.045 | 0.027 | IV | No |
| Wu, 2020 [3] | Ischemic stroke | General | Prospective | 1-unit increase | 6 | 523 | 10914 | RR | 1.1 (1, 1.1) | | 1.06 (0.997, 1.12 | | 0.0632 | 0.9, 1.3 | 58.7 | 0.29 | 0.33 | NL | Yes |
| Zhang, H, 2021 [4] | Poor functional outcome in acute ischemic stroke | Patients with acute ischemic stroke |  | Highest vs lowest | 4 | 529 | 4467 | RR | 1.8 (0.6, 5.4) | | 1.84 (0.63, 5.40) | | 0.267 | 0.01, 256.9 | 76.8 | 0.20 | 0.61 | NL | No |
| Piao, 2018 [11] | Cerebral small vessel disease | General | Case-ctrl or cross-sectional | Patients vs ctrls | 20 | 1987 | 5088 | SMD | 0.5 (0.3, 0.7) | | 2.46 (1.85, 3.26) | | 6.15E-10 | 0.7, 8.6 | 78.5 | 0.05 | <.01 | II | No |
| Tian, 2023 [12] | Intracranial atherosclerotic stenosis | Stroke-free populations | Case-ctrl | Patients vs ctrls | 4 | 1181 | 7053 | SMD | 0.5 (0.1, 0.9) | | 2.67 (1.29, 5.51) | | 0.0079 | 0.08, 85.1 | 92.7 | 0.17 | <.01 | IV | Yes |
| Bautista, 2002 [13] | Cardiovascular disease | General | Prospective | First occurence in patients vs ctrls | 15 | 2412 | 9717 | RR | 1.4 (1.1, 1.6) | | 1.37 (1.15, 1.64) | | 5.5E-04 | 0.8, 2.4 | 61.6 | 0.31 | 0.28 | III | Yes |
| Wu, 2018 [14] | Calcific aortic valve disease | General | Case-ctrl | Patients vs ctrls | 10 | 3030 | 6349 | SMD | 0.6 (0.3, 0.8) | | 2.83 (1.86, 4.32) | | 1.4E-06 | 0.6, 12.8 | 91.3 | 0.98 | 0.68 | III | Yes |
| Rong, 2020 [15] | Atrial fibrillation | General | Case-ctrl | With vs without recurrence | 3 | 101 | 423 | SMD | 0.6 (0.3, 0.9) | | 3.13 (1.85, 5.28) | | 2E-05 | 0.04, 264 | 29.3 | 0.79 | 0.55 | IV | No |
| Rong, 2020 [15] | Atrial fibrillation | General | Case-ctrl | Patients vs ctrls | 7 | 2794 | 3699 | SMD | 0.6 (0.1, 1.0) | | 2.79 (1.18, 6.59) | | 0.0194 | 0.1, 54.6 | 91.4 | 0.28 | <.01 | IV | Yes |
| Clarke, 2002 [2] | Ischemic heart disease | General | Retrospective | 25% lower Hcy | 12 | 2496 | 4896 | OR | 0.6 (0.5, 0.7) | | 0.62 (0.54, 0.72) | | 1.09E-11 | 0.4, 0.99 | 72 | 0.05 | 0.001 | II | No |
| Clarke, 2002 [2] | Ischemic heart disease | General | Prospective | 25% lower Hcy | 11 | 1968 | 4168 | OR | 0.8 (0.7, 0.9) | | 0.82 (0.74, 0.91) | | 3E-04 | 0.6, 1.1 | 51.9 | 0.72 | 0.55 | III | Yes |
| Unadkat, 2024 [16] | Coronary heart disease | General | Case-ctrl | Patients vs ctrls | 59 | 9381 | 21569 | SMD | 0.7 (0.6, 0.9) | | 3.77 (2.75, 5.16) | | 1.26E-16 | 0.3, 42 | 94.4 | 0.0004 | <.01 | II | No |
| Humphrey, 2008 [17] | Coronary heart disease | General adult population without known CHD | Nested case-ctrl or cohort | 5-unit increase | 18 | 3035 | 25909 | RR | 1.2 (1.1, 1.3) | | 1.21 (1.11, 1.33) | | 3E-05 | 0.95, 1.5 | 34.3 | 0.20 | 0.39 | III | Yes |
| Wang, 2022 [18] | Coronary heart disease | General | Case-ctrl or cohort | 5-unit increase | 9 | 2161 | 6398 | RR | 1.2 (1.1, 1.4) | | 1.23 (1.09, 1.40) | | 7E-04 | 0.98, 1.5 | 27.7 | 0.64 | <.01 | III | No |
| Jin, 2021 [19] | Heart failure | General | Case-ctrl | Patients vs ctrls | 12 | 1530 | 5506 | SMD | 1.2 (0.5, 1.9) | | 8.43 (2.34, 30.4) | | 1.1E-03 | 0.05, 1509.4 | 96.6 | 0.003 | <.01 | IV | Yes |
| Cao, 2014 [20] | Abdominal aortic aneurysm | General | Case-ctrl | Patients vs ctrls | 7 | 1410 | 6445 | OR | 3.5 (1.4, 8.4) | | 3.46 (1.43, 8.38) | | 0.006 | 0.2, 68.8 | 92.9 | 0.05 | <.01 | IV | Yes |
| Wang, H, 2022 [21] | Peripheral arterial disease | General | Cohort, case–ctrl, cross-sectional | Patients vs ctrls | 16 | 2035 | 11812 | SMD | 0.4 (0.3, 0.6) | | 2.24 (1.65, 3.05) | | 2.4E-07 | 0.7, 7.7 | 82.5 | 0.18 | 0.02 | II | Yes |
| Zhong, 2017 [22] | Essential hypertension | General | Prospective and retrospective | Highest vs lowest | 11 | 4830 | 16571 | OR | 1.4 (1.0, 1.8) | | 1.35 (1.03, 1.77) | | 0.0287 | 0.6, 3.2 | 82.8 | 0.15 | 0.05 | IV | Yes |
| **Neurology and psychiatry** | | | |  |  |  |  |  |  |  |  |  |  |  |  |  |  |  |  |
| Zhang, 2022 [23] | Alzheimer's disease | General | Case-ctrl | Patients vs ctrls | 33 | 2092 | 4262 | SMD | 0.7 (0.6, 0.9) | | 3.75 (2.81, 5.02) | | 5.35E-19 | 0.8, 17.8 | 78.2 | 0.00063 | <.01 | II | No |
| Zhou, 2019 [24] | Alzheimer's disease | General | Prospective | Highest vs lowest | 8 | 385 | 4174 | RR | 1.7 (1.3, 2.3) | | 1.74 (1.31, 2.32) | | 1.2E-04 | 0.8, 3.7 | 58.4 | 0.0045 | <.01 | IV | Yes |
| Zhou, 2019 [24] | All-cause dementia | General | Prospective | Highest vs lowest | 10 | 699 | 6810 | RR | 1.6 (1.4, 2) | | 1.65 (1.37, 1.98) | | 7.5E-08 | 1.2, 2.3 | 14.9 | 0.012 | 0.18 | IV | Yes |
| Liu, 2023 [25] | Parkinson's disease | General | Case-ctrl | Patients vs ctrls | 50 | 4456 | 8323 | SMD | 0.8 (0.6, 1) | | 4.19 (3.03, 5.78) | | 3.13E-18 | 0.5, 38.8 | 91.8 | 0.025 | <.01 | II | No |
| Liu, 2023 [25] | Parkinson's disease | Untreatment | Case-ctrl | Patients vs ctrls | 10 | 1078 | 2181 | SMD | 0.3 (0.2, 0.4) | | 1.69 (1.29, 2.23) | | 1.7E-04 | 0.9, 3.1 | 15 | 0.91 | 0.5 | III | Yes |
| Dong, 2020 [26] | Parkinson's disease | Chinese | Case-ctrl | Patients vs ctrls | 26 | 1765 | 2918 | SMD | 1.2 (0.9, 1.5) | | 8.48 (4.94, 14.56) | | 8.34E-15 | 0.5, 138.9 | 90.2 | 0.0063 | 0.34 | III | Yes |
| Periñán, 2023 [27] | Cognitive impairment | Patients with Parkinson's disease | Case-ctrl | With vs without impairment | 13 | 568 | 1809 | SMD | 0.6 (0.4, 0.8) | | 3.21 (2.20, 4.67) | | 1.18E-09 | 0.9, 11.3 | 66.5 | 0.017 | <.01 | IV | No |
| Loures, 2019 [28] | Mild cognitive impairment | General | Case-ctrl | Patients vs ctrls | 3 | 158 | 400 | SMD | 0.4 (0.2, 0.6) | | 2.21 (1.51, 3.24) | | 4.3E-05 | 0.2, 26.1 | 0 | 0.85 | 0.40 | IV | No |
| Nie, 2014 [29] | Cognitive decline | General | Prospective |  | 15 | 1543 | 16018 | RR | 1.5 (1.2, 1.8) | | 1.50 (1.23, 1.84) | | 6.3E-05 | 0.8, 2.9 | 71.8 | 0.00015 | <.01 | III | No |
| Zhou, 2019 [24] | Cognitive impairment without dementia | General | Prospective | Highest vs lowest | 8 | 542 | 4783 | RR | 1.3 (1.0, 1.8) | | 1.35 (1.02, 1.77) | | 0.0346 | 0.6, 2.9 | 54.6 | 0.094 | 0.44 | IV | Yes |
| Kim, 2022 [30] | Post-stroke cognitive impairment | Post-stroke patients | Case-ctrl | Patients vs ctrls | 6 | 1934 | 3053 | SMD | 0.4 (-0.03, 0.8) | | 2.09 (0.95, 4.61) | | 0.0685 | 0.1, 36 | 82.1 | 0.099 | <.01 | NL | Yes |
| Huang, 2015 [31] | Age-related macular degeneration | General | Case-ctrl | Patients vs ctrls | 15 | 1072 | 2440 | SMD | 0.7 (0.3, 1.1) | | 3.76 (1.82, 7.78) | | 3.5E-04 | 0.2, 79.3 | 94.1 | 0.16 | 0.23 | III | Yes |
| Hu, 2023 [32] | Amyotrophic lateral sclerosis | General | Case-ctrl | Patients vs ctrls | 11 | 812 | 3444 | SMD | 0.5 (-0.05, 1.1) | | 2.47 (0.91, 6.74) | | 0.0765 | 0.05, 122.1 | 95.6 | 0.22 | 0.079 | NL | Yes |
| Stein, 2021 [33] | Peripheral neuropathy | General | Cohort, case–ctrl, and cross-sectional | Patients vs ctrls | 15 | 1047 | 5810 | OR | 3.5 (2, 6.3) | | 3.53 (1.99, 6.25) | | 1.5E-05 | 0.5, 25.9 | 70.6 | 0.28 | 0.16 | III | Yes |
| Chen, 2022 [34] | Poststroke depression | Post-stroke patients | Case-ctrl | Patients vs ctrls | 11 | 842 | 2789 | SMD | 0.4 (0.1, 0.7) | | 1.93 (1.12, 3.33) | | 0.018 | 0.2, 15.5 | 91.1 | 0.26 | <.01 | IV | Yes |
| Jaiswal, 2024 [35] | Post-stroke depression | Post-stroke patients | Case-ctrl | Patients vs ctrls | 9 | 771 | 2435 | SMD | 0.1 (0.05, 0.2) | | 1.30 (1.09, 1.54) | | 0.0029 | 0.96, 1.7 | 0 | 0.16 | 0.41 | IV | No |
| Moradi, 2021 [36] | Depression | General | Cohort, case–ctrl, and cross-sectional | Patients vs ctrls | 26 | 4969 | 25911 | OR | 1.4 (1.1, 1.7) | | 1.36 (1.12, 1.66) | | 1.8E-03 | 0.6, 3.2 | 75.2 | 0.026 | <.01 | IV | Yes |
| Li, X, 2020 [37] | Multiple sclerosis | General | Case-ctrl | Patients vs ctrls | 17 | 1419 | 2624 | SMD | 0.6 (0.3, 0.9) | | 3.01 (1.69, 5.37) | | 1.9E-04 | 0.2, 38 | 93.2 | 0.00093 | <.01 | III | Yes |
| Zhang, 2018 [38] | Systemic sclerosis | General | Case-ctrl | Patients vs ctrls | 6 | 322 | 482 | SMD | 1.2 (0.3, 2.0) | | 8.27 (1.79, 38.16) | | 0.00674 | 0.03, 2085.2 | 94.3 | 0.31 | <.01 | IV | Yes |
| Cong, 2021 [39] | Multiple system atrophy | General | Case-ctrl | Patients vs ctrls | 3 | 155 | 343 | SMD | 0.9 (0.7, 1.1) | | 5.01 (3.34, 7.51) | | 6.96E-15 | 0.4, 69.4 | 0 | 0.98 | 0.46 | IV | Yes |
| Nishi, 2014 [40] | Schizophrenia | Males | Case-ctrl | Patients vs ctrls | 12 | 1079 | 2638 | SMD | 0.7 (0.3, 1.2) | | 3.79 (1.71, 8.38) | | 0.00102 | 0.2, 81.6 | 91.2 | 0.32 | 0.4 | IV | No |
| Nishi, 2014 [40] | Schizophrenia | Females | Case-ctrl | Patients vs ctrls | 10 | 615 | 2076 | SMD | 0.5 (0.3, 0.7) | | 2.47 (1.73, 3.54) | | 7.6E-07 | 0.8, 7.4 | 69.7 | 0.35 | 0.88 | IV | No |
| Nishi, 2014 [40] | Schizophrenia | General | Case-ctrl | Patients vs ctrls | 22 | 1694 | 4714 | SMD | 0.6 (0.4, 0.8) | | 2.98 (1.94, 4.57) | | 5.5E-07 | 0.4, 21.6 | 86.5 | 0.52 | 0.635 | II | Yes |
| Guo, 2020 [41] | Autism spectrum disorder | Children | Case-ctrl | Patients vs ctrls | 36 | 1721 | 3346 | SMD | 0.7 (0.3, 1.1) | | 3.56 (1.76, 7.22) | | 4.2E-04 | 0.05, 272.5 | 91.6 | 0.76 | 0.35 | III | Yes |
| Fraguas, 2019 [42] | First-Episode Psychosis | General | Case-ctrl | Patients vs ctrls | 4 | 149 | 294 | SMD | 0.8 (0.4, 1.3) | | 4.6 (1.90, 11.154 ) | | 0.000732 | 0.1, 191.9 | 66.7 | 0.15 | <.01 | IV | Yes |
| Yan, 2022 [43] | Obsessive-compulsive disorder | General | Case-ctrl | Patients vs ctrls | 3 | 110 | 185 | SMD | 1.1 (0.4, 1.8) | | 7.31 (2.23, 23.95) | | 0.00102 | 0, 6161358.8 | 68.8 | 0.44 | 0.13 | IV | Yes |
| Liampas, 2020 [44] | Migraine | Adults | Case-ctrl | Patients vs ctrls | 12 | 1074 | 1961 | SMD | 0.4 (0.1, 0.6) | | 1.89 (1.24, 2.89) | | 0.0031 | 0.4, 9.1 | 76.5 | 0.65 | 0.3 | IV | Yes |
| Liampas, 2020 [44] | Migraine | Migraine in adults | Case-ctrl | With vs without aura | 6 | 455 | 928 | SMD | 0.2 (0.1, 0.4) | | 1.49 (1.14, 1.94) | | 0.00376 | 0.9, 2.5 | 28.8 | 0.87 | 0.39 | IV | No |
| Li, K, 2017 [45] | Obstructive sleep apnea | General | Case-ctrl | Patients vs ctrls | 14 | 457 | 839 | SMD | 0.5 (-0.2, 1.3) | | 2.60 (0.68, 10) | | 0.165 | 0.01, 734.8 | 92.2 | 0.93 | 0.024 | NL | Yes |
| **Obesity and metabolic disorders** | | | |  |  |  |  |  |  |  |  |  |  |  |  |  |  |  |  |
| Fu, 2019 [46] | Obesity | General | Case-ctrl | Patients vs ctrls | 19 | 2401 | 8230 | SMD | 0.7 (0.3, 1.2) | | 3.77 (1.67, 8.51) | | 0.0014 | 0.1, 168.3 | 95.4 | 0.0023 | <.01 | IV | No |
| Wang, 2021 [47] | Obesity (case-ctrl) | General | Case-ctrl | Patients vs ctrls | 4 | 233 | 416 | SMD | 1.8 (-0.1, 3.7) | | 25.29 (0.83, 772.93) | | 0.0641 | 0, 400031327.9 | 96.2 | 0.89 | 0.52 | NL | Yes |
| Wang, 2021 [47] | Obesity (cross-sectional) | General | Cross-sectional | Patients vs ctrls | 11 | 447 | 841 | SMD | 0.3 (0.1, 0.6) | | 1.83 (1.10, 3.03) | | 0.0196 | 0.3, 10.2 | 68.8 | 0.72 | 0.75 | IV | No |
| Wang, 2021 [48] | Type 2 diabetes (cross-sectional) | General | Cross-sectional | Patients vs ctrls | 18 | 1334 | 3620 | SMD | 0.6 (0.4, 0.8) | | 3.01 (2.07, 4.38) | | 8.46E-09 | 0.6, 14.3 | 80.3 | 0.53 | 0.09 | II | No |
| Wang, 2021 [48] | Type 2 diabetes | General | Case-ctrl | Patients vs ctrls | 5 | 443 | 935 | SMD | 1.0 (-0.3, 2.4) | | 6.58 (0.56, 77.75) | | 0.135 | 0, 112650.7 | 96.1 | 0.33 | 0.56 | NL | Yes |
| Wang, 2021 [48] | Retinopathy in type 2 DM | Type 2 diabetes | Case-ctrl | With vs without retinopathy | 4 | 252 | 465 | SMD | 0.9 (0.2, 1.7) | | 5.52 (1.52, 20) | | 0.0093 | 0.01, 2147.1 | 82.2 | 0.68 | 0.45 | IV | No |
| Wang, 2021 [48] | Nephropathy in type 2 DM | Type 2 diabetes | Cross-sectional | With vs without nephropathy | 9 | 326 | 905 | SMD | 1.1 (0.6, 1.6) | | 7.66 (3.17, 18.56) | | 6.4E-06 | 0.3, 175.3 | 80.1 | 0.29 | 0.46 | IV | Yes |
| Zheng, 2015 [49] | Diabetic peripheral neuropathy | DM | Case-ctrl | With vs without neuropathy | 6 | 603 | 1290 | SMD | 1.3 (0.2, 2.4) | | 11.1 (1.57, 78.6) | | 0.016 | 0.01, 15724.6 | 98.4 | 0.32 | 0.28 | IV | Yes |
| Guo, 2016 [50] | Neuropathy in type 2 diabetes | Type 2 diabetes | Case-ctrl | With vs without neuropathy | 5 | 439 | 784 | SMD | 1.0 (0.5, 1.6) | | 6.52 (2.42, 17.59) | | 2.2E-04 | 0.2, 280.3 | 91.8 | 0.82 | 0.22 | IV | Yes |
| Feng, 2015 [51] | Type 1 DM without any complications | General | Case-ctrl | Patients vs ctrls | 13 | 486 | 987 | SMD | -0.1 (-0.5, 0.3) | | 0.85 (0.41, 1.76) | | 0.656 | 0.05, 14.5 | 81.5 | 0.058 | 0.011 | NL | Yes |
| Feng, 2015 [51] | T1 diabetes nephrenopathy | T1DM | Case-ctrl | Only diabetes nephrenopathy vs without any complications | 4 | 54 | 118 | SMD | 0.7 (0.2, 1.2) | | 3.57 (1.38, 9.22) | | 0.00866 | 0.1, 108.4 | 44.1 | 0.44 | 0.83 | IV | No |
| Feng, 2015 [51] | T1DM with only diabetes retinopathy | T1DM | Case-ctrl | Only diabetes retinopathy vs without any complications | 5 | 148 | 427 | SMD | 0.3 (0.1, 0.6) | | 1.84 (1.23, 2.77) | | 0.0032 | 0.8, 4.0 | 0 | 0.76 | 0.29 | IV | No |
| Zheng, 2021 [52] | Gestational DM | Pregnant women | Case-ctrl | GDM vs healthy pregnant women | 12 | 712 | 1989 | SMD | 0.6 (0.2, 0.9) | | 2.74 (1.48, 5.06) | | 0.00134 | 0.3, 27.7 | 84 | 0.38 | 0.33 | IV | Yes |
| Lei, 2018 [53] | Diabetic retinopathy | General | Cohort | High vs low | 11 | 781 | 2039 | OR | 1.6 (1.3, 2.1) | | 1.63 (1.28, 2.09) | | 8.2E-05 | 0.8, 3.3 | 58.3 | 0.051 | <.01 | IV | Yes |
| Ulloque-Badaracco, 2023 [54] | Metabolic syndrome | General | Cohort, case–ctrl, and cross-sectional | Patients vs ctrls | 60 | 6000 | 18000 | OR | 1.5 (1.3, 1.8) | | 1.52 (1.3, 1.78) | | 2.9E-07 | 0.5, 4.8 | 90.4 | 0.0001 | <.01 | III | Yes |
| Merashli, 2022 [55] | Ocular involvement in Behcet's syndrome | Patients with Behcet's syndrome | Case-ctrl | With vs without ocular involvement | 7 | 285 | 551 | SMD | 1.3 (0.5, 2.1) | | 10.09 (2.45, 41.58) | | 0.00138 | 0.1, 1600.6 | 91.9 | 0.067 | 0.53 | IV | No |
| Merashli, 2022 [55] | Overall vascular involvement in Behcet's syndrome | Patients with Behcet's syndrome | Case-ctrl | With vs without overall vascular involvement | 18 | 1018 | 2150 | SMD | 0.6 (0.3, 0.9) | | 3.06 (1.85, 5.06) | | 1.4E-05 | 0.3, 27.8 | 84.1 | 0.22 | 0.094 | III | No |
| Merashli, 2022 [55] | Venous thrombosis only in Behcet's syndrome | Patients with Behcet's syndrome | Case-ctrl | With vs without venous thrombosis | 11 | 666 | 1243 | SMD | 0.5 (0.3, 0.7) | | 2.47 (1.79, 3.42) | | 4.68E-08 | 0.99, 6.2 | 48.2 | 0.72 | 0.38 | IV | No |
| Merashli, 2022 [55] | Active Behcet's syndrome | Patients with Behcet's syndrome | Case-ctrl | Inactive vs active | 10 | 470 | 756 | SMD | 1.0 (0.7, 1.4) | | 6.44 (3.48, 11.91) | | 2.91E-09 | 0.8, 54.3 | 75.8 | 0.48 | 0.376 | IV | No |
| La, 2010 [56] | Thrombosis in vascular Behcet disease | Patients with vascular Behcet disease |  | With vs without thrombosis in vascular Behcet disease | 8 | 154 | 481 | OR | 3.1 (1.3, 7.5) | | 3.1 (1.28, 7.53) | | 0.0124 | 0.2, 43.0 | 61.6 | 0.72 | 0.52 | IV | No |
| Merashli, 2022 [55] | Behcet's syndrome | General | Case-ctrl | Patients vs ctrls | 48 | 2501 | 4628 | SMD | 0.8 (0.6, 1) | | 3.97 (2.72, 5.8) | | 9.52E-13 | 0.3, 51.6 | 88.8 | 0.037 | 0.35 | II | Yes |
| Merashli, 2022 [57] | Buerger's disease | General | Cohort and/or case–ctrl | Patients vs ctrls | 7 | 193 | 596 | SMD | 0.8 (0.6, 1.0) | | 4.52 (3.07, 6.66) | | 1.91E-14 | 2.3, 9.0 | 8.9 | 0.97 | 0.7 | IV | Yes |
| Zhang S, 2020 [58] | Subclinical hypothyroidism | General | Case-ctrl | Patients vs ctrls | 12 | 684 | 1313 | SMD | 0.3 (0.1, 0.5) | | 1.88 (1.31, 2.71) | | 6.7E-04 | 0.6, 6.3 | 64 | 0.86 | 0.35 | IV | Yes |
| **Cancer and cause-specific mortality** | | | |  |  |  |  |  |  |  |  |  |  |  |  |  |  |  |  |
| Fan, 2017 [59] | All-cause mortality | General | Prospective | Highest vs lowest | 11 | 4110 | 31847 | RR | 1.8 (1.5, 2.1) | | 1.79 (1.51, 2.12) | | 1.32E-11 | 1.1, 3 | 64.7 | 0.051 | <.01 | II | Yes |
| Peng, 2015 [60] | All-cause mortality | General | Prospective | Highest vs lowest | 7 | 700 | 2100 | RR | 1.9 (1.5, 2.4) | | 1.94 (1.53, 2.44) | | 3.08E-08 | 1.0, 3.7 | 52.8 | 0.93 | 0.17 | IV | No |
| Peng, 2015 [60] | All-cause mortality (per 5 unit) | General | Prospective | 5-unit increase | 3 | 300 | 900 | RR | 1.3 (1.1, 1.5) | | 1.26 (1.06, 1.51) | | 0.0108 | 0.1, 10.8 | 86.5 | 0.37 | <.01 | IV | No |
| Zhang, H, 2021 [4] | All-cause mortality | Patients with acute ischemic stroke | Prospective | Highest vs lowest | 6 | 1176 | 18707 | RR | 1.4 (1.2, 1.6) | | 1.42 (1.25, 1.61) | | 6.29E-08 | 1.1, 1.9 | 10.8 | 0.09 | <.01 | II | No |
| Zhang, D, 2015 [61] | All cancer | General | Case-ctrl | Patients vs ctrls | 128 | 14834 | 34974 | SMD | 2.0 (1.5, 2.5) | | 39.8 (15.6, 101.3) | | 1.19E-14 | 0.001, 1705968.8 | 98.2 | 2.39E-21 | <.01 | III | Yes |
| Yang, 2018 [62] | Lung cancer | General | Case-ctrl | Patients vs ctrls | 8 | 1322 | 3649 | SMD | 0.4 (0.2, 0.6) | | 2.08 (1.53, 2.82) | | 3.1E-06 | 0.9, 4.7 | 60.2 | 0.0016 | <.01 | III | Yes |
| Xu, J, 2018 [63] | Digestive tract cancer | General | Case-ctrl, nested or not | Highest vs lowest | 16 | 3609 | 11155 | OR | 1.3 (1.2, 1.4) | | 1.27 (1.16, 1.4) | | 6.8E-07 | 1.1, 1.4 | 0 | 0.58 | 0.5 | I | Yes |
| Xu, J, 2018 [63] | Colorectal cancer | General | Case-ctrl, nested or not | Highest vs lowest | 8 | 2345 | 5905 | OR | 1.3 (1.1, 1.4) | | 1.27 (1.14, 1.41) | | 1E-05 | 1.1, 1.4 | 0 | 0.75 | 0.51 | III | Yes |
| Xu, J, 2018 [63] | Gastric cancer | General | Case-ctrl, nested or not | Highest vs lowest | 4 | 878 | 2859 | OR | 1.2 (0.9, 1.7) | | 1.23 (0.87, 1.74) | | 0.244 | 0.3, 4.4 | 43.8 | 0.30 | 0.44 | NL | Yes |
| Xu, J, 2018 [63] | Esophagogastric cancer | General | Case-ctrl, nested or not | Highest vs lowest | 8 | 1264 | 5250 | OR | 1.3 (1, 1.6) | | 1.26 (0.99, 1.61) | | 0.0556 | 0.8, 2.0 | 0 | 0.31 | 0.569 | NL | Yes |
| Peng, 2015 [60] | Coronary heart disease mortality | General | Prospective | 5-unit increase | 3 | 300 | 900 | RR | 1.5 (1.3, 1.8) | | 1.52 (1.26, 1.84) | | 1.4E-05 | 0.4, 5.2 | 0 | 0.38 | 0.0063 | IV | No |
| Peng, 2015 [60] | Coronary heart disease mortality | General | Prospective | Highest vs lowest | 4 | 400 | 1200 | RR | 1.7 (1.1, 2.5) | | 1.66 (1.12, 2.47) | | 0.012 | 0.7, 4 | 0 | 0.55 | 0.48 | IV | Yes |
| Peng, 2015 [60] | Cardiovascular mortality | General | Prospective | Highest vs lowest | 4 | 400 | 1200 | RR | 1.7 (1.1, 2.6) | | 1.65 (1.07, 2.56) | | 0.0243 | 0.2, 11 | 86 | 0.039 | <.01 | IV | Yes |
| Peng, 2015 [60] | Cardiovascular mortality | General | Prospective | 5-unit increase | 3 | 300 | 900 | RR | 1.3 (1.1, 1.6) | | 1.32 (1.09, 1.60) | | 0.0053 | 0.2, 10.5 | 58.6 | 0.45 | <.01 | IV | No |
| Heinz, 2009 [64] | Mortality of dialysis | Dialysis patients | Prospective | 5 μM increase | 4 | 322 | 814 | HR | 1.07 (1.01, 1.14) | | 1.07 (1.01, 1.14) | | 0.0282 | 0.9, 1.3 | 17.1 | 0.86 | 0.063 | IV | Yes |
| **Digestive diseases** | | | |  |  |  |  |  |  |  |  |  |  |  |  |  |  |  |  |
| Zhong, 2019 [65] | Ulcerative colitis | General | Case-ctrl | Patients vs ctrls | 18 | 1577 | 3170 | SMD | 1.2 (0.8, 1.6) | | 8.97 (4.55, 17.68) | | 2.33E-10 | 0.4, 198.5 | 94.1 | 0.014 | 0.049 | II | Yes |
| Oussalah, 2011 [66] | Crohn’s disease | General | Case-ctrl | Patients vs ctrls | 5 | 279 | 576 | SMD | 1.0 (0.7, 1.2) | | 5.61 (3.32, 9.47) | | 1.15E-10 | 1.1, 29.9 | 60.6 | 0.99 | 0.28 | IV | Yes |
| Oussalah, 2011 [66] | Inflammatory bowel diseases | General | Case-ctrl | High vs low | 11 | 909 | 2256 | OR | 4.7 (3.0, 7.2) | | 4.66 (3.03, 7.19) | | 2.91E-12 | 1.3, 16.6 | 51 | 0.094 | 0.0069 | IV | Yes |
| Yuan, 2022 [67] | Nonalcoholic fatty liver disease | General | Case-ctrl | High vs low | 20 | 11425 | 30143 | OR | 2.0 (1.5, 2.6) | | 2.00 (1.54, 2.6) | | 1.8E-07 | 0.7, 6 | 88.8 | 0.0000098 | <.01 | III | Yes |
| Qi, 2014 [68] | Budd-Chiari syndrome | General | Case-ctrl | Patients vs ctrls | 4 | 151 | 408 | SMD | 0.6 (0.3, 0.8) | | 2.81 (1.88, 4.19) | | 4.5E-07 | 1.1, 7.5 | 23.8 | 0.78 | 0.49 | IV | Yes |
| Qi, 2014 [68] | Budd-Chiari syndrome | General | Case-ctrl | High vs low | 2 | 59 | 223 | OR | 2.6 (1.2, 5.5) | | 2.55 (1.18, 5.50) | | 0.0168 | - | 0 |  |  | IV | No |
| **Sense organ-related diseases** | | |  |  |  |  |  |  |  |  |  |  |  |  |  |  |  |  |  |
| Xu, 2012 [69] | Primary open-angle glaucoma | General | Case-ctrl | Patients vs ctrls | 12 | 546 | 1081 | SMD | 0.9 (0.2, 1.6) | | 4.75 (1.34, 16.84) | | 0.0159 | 0.03, 762.4 | 96.7 | 0.60 | 0.99 | IV | Yes |
| Xu, F, 2012 [70] | Pseudoexfoliation glaucoma | General | Case-ctrl | Patients vs ctrls | 14 | 485 | 941 | SMD | 0.8 (0.5, 1.1) | | 4.46 (2.58, 7.71) | | 8.26E-08 | 0.6, 35.5 | 77.4 | 0.088 | 0.42 | IV | Yes |
| Li, 2016 [71] | Normal-tension glaucoma | General | Case-ctrl | Patients vs ctrls | 4 | 149 | 297 | SMD | 0.4 (-0.01, 0.8) | | 1.96 (0.98, 3.92) | | 0.0564 | 0.1, 33.4 | 63.5 | 0.90 | 0.37 | NL | Yes |
| Niu, 2023 [72] | Sudden sensorineural hearing loss | General | Case-ctrl | High vs low | 3 | 207 | 927 | OR | 3.3 (2.2, 5) | | 3.28 (2.17, 4.95) | | 1.9E-08 | 0.2, 47.9 | 0 | 0.84 | 0.26 | IV | Yes |
| Niu, 2023 [72] | Sudden sensorineural hearing loss | General | Case-ctrl | Patients vs ctrls | 6 | 358 | 766 | SMD | 0.4 (0.1, 0.7) | | 2.12 (1.22, 3.68) | | 0.0078 | 0.3, 12.9 | 73.9 | 0.56 | <.01 | IV | No |
| Li, D, 2014 [73] | Retinal vein occlusion | General | Case-ctrl | Patients vs ctrls | 35 | 2304 | 5033 | SMD | 2.1 (1.2, 2.9) | | 42.48 (9.62, 187.7) | | 7.6E-07 | 0.01, 211959.2 | 89.8 | 0.84 | 0.73 | III | Yes |
| Huang, 2017 [74] | Retinal artery occlusive disease | General | Case-ctrl | High vs low | 6 | 307 | 849 | OR | 6.5 (3.5, 12.1) | | 6.53 (3.53, 12.08) | | 2.32E-09 | 1.2, 36.5 | 56.9 | 0.16 | 0.04 | IV | Yes |
| **Reproductive and congenital diseases** | | | |  |  |  |  |  |  |  |  |  |  |  |  |  |  |  |  |
| Murri, 2013 [75] | Polycystic ovary syndrome | Women | Case-ctrl | Patients vs ctrls | 35 | 2090 | 3511 | SMD | 0.6 (0.4, 0.8) | | 2.98 (2.05, 4.31) | | 8.56E-09 | 0.4, 24.5 | 83.9 | 0.092 | 0.48 | II | Yes |
| Meng, 2016 [76] | Insulin resistance | Women with polycystic ovary syndrome | Case-ctrl | With vs without insulin resistance | 3 | 91 | 175 | SMD | 0.7 (0.4, 1.0) | | 3.48 (1.94, 6.25) | | 2.9E-05 | 0.1, 154.2 | 0 | 0.21 | 0.03 | IV | No |
| Zhang, C, 2022 [77] | Pre-eclampsia | Pregnant woman | Case-ctrl | High vs low | 9 | 4384 | 30405 | OR | 1.7 (1.1, 2.7) | | 1.75 (1.13, 2.71) | | 0.0123 | 0.4, 8.3 | 85.9 | 0.019 | 0.017 | IV | Yes |
| Bala, 2021 [78] | Early pregnancy loss | Women | Case-ctrl | Patients vs ctrls | 3 | 113 | 230 | SMD | 0.3 (0.1, 0.6) | | 1.84 (1.14, 2.97) | | 0.0125 | 0.1, 41.0 | 0 | 0.95 | 0.45 | IV | Yes |
| Diao, 2020 [79] | Recurrent spontaneous abortion | Pregnant woman | Case-ctrl | Patients vs ctrls | 21 | 1870 | 3267 | SMD | 1.5 (0.8, 2.2) | | 14.91 (4.33, 51.32) | | 1.8E-05 | 0.03, 6771.9 | 95.8 | 0.00098 | <.01 | III | Yes |
| Yadav, 2021 [80] | Neural tube defects-affected | Mothers | Case-ctrl | Patients vs ctrls | 34 | 2140 | 5649 | SMD | 0.7 (0.1, 1.3) | | 3.48 (1.15, 10.54) | | 0.027 | 0.004, 2918. | 88.8 | 0.015 | 0.69 | IV | Yes |
| Sansone, 2018 [81] | Erectile dysfunction | Men | Case-ctrl | Patients vs ctrls | 9 | 489 | 1320 | SMD | 1.0 (0.6, 1.3) | | 6.02 (3.19, 11.34) | | 2.92E-08 | 0.7, 54.3 | 85.3 | 0.31 | 0.63 | IV | Yes |
| Blanco, 2016 [82] | Non-syndromic orofacial cleft | Mothers | Case-ctrl | Patients vs ctrls | 10 | 856 | 3216 | SMD | 0.4 (0.003, 0.8) | | 1.99 (1.01, 3.95) | | 0.0483 | 0.2, 26.2 | 92.6 | 0.35 | 0.77 | IV | Yes |
| Verkleij-Hagoort, 2007 [83] | Congenital heart defects | Children with cleft lip | Case-ctrl | High vs low | 3 | 400 | 718 | OR | 4.4 (2.5, 7.6) | | 4.38 (2.54, 7.56) | | 1.1E-07 | 0.04, 460.2 | 14.2 | 0.92 | 0.27 | IV | Yes |
| **Other outcomes** | | |  |  |  |  |  |  |  |  |  |  |  |  |  |  |  |  |  |
| Yang, 2012 [84] | Fracture | General | Prospective | Highest vs lowest | 12 | 1457 | 14934 | RR | 1.6 (1.3, 1.9) | | 1.58 (1.28, 1.95) | | 1.8E-05 | 0.9, 2.9 | 53.6 | 0.038 | 0.013 | III | Yes |
| Mangoni, 2024 [85] | Rheumatoid arthritis | General | Case-ctrl | Patients vs ctrls | 26 | 1729 | 3120 | SMD | 0.7 (0.5, 1) | | 3.78 (2.52, 5.68) | | 1.26E-10 | 0.5, 28.5 | 83.7 | 0.45 | 0.66 | II | Yes |
| Zhao, 2021 [86] | Postmenopausal osteoporosis | General | Case-ctrl | Patients vs ctrls | 12 | 898 | 3043 | SMD | 0.9 (0.1, 1.6) | | 4.79 (1.26, 18.18) | | 0.0212 | 0.02, 1052.9 | 98.8 | 0.052 | 0.99 | IV | Yes |
| Ames, 2022 [87] | Sickle cell disease | General paediatric | Case-ctrl | Patients vs ctrls | 10 | 479 | 1103 | SMD | 1.2 (0.3, 2.1) | | 8.79 (1.78, 43.5) | | 0.0077 | 0.02, 3842.4 | 95.7 | 0.0088 | 0.42 | IV | Yes |
| Tsai, 2019 [88] | Vitiligo | General | Case-ctrl | Patients vs ctrls | 16 | 969 | 1737 | SMD | 0.5 (0.2, 0.8) | | 2.66 (1.54, 4.62) | | 4.8E-04 | 0.3, 27 | 86.5 | 0.78 | 0.23 | IV | Yes |
| Tsai, 2019 [89] | Psoriasis | General | Case-ctrl | Patients vs ctrls | 18 | 1401 | 2653 | SMD | 0.4 (0.2, 0.6) | | 2.09 (1.41, 3.07) | | 2.1E-04 | 0.4, 10.4 | 76.8 | 0.52 | 0.04 | III | Yes |
| Tsai, 2021 [90] | Systemic lupus erythematosus | General | Case-ctrl | Patients vs ctrls | 40 | 3629 | 6884 | SMD | 1.2 (0.4, 1.9) | | 8.17 (2.15, 31.0) | | 0.002 | 0.001, 53075.2 | 95.8 | 0.0031 | <.01 | IV | Yes |
| Li, 2021 [91] | Ankylosing spondylitis | General | Case-ctrl | Patients vs ctrls | 9 | 778 | 1300 | SMD | 0.5 (-0.2, 1.1) | | 2.3 (0.67, 7.89) | | 0.186 | 0.02, 230.3 | 96.3 | 0.93 | 0.52 | NL | Yes |
| Heinz, 2009 [64] | Cardiovascular events | Dialysis patients | Prospective | 5-unit increase | 7 | 351 | 1275 | HR | 1.1 (1.0, 1.1) | | 1.09 (1.04, 1.14) | | 6.6E-04 | 0.97, 1.2 | 58.4 | 0.019 | <.01 | IV | Yes |
| Heinz, 2009 [64] | Cardiovascular disease | Dialysis patients | Case-ctrl | With or without cardovascular disease | 5 | 166 | 366 | SMD | 0.6 (-0.1, 1.2) | | 2.79 (0.84, 9.32) | | 0.0954 | 0.03, 258 | 87.4 | 0.018 | 0.53 | NL | No |
| Ames, 2022 [87] | Sickle cell disease | Adults | Case-ctrl | Patients vs ctrls | 12 | 981 | 1839 | SMD | 0.1 (-0.9, 1.1) | | 1.15 (0.19, 7.04) | | 0.88 | 0.001, 1807.6 | 97.4 | 0.41 | <.01 | NL | Yes |
| Ames, 2022 [87] | Sickle cell disease | Paediatric in USA | Case-ctrl | Patients vs ctrls | 5 | 341 | 819 | SMD | 0.0 (-0.2, 0.3) | | 1.08 (0.68, 1.72) | | 0.753 | 0.235, 5 | 64.8 | 0.51 | 0.32 | NL | Yes |
| Deminice, 2015 [92] | HIV-infected | General | Case-ctrl | Patients vs ctrls | 12 | 945 | 1649 | SMD | 0.3 (-0.2, 0.8) | | 1.68 (0.71, 4) | | 0.24 | 0.06, 49.5 | 90.1 | 0.64 | 0.22 | NL | Yes |
| Deminice, 2015 [92] | HIV-infected exposed to antiretroviral therapy | HIV patients | Case-ctrl | Exposed vs non-exposed to antiretroviral therapy | 7 | 1066 | 1553 | SMD | 0.5 (0.1, 0.9) | | 2.38 (1.14, 4.94) | | 0.02 | 0.2, 30.9 | 90.1 | 0.77 | 0.95 | IV | Yes |
| Zinellu, 2023 [93] | COPD | General | Case-ctrl | Exposed vs non-exposed to antiretroviral therapy | 11 | 610 | 1078 | SMD | 0.8 (0.5, 1.1) | | 4.06 (2.44, 6.75) | | 7.26E-08 | 0.7, 24. | 79.3 | 0.48 | 0.43 | IV | Yes |
| Chen, 2023 [94] | CKD | General | Cohort, case–ctrl, cross-sectional | HHcy vs normal | 11 | 4271 | 86177 | OR | 2.8 (1.7, 2.8) | | 2.18 (1.71, 2.77) | | 2.3E-10 | 1.1, 4.5 | 57.7 | 0.16 | <.01 | II | No |

SSE, small study effect; ES, effect size; NL, null; eOR, equivalent OR; ESB, excess significance bias; PI, prediction interval; CVD, cardiovascular disease; CHD, coronary heart disease; CKD, chronic kidney disease; SD, standard deviation; NAFLD, Nonalcoholic fatty liver disease; COPD, chronic obstructive pulmonary disease; CVD, cardiovascular disease; MI, myocardial infarction.

**Supplementary Table 3.** A summary of credibility assessment for characteristics and quantitative synthesis of meta-analyses of observational studies reporting associations of Hcy with multiple health outcomes.

| Level of credibility | Criteria | | | | Health outcome |
| --- | --- | --- | --- | --- | --- |
|  | No of cases | P for ES | P for LSS | Specific 4 items |  |
| Convincing (I) | >1000 | <10^-6^ | <0.05 | Met | Digestive tract cancer |
| Highly suggestive (II) | >1000 | <10^-6^ | <0.05 | Not met | Ulcerative colitis, Behcet's syndrome, rheumatoid arthritis, schizophrenia, polycystic ovary syndrome, cerebral small vessel disease, peripheral arterial disease, chronic kidney disease, first-time stroke, all-cause mortality |
| Suggestive (III) | >1000 | <10^-3^ | >0.05 | Not met | Retinal vein occlusion, all cancer, recurrent spontaneous abortion, age-related macular degeneration, autism spectrum disorder in children, peripheral neuropathy, multiple sclerosis, calcific aortic valve disease, psoriasis, lung cancer, nonalcoholic fatty liver disease, Parkinson's disease, fracture, metabolic syndrome, cardiovascular disease, colorectal cancer, coronary heart disease, ischemic heart disease |
| Weak (VI) | <1000 | <0.05 | >0.05 | Not met | Cerebral infarction, diabetic peripheral neuropathy, paediatric sickle cell disease, heart failure, systemic sclerosis, systemic lupus erythematosus, nephropathy in type 2 diabetes, obsessive-compulsive disorder, small-vessel occlusion stroke, retinal artery occlusive disease, neuropathy in type 2 diabetes, erectile dysfunction, Crohn’s disease, multiple system atrophy, postmenopausal osteoporosis, primary open-angle glaucoma, inflammatory bowel diseases, first-episode psychosis, Buerger's disease, pseudoexfoliation glaucoma, congenital heart defects in cleft lip, chronic obstructive pulmonary disease, obesity, neural tube defects-affected in mothers, abdominal aortic aneurysm, sudden sensorineural hearing loss, cerebral venous thrombosis, intracerebral hemorrhage, Budd-Chiari syndrome, atrial fibrillation, gestational diabetes, intracranial atherosclerotic stenosis, vitiligo, non-syndromic orofacial cleft, poststroke depression, migraine, subclinical hypothyroidism, early pregnancy loss, pre-eclampsia, Alzheimer's disease, coronary heart disease mortality, cardiovascular mortality, all-cause dementia, diabetic retinopathy, depression, essential hypertension, cognitive impairment without dementia, cardiovascular events in dialysis, mortality of dialysis, stroke |
| NS (V) |  | >0.05 | >0.05 | Not met | Recurrent stroke, ischemic stroke, post-stroke cognitive impairment, amyotrophic lateral sclerosis, obstructive sleep apnea, type 2 diabetes, type 1 diabetes, gastric cancer, esophagogastric cancer, normal-tension glaucoma, ankylosing spondylitis, adult sickle cell disease, paediatric sickle cell disease, AIDS |

ES, effect size; LSS, the largest size study; NS, not significant; Specific 4 items: I^2^ <50%, neither small study effects nor excess significance bias (both *p* > 0.1), 95% prediction interval excluding null; AIDS, acquired immune deficiency syndrome.

**Supplementary Table 4. Characteristics and statistical power of the primary MR studies reporting causal associations of Hcy with multiple health outcomes.**

|  | **Author/year** | **Outcome or phenotype** | **Outcome popul.** | **No/No of events** | **No. IVs** | **R^2^ (%)** | **Metric** | **Estimate of effect (95% CI)** | **P value** | **Power** | **Level of exposure** | **Comment/selection** |
| --- | --- | --- | --- | --- | --- | --- | --- | --- | --- | --- | --- | --- |
|  | **Cardiovascular outcomes** | | | | | | | | | | | |
| 1 | Larsson, 2019 [95] | Coronary artery disease | Euro | 184305/60801 | 18 | 5.9 | OR | 1.02 (0.95-1.09) | 0.61 | 0.143 | per 1 SD inc | Smaller case population |
| 2 | Yuan, 2021 [96] | Coronary artery disease | General | 724160/139364 | 27 | 6 | OR | 1.05 (0.96-1.15) | 0.264 | 0.98 | per 1 SD inc | √ Larger sample size and number of IVs |
| 3 | Miao, 2021 [97] | Coronary heart disease | Euro | NR/184305 | 9 | NR | OR | 1.015 (0.92-1.11) | 0.752 | NA | Per 1-unit inc | Fewer IVs |
| 4 | van Meurs, 2013 [98] | Coronary artery disease | Euro | 124327/31400 | 13 | 5.9 | OR | 1.01 (0.98-1.04) | 0.49 | 0.041 | NR | Smaller case population |
| 5 | Miao, 2021 [97] | Acute myocardial infarction | Euro | NR/181875 | 9 | NR | OR | 1.04 (0.93-1.14) | 0.499 | NA | Per 1-unit inc |  |
| 6 | Xu, 2021 [99] | Coronary artery disease in DM | Euro | 15666/3968 | 9 | NR | OR | 1.14 (0.82-1.58) | 0.43 | NA | NR |  |
| 7 | Yuan, 2021 [96] | Heart failure | Euro | 1146185/56885 | 27 | 6 | OR | 0.96 (0.88-1.05) | 0.372 | 0.642 | per 1 SD inc |  |
| 8 | Sun, 2021 [100] | Atrial fibrillation | Euro (84.2%) | 588190/65446 | 13 | 2.9 | OR | 0.97 (0.92-1.03) | 0.308 | 0.214 | per 1 SD inc |  |
| 9 | Chen, 2021 [101] | Atrial fibrillation | Euro | 1030836/60620 | 9 | NR | OR | 1.08 (0.99-1.17) | 0.075 | NA | per 1 SD inc |  |
| 10 | Yuan, 2021 [96] | Atrial fibrillation | Euro | 1145375/77945 | 27 | 6 | OR | 0.96 (0.91-1.01) | 0.098 | 0.769 | per 1 SD inc | Larger sample size and number of IVs |
| 11 | Wang X, 2023 [102] | Congestive heart failure | Euro | 456348/897 | 3 | NR | OR | 1.75 (0.67-4.56) | 0.25 | NA | per 1 SD inc |  |
| 12 | Wang X, 2023 [102] | Cardiomyopathy | Euro | 159811/3100 | 12 | NR | OR | 0.81 (0.58-1.11) | 0.189 | NA | per 1 SD inc |  |
| 13 | Wang X, 2023 [102] | Non-ischemic cardiomyopathy | Euro | 1763152/11400 | 12 | NR | OR | 1.06 (0.93-1.22) | 0.379 | NA | per 1 SD inc |  |
| 14 | Yuan, 2021 [96] | Aortic valve stenosis | Euro | 367561/3528 | 14 | 6 | OR | 1.14 (0.86-1.5) | 0.356 | 0.475 | per 1 SD inc |  |
| 15 | Yuan, 2021 [96] | Aortic aneurysm | Euro | 5373323/4180 | 27 | 6 | OR | 1.11 (0.92-1.35) | 0.286 | 0.379 | per 1 SD inc |  |
| 16 | Yuan, 2021 [96] | Stroke | General | 961455/66792 | 41 | 6 | OR | 1.11 (1.03-1.20) | 0.008 | 1 | per 1 SD inc |  |
| 17 | Yuan, 2021 [96] | Subarachnoid hemorrhage | Euro | 243956 /8514 | 26 | 6 | OR | 1.26 (1.05-1.51) | 0.013 | 0.999 | per 1 SD inc |  |
| 18 | Yuan, 2021 [96] | Ischemic stroke | General | 48829/NR | 41 | 6 | OR | 1.11 (1.03-1.21) | 0.011 |  | per 1 SD inc | Smaller case population |
| 19 | Liu, 2021 [103] | Ischemic stroke | Euro | 440328/17265 | 13 | 5.9 | OR | 1.10 (0.980-1.23) | 0.107 | 0.833 |  | √ Larger sample size |
| 20 | Larsson, 2019 [95] | All ischemic stoke | Euro | 438847/34217 | 18 | 5.9 | OR | 1.09 (1.02-1.17) | 0.02 | 0.928 | per 1 SD inc |  |
| 21 | Yuan, 2021 [96] | Intracerebral hemorrhage | Euro | 539266/5951 | 39 | 6 | OR | 1.09 (0.89-1.34) | 0.411 | 0.367 | per 1 SD inc |  |
| 22 | Ma, 2022 [104] | Aneurysmal subarachnoid hemorrhage | Euro | 77074/5140 | 9 | NR | OR | 1.10 (0.88-1.39 | 0.398 | NA | NR |  |
| 23 | Liu, 2021 [103] | Large artery atherosclerosis stroke | Euro | 440328/4373 | 13 | 5.9 | OR | 1.09 (0.88-1.31) | 0.424 | 0.295 | NR |  |
| 24 | Liu, 2021 [103] | Cardioembolism stroke | Euro | 440328/7193 | 13 | 5.9 | OR | 0.92 (0.79-1.08) | 0.308 | 0.399 | NR |  |
| 25 | Liu, 2021 [103] | Small artery occlusion stroke | Euro | 440328/5386 | 13 | 5.9 | OR | 1.33 (1.00-1.76) | 0.048 | 0.999 | NR |  |
| 26 | Larsson, 2019 [95] | Small vessel stroke | Euro | 410016/5386 | 18 | 5.9 | OR | 1.34 (1.13-1.58) | 6.7E-04 | 0.998 | per 1 SD inc |  |
| 27 | Larsson, 2019 [95] | Large artery stroke | Euro | 409003/4373 | 18 | 5.9 | OR | 1.01 (0.84-1.21) | 0.89 | 0.035 | per 1 SD inc |  |
| 28 | Larsson, 2019 [95] | Cardioembolic stroke | Euro | 411823/7193 | 18 | 5.9 | OR | 0.94 (0.81-1.07) | 0.35 | 0.056 | per 1 SD inc |  |
| 29 | Cao, 2021 [105] | Lacunes | Chinese | 1023/139 | 1 | 1 | OR | 2.14 (1.4-3.27) | <0.00001 | 0.13 | NR |  |
| 30 | Wen, 2023 [106] | Intracranial aneurysm | Euro | 79429/7495 | 9 |  | OR | 1.38 (1.07-1.79) | 0.018 | 0.802 | per 1 SD inc |  |
| 31 | Ma, 2022 [104] | Intracranial aneurysm | Euro | 79429/7495 | 9 | 1.12 | OR | 1.12 (0.92-1.37) | 0.246 | 0.173 | NR |  |
| 32 | Yuan, 2021 [96] | Transient ischemic attack | Euro | 538576/11542 | 28 | 6 | OR | 1.15 (0.99-1.33) | 0.066 | 0.953 | per 1 SD inc | Larger number of IVs |
| 33 | Liu, 2021 [103] | Transient ischemic attack | Euro | 461646/1364 | 13 | 5.9 | OR | 1.0 (0.999-1.001) | 0.858 | 0.025 | NR |  |
| 34 | Ma, 2022 [104] | Unruptured intracranial aneurysm | Euro | 74004/2070 | 7 | NR | OR | 1.126 (0.68-1.86) | 0.644 | NA | NR |  |
| 35 | Yuan, 2021 [96] | Venous thromboembolism | Euro | 544460/23325 | 27 | 6 | OR | 1.05 (0.94-1.16) | 0.392 | 0.431 | per 1 SD inc |  |
| 36 | Yuan, 2021 [96] | Peripheral arterial disease | Euro | 540727/9916 | 27 | 6 | OR | 1.06 (0.91-1.23) | 0.486 | 0.291 | per 1 SD inc |  |
| 37 | Fu, 2019 [107] | Hypertension | General | 40173/14378 | 1 | 1 | OR | 1.32 (1.22-1.49) |  | 0.76 | per 5-unit inc |  |
| 38 | Li, 2019 [108] | Hypertension in pregnancy | Chinese | 2188/1077 | 1 | 1 | OR | 3.21 (2.36-4.07) | 7.4E-04 | 0.779 | per 1 SD inc |  |
|  | **Neurocognitive disorders** | | | | | | | | | | | |
| 39 | Larsson, 2017 [109] | Alzheimer' disease | Europea | 54162/17008 | 18 | 5.9 | OR | 0.99 (0.88-1.11) | 0.86 | 0.045 | per 1 SD inc | Smaller case population |
| 40 | Hu, 2016 [110] | Alzheimer' disease | General | 9397/4120 | 1 | 1 | OR | 3.37 (1.90-5.95) | 2.9E-05 | 1 | per 1 SD inc | Fewer IVs |
| 41 | Hu, 2016 [110] | Alzheimer' disease | Caucasian | 4516/2165 | 1 | 1 | OR | 1.67 (0.95-2.95) | 0.75 | 0.987 | per 1 SD inc | Fewer IVs |
| 42 | Hu, 2016 [110] | Alzheimer' disease | Asian | 4056/1560 | 1 | 1 | OR | 6.69 (2.13-20.99) | 0.0011 | 1 | per 1 SD inc | Fewer IVs |
| 43 | Hu, 2016 [110] | Alzheimer' disease | Other | 828/395 | 1 | 1 | OR | 12.6 (3.15-50.61) | 0.0034 | 1 | per 1 SD inc | Fewer IVs |
| 44 | Liu, 2021 [103] | Alzheimer' disease | Euro | 63926/21982 | 13 | 5.9 | OR | 1.08 (0.96-1.22) | 0.198 | 0.623 | NR | √ Larger sample size and number of IVs |
| 45 | Liu, 2021 [103] | Frontotemporal dementia | Euro | 3024/515 | 13 | 5.9 | OR | 1.27 (0.42-3.86) | 0.676 | 0.221 | NR |  |
| 46 | Wu, 2017 [111] | Vascular dementia | General | 1880/722 | 1 | 1 | OR | 4.29 (1.11-16.57) | 0.034 | 0.867 | per 1 SD inc |  |
| 47 | Liu, 2021 [103] | Parkinson's disease | Euro | 482730/33674 | 13 | 5.9 | OR | 0.99 (0.85-1.14) | 0.837 | 0.095 | NR |  |
| 48 | Zhao, 2021 [112] | Parkinson's disease | Euro | 482730/33674 | 14 | NR | OR | 1.01 (0.88-1.16) | 0.868 | NA | per 1 SD inc | Larger sample size and number of IVs |
| 49 | Zhao, 2021 [112] | Age at onset in PD | Euro | 467052/17996 | 14 | NR | beta | -0.65 (-1.7, 0.4) | 0.222 | NA | per 1 SD inc |  |
| 50 | Liu, 2021 [103] | Amyotrophic lateral sclerosis | Euro | 80610/20806 | 13 | 5.9 | OR | 1.09 (0.95-1.24) | 0.235 | 0.692 | NR |  |
| 51 | Peng, 2021 [113] | Multiple sclerosis | Euro | 115803/47429 | 14 | 6 | OR | 0.78 (0.64-0.94) | 0.0106 | 1 | per 1 SD inc | √ Larger sample size and number of IVs |
| 52 | Liu, 2021 [103] | Multiple sclerosis | Euro | 38589/14498 | 13 | 5.9 | OR | 1.15 (0.89-1.50) | 0.283 | 0.912 | NR | Smaller case population |
| 53 | Numata, 2015 [114] | Schizophrenia | General | 25599/11042 | 1 | 1 | OR | 2.15 (1.39-3.32) | 5.3E-04 | 1 | per 1 SD inc | fewer IVs |
| 54 | Yu J, 2022 [115] | Schizophrenia | Euro | 161405/67390 | 10 | 2^#^ | OR | 1.11 (1.03-1.20) | 2.74E-03 | 0.832 | NR | √ Larger sample size and number of IVs |
| 55 | Nishi, 2014 [40] | Schizophrenia | Japan | 10378/4316 | 1 | 1 | OR | 1.14 (1.03-1.27) | 0.016 | 0.096 | per 1-unit inc | Fewer IVs |
| 56 | Yu J, 2022 [115] | Bipolar disorder | Euro | 413466/41917 | 11 |  | OR | 1.08 (1.00-1.17) | 0.054 | NA | NR |  |
| 57 | Yu J, 2022 [115] | BD-I type | Euro | 475038/25060 | 13 | 3^#^ | OR | 1.13 (1.03-1.25) | 9.44E-03 | 0.915 | NR |  |
| 58 | Yu J, 2022 [115] | BD-II type | Euro | 370856/6781 | 13 | NR | OR | 0.98 (0.83-1.15) | 0.773 | NA | NR |  |
| 59 | Jin, 2024 [116] | Autism spectral disorder | Euro | 46351/18382 | 13 | NR | OR | 1.03 (0.92-1.15) | 0.63 | NA | NR |  |
| 60 | Yu J, 2022 [115] | Major depressive disorder | Euro | 42455/16823 | 13 | NR | OR | 0.95 (0.89-1.01) | 0.115 | NA | NR |  |
| 61 | Gao, 2024 [117] | Brain atrophy | Caucasian British | 7916/NR | 9 | NR | OR | 0.96 (0.81-1.14) | NR | NA | NR |  |
|  | **Obesity and metabolic disorders** | | | | | | | | | | | |
| 62 | Kumar, 2015 [118] | T2DM | Euro | 149821/34840 | 5 | NR | OR | 1.09 (0.92-1.30) | 0.34 | NA | per 1 SD inc | Fewer IVs |
| 63 | Cheng, 2022 [119] | T2DM | Euro | 898130/74124 | 14 | 6 | OR | 1.08 (0.95-1.21) | 0.249 | 0.998 | per 1 SD inc | √ Larger sample size and number of IVs |
| 64 | Ma, 2019 [120] | Diabetic kidney disease | Chinese | 1107/547 | 1 | 1 | OR | 3.86 (1.21-2.05) | <0.001 | 0.613 | per 5-unit inc |  |
| 65 | Lee, 2021 [121] | Metabolic syndrome | Korea | 5902/2090 | 5 |  | β | 0.723 (0.50-0.94) | <0.001 | 1 | per 1 SD inc |  |
|  | **Digestive disorders** | | | | | | | | | | | |
| 66 | Fu, 2023 [122] | NAFLD | Euro | 797878/9917 | 9 | 6 | OR | 1.25 (1.05-1.45) | 0.008 | 1 | per 1 SD inc | √ Larger sample size and number of IVs |
| 67 | Yuan, 2022 [67] | NAFLD | Euro | 778614/8434 | 13 | 6 | OR | 1.17 (1.01-1.36) |  | 0.94 | per 1 SD inc | Smaller case population |
| 68 | Chen, 2022 [123] | NAFLD | Euro | 309154/1578 | 12 | 4^#^ | OR | 1.26 (0.92-1.73) | 0.143 | 0.459 | NR | Smaller case population |
| 69 | Chen, 2022 [123] | Non-alcoholic steatohepatitis | Euro | 309154/99 | 12 | 4^#^ | OR | 1.89 (0.51-7.02) | 0.341 | 0.244 | NR |  |
| 70 | Chen, 2022 [123] | NAFLD-related cirrhosis | Euro | 306971/826 | 12 | 4^#^ | OR | 0.81 (0.50-1.32) | 0.401 | 0.224 | NR |  |
|  | **Cancer and cause-specific mortality** | | | | | | | | | | | |
| 71 | Wang, 2020 [124] | Gastric cancer | Chinese Han | 7004/2631 | 15 | 6* | OR | 1.07 (1.01-1.12) | 0.011 | 0.099 | per 1-unit inc | √ Larger number of IVs |
| 72 | Xu, 2016 [125] | Gastric cancer | General | 18206/7566 | 1 | 1 | OR | 2.56 (2.41-2.72) | 5E-04 | 1 | per 1-unit inc | Fewer IVs |
| 73 | He, 2021 [126] | Breast cancer | Euro | 267173/133384 | 15 | NR | OR | 0.97 (0.90-1.06) | 0.543 | NA | NR |  |
| 74 | He, 2021 [126] | Prostate cancer | Euro | 140254/79148 | 15 | NR | OR | 1.01 (0.93-1.11) | 0.774 | NA | NR |  |
| 75 | He, 2021 [126] | Renal cell carcinoma in men | Euro | 8143/3227 | 15 | NR | OR | 0.99 (0.73-1.34) | 0.929 | NA | NR |  |
| 76 | He, 2021 [126] | Renal cell carcinoma in women | Euro | 5087/1992 | 15 | NR | OR | 0.89 (0.61-1.31) | 0.563 | NA | NR |  |
| 77 | Xuan, 2016 [127] | Multiple myeloma | General | 7046/2092 | 1 | 1 | OR | 2.67 (1.12-6.38) | 0.027 | 0.965 | per 1 SD inc |  |
| 78 | Choi, 2023 [128] | All-cause mortality | General | 10005/1691 | 1 | 3.9 | RR | 0.99 (0.62-1.57) |  | 0.03 | per 2-fold inc |  |
| 79 | Choi, 2023 [128] | CVD mortality | General | 10005/240 | 1 | 3.9 | RR | 1.76 (0.54-5.77) |  | 0.401 | per 2-fold inc |  |
|  | **Bone and Joint disorders** | | | | | | | | | | | |
| 80 | Wang P, 2021 [129] | Bone fracture |  | NR/426795 | 5 | 1.78 | OR | 0.97 (0.88-1.07) | 0.562 | NA | per 1-unit inc |  |
| 81 | Hong, 2023 [130] | Overall osteoarthritis | >99% Euro | 826690/177517 | 11 | 1* | OR | 1.10 (1.04-1.16) | 0.001 | 0.937 | NR |  |
| 82 | Hong, 2023 [130] | Knee osteoarthritis | >99% Euro | 396054/62497 | 11 |  | OR | 1.08 (0.99-1.18) | 0.074 | NA | NR |  |
| 83 | Hong, 2023 [130] | Hip osteoarthritis | Euro | 353388/36445 | 11 | 1* | OR | 1.17 (1.03-1.33) | 0.015 | 0.814 | NR |  |
| 84 | Hong, 2023 [130] | Spine osteoarthritis | >98% Euro | 333950/28372 | 11 | 3^#^ | OR | 1.11 (1.02-1.22) | 0.02 | 0.842 | NR |  |
| 85 | Hong, 2023 [130] | Hand osteoarthritis | Euro | 303782/20901 | 11 |  | OR | 1.04 (0.87-1.24) | 0.657 | NA | NR |  |
| 86 | Hong, 2023 [130] | Thumb osteoarthritis | Euro | 247455/10536 | 11 |  | OR | 1.06 (0.86-1.29) | 0.592 | NA | NR |  |
| 87 | Fu, 2022 [131] | Knee osteoarthritis | Euro | 455221/76932 | 14 | 6 | OR | 1.12 (1.03-1.21) | 0.007 | 1 | per 1 SD inc |  |
| 88 | Fu, 2022 [131] | Hospital-diagnosed osteoarthritis | Euro | 327918/30824 | 14 | 6 | OR | 1.18 (1.01-1.37) | 0.034 | 1 | per 1 SD inc |  |
| 89 | Fu, 2022 [131] | Osteoporosis with pathological fracture | Euro | 173619/868 | 13 | 6 | OR | 1.60 (1.04-2.46) | 0.034 | 0.921 | per 1 SD inc |  |
| 90 | Fu, 2022 [131] | Soft tissue disorder | Euro | 218792/115741 | 13 | 6 | OR | 1.07 (1.00-1.14) | 0.045 | 0.968 | per 1 SD inc |  |
| 91 | Wang P, 2021 [129] | Forearm bone mineral density | General | 10805/NA | 8 | 1.78 | beta | -0.111 (0.076) | 0.153 | NA | per 1-unit inc |  |
| 92 | Wang P, 2021 [129] | Femoral neck bone mineral density | General | 49988/NA | 5 | 1.78 | beta | -0.02 (0.058) | 0.731 | NA | per 1-unit inc |  |
| 93 | Wang P, 2021 [129] | Lumbar spine bone mineral density | General | 44731/NA | 5 | 1.78 | beta | -0.001 (0.068) | 0.989 | NA | per 1-unit inc |  |
| 94 | Wang P, 2021 [129] | Estimated heel bone mineral density | General | 426824/NA | 5 | 1.78 | beta | 0.028 (0.0398) | 0.468 | NA | per 1-unit inc |  |
| 95 | Wang, 2023 [132] | Forearm bone mineral density | Euro | 8143/NA | 8 | NR | OR | 0.96 (0.77-1.19) | 0.69 | NA | per 1 SD inc |  |
| 96 | Wang, 2023 [132] | Lumbar bone mineral density | Euro | 28498/NA | 7 | NR | OR | 0.86 (0.72-1.02) | 0.077 | NA | per 1 SD inc |  |
| 97 | Wang, 2023 [132] | Heel bone mineral density | Euro | 142487/NA | 8 | NR | OR | 0.96 (0.93-0.99) | 0.011 | NA | per 1 SD inc |  |
|  | **Others** | | | | | | | | | | | |
| 98 | Hu, 2023 [133] | COPD-related chronic infections | Euro | 186957/234 | 14 | NR | OR | 1.50 (0.57–3.99) | 0.41 | NA | NR |  |
| 99 | Hu, 2023 [133] | COPD/asthma/ILD-related pneumonia or pneumonia-derived septicemia | Euro | 187582/27715 | 14 | NR | OR | 0.93 (0.86–1.02) | 0.13 | NA | NR |  |
| 100 | Hu, 2023 [133] | COPD-related respiratory insufficiency | Euro | 187754/1031 | 14 | NR | OR | 1.00 (0.70–1.44) | 0.99 | NA | NR |  |
| 101 | Hu, 2023 [133] | COPD hospital admissions | Euro | 218792/6500 | 14 | NR | OR | 1.06 (0.91–1.24) | 0.42 | NA | NR |  |
| 102 | Hu, 2023 [133] | Asthma/COPD | Euro | 208167/21444 | 14 | NR | OR | 0.97 (0.89–1.06) | 0.55 | NA | NR |  |
| 103 | Wang L, 2023 [134] | Chronic kidney disease | Euro | 20920/1533 | 14 | 3.22 | OR | 1.32 (1.06-1.63) | 0.012 | 0.467 | per 1 SD inc |  |
| 104 | Xiong, 2022 [135] | Chronic kidney disease | Non-Euro | 480698/41395 |  | NR | OR | 1.24 (1.07-1.44) | <0.05 | NA | per 1 SD inc | √ Larger sample size and number of IVs |
| 105 | Kjaergaard, 2022 [136] | Pregnancy loss | Euro | 194174 | 18 | 5.9 | beta | −0.00 (−0.04, 0.03) |  | NA | per 1 SD inc |  |
| 106 | Chen, 2023 [137] | Psoriasis | Euro | 373338/9267 | 11 |  | OR | 1.00 (0.86-1.15) | 0.941 | NA | NR |  |

*denoting a more conservative value; # denoting an approximate value. MR, Mendelian Randomization; IV, instrumental variable; NR, not report; NA, not applicable; SD, standard deviation; NAFLD, Nonalcoholic fatty liver disease; COPD, chronic obstructive pulmonary disease; CVD, cardiovascular disease; T2DM, type 2 diabetes mellitus; DM, diabetes mellitus; BD, bipolar disorder; inc, increase; Euro, European; PD, Parkinson's disease; OR, odds ratio; ILD, interstitial lung disease;

## **Supplementary Table 5. Methodological quality assessment of included meta-analytic studies using AMSTAR 2.**

| **Study** | **Item 1** | **Item 2** | **Item 3** | **Item 4** | **Item 5** | **Item 6** | **Item 7** | **Item 8** | **Item 9** | **Item 10** | **Item 11** | **Item 12** | **Item 13** | **Item 14** | **Item 15** | **Item 16** | **Overall Rating** |
| --- | --- | --- | --- | --- | --- | --- | --- | --- | --- | --- | --- | --- | --- | --- | --- | --- | --- |
| Ames, 2022 [87] | 2 | 1 | 2 | 2 | 2 | 2 | 0 | 2 | 2 | 2 | 2 | 2 | 2 | 2 | 2 | 2 | Low |
| Bala, 2021 [78] | 2 | 1 | 2 | 2 | 2 | 2 | 0 | 2 | 0 | 0 | 2 | 2 | 0 | 0 | 2 | 2 | Critically low |
| Bautista, 2002 [13] | 2 | 1 | 2 | 0 | 0 | 0 | 0 | 2 | 2 | 0 | 2 | 2 | 0 | 0 | 2 | 0 | Critically low |
| Blanco, 2016 [82] | 2 | 1 | 0 | 2 | 0 | 0 | 0 | 2 | 2 | 0 | 2 | 2 | 2 | 2 | 2 | 2 | Low |
| Cao, 2014 [20] | 2 | 1 | 0 | 2 | 0 | 2 | 0 | 2 | 2 | 0 | 2 | 2 | 2 | 2 | 2 | 2 | Low |
| Chen, 2022 [34] | 2 | 1 | 2 | 2 | 2 | 2 | 0 | 2 | 2 | 0 | 2 | 2 | 2 | 2 | 2 | 0 | Low |
| Chen, 2023 [94] | 2 | 1 | 2 | 2 | 2 | 2 | 0 | 2 | 2 | 0 | 2 | 2 | 2 | 2 | 2 | 2 | Low |
| Clarke, 2002 [2] | 2 | 1 | 2 | 0 | 2 | 2 | 0 | 2 | 2 | 0 | 2 | 2 | 2 | 2 | 2 | 2 | Critically low |
| Cong, 2021 [39] | 2 | 1 | 2 | 2 | 2 | 2 | 0 | 2 | 2 | 0 | 2 | 2 | 2 | 2 | 2 | 2 | Low |
| Diao, 2020 [79] | 2 | 1 | 2 | 2 | 2 | 2 | 0 | 2 | 2 | 0 | 2 | 2 | 2 | 2 | 2 | 2 | Low |
| Fan, 2017 [59] | 2 | 1 | 2 | 2 | 0 | 2 | 0 | 2 | 2 | 0 | 2 | 2 | 2 | 2 | 2 | 0 | Low |
| Fraguas, 2019 [42] | 2 | 1 | 2 | 0 | 0 | 0 | 0 | 2 | 2 | 0 | 2 | 2 | 2 | 2 | 2 | 2 | Critically low |
| Fu, 2015 [6] | 2 | 1 | 2 | 2 | 0 | 0 | 0 | 2 | 2 | 0 | 2 | 2 | 2 | 2 | 2 | 0 | Low |
| Fu, 2019 [46] | 2 | 1 | 0 | 2 | 0 | 2 | 0 | 2 | 0 | 0 | 2 | 0 | 0 | 2 | 2 | 2 | Critically low |
| Guo, 2016 [50] | 2 | 1 | 2 | 2 | 2 | 2 | 0 | 2 | 2 | 0 | 2 | 0 | 2 | 2 | 0 | 2 | Critically low |
| Guo, 2020 [41] | 2 | 1 | 2 | 2 | 0 | 2 | 0 | 2 | 2 | 0 | 2 | 2 | 2 | 2 | 2 | 2 | Low |
| Heinz, 2009 [64] | 2 | 1 | 2 | 2 | 0 | 2 | 0 | 2 | 0 | 0 | 2 | 2 | 2 | 2 | 0 | 2 | Critically low |
| Huang, 2015 [31] | 2 | 1 | 2 | 2 | 2 | 2 | 0 | 2 | 0 | 0 | 2 | 0 | 0 | 2 | 2 | 2 | Critically low |
| Huang, 2017 [74] | 2 | 1 | 2 | 2 | 2 | 2 | 0 | 2 | 2 | 0 | 2 | 2 | 2 | 2 | 2 | 2 | Low |
| Humphrey, 2008 [17] | 2 | 1 | 2 | 2 | 0 | 0 | 0 | 2 | 0 | 0 | 2 | 2 | 2 | 2 | 2 | 0 | Critically low |
| Jin, 2021 [19] | 2 | 1 | 2 | 2 | 2 | 2 | 0 | 2 | 2 | 0 | 2 | 0 | 2 | 2 | 2 | 0 | Low |
| Lauw, 2013 [7] | 2 | 1 | 2 | 2 | 2 | 2 | 0 | 2 | 0 | 0 | 2 | 0 | 2 | 2 | 2 | 0 | Critically low |
| Lei, 2018 [53] | 2 | 1 | 2 | 2 | 0 | 2 | 0 | 2 | 2 | 0 | 2 | 2 | 2 | 2 | 2 | 2 | Low |
| Li H, 2022 [1] | 2 | 1 | 2 | 2 | 0 | 2 | 0 | 2 | 2 | 0 | 2 | 2 | 2 | 2 | 2 | 0 | Low |
| Li, D, 2014 [73] | 2 | 1 | 2 | 2 | 0 | 2 | 0 | 2 | 2 | 0 | 2 | 2 | 2 | 2 | 2 | 0 | Low |
| Li, X, 2020 [37] | 2 | 1 | 2 | 2 | 0 | 0 | 0 | 2 | 2 | 0 | 2 | 2 | 2 | 2 | 2 | 2 | Low |
| Liampas, 2020 [44] | 2 | 1 | 2 | 2 | 2 | 2 | 0 | 2 | 2 | 0 | 2 | 2 | 2 | 2 | 2 | 2 | Low |
| Liu, 2023 [25] | 2 | 2 | 2 | 2 | 0 | 2 | 0 | 2 | 2 | 0 | 2 | 2 | 2 | 2 | 2 | 2 | Low |
| Mangoni, 2024 [85] | 2 | 2 | 2 | 2 | 0 | 0 | 0 | 2 | 2 | 0 | 2 | 2 | 2 | 2 | 2 | 2 | Low |
| Merashli, 2022 [57] | 2 | 2 | 2 | 2 | 2 | 2 | 0 | 2 | 2 | 0 | 2 | 2 | 2 | 2 | 0 | 2 | Critically low |
| Moradi, 2021 [36] | 2 | 1 | 2 | 2 | 0 | 2 | 0 | 2 | 2 | 0 | 2 | 2 | 2 | 2 | 2 | 0 | Low |
| Murri, 2013 [75] | 2 | 1 | 2 | 2 | 0 | 2 | 0 | 2 | 2 | 0 | 2 | 2 | 2 | 2 | 2 | 2 | Low |
| Nishi, 2014 [40] | 0 | 0 | 2 | 0 | 0 | 0 | 0 | 0 | 0 | 0 | 2 | 0 | 0 | 2 | 2 | 2 | Critically low |
| Niu, 2023 [72] | 2 | 1 | 2 | 2 | 0 | 2 | 0 | 2 | 2 | 0 | 2 | 2 | 2 | 2 | 2 | 2 | Low |
| Oussalah, 2011 [66] | 2 | 1 | 2 | 2 | 0 | 2 | 0 | 2 | 0 | 0 | 2 | 2 | 2 | 2 | 2 | 0 | Critically low |
| Peng, 2015 [60] | 2 | 1 | 2 | 2 | 0 | 2 | 0 | 2 | 2 | 0 | 2 | 2 | 2 | 2 | 2 | 0 | Low |
| Piao, 2018 [11] | 2 | 1 | 2 | 2 | 0 | 0 | 0 | 2 | 2 | 0 | 2 | 2 | 2 | 2 | 2 | 0 | Low |
| Qi, 2014 [68] | 2 | 1 | 2 | 2 | 0 | 2 | 0 | 2 | 2 | 0 | 2 | 2 | 2 | 2 | 2 | 2 | Low |
| Rong, 2020 [15] | 2 | 1 | 2 | 2 | 2 | 2 | 0 | 2 | 2 | 0 | 2 | 2 | 2 | 2 | 2 | 0 | Low |
| Sansone, 2018 [81] | 2 | 2 | 2 | 2 | 0 | 2 | 0 | 2 | 2 | 0 | 2 | 2 | 2 | 2 | 2 | 0 | Low |
| Stein, 2021 [33] | 2 | 2 | 2 | 2 | 2 | 2 | 0 | 2 | 2 | 0 | 2 | 2 | 2 | 2 | 2 | 0 | Low |
| Tian, 2023 [12] | 2 | 2 | 2 | 2 | 0 | 2 | 0 | 2 | 2 | 0 | 2 | 2 | 2 | 2 | 2 | 0 | Low |
| Tsai, 2019 [88] | 2 | 2 | 2 | 2 | 0 | 2 | 0 | 2 | 2 | 0 | 2 | 2 | 2 | 2 | 2 | 2 | Low |
| Tsai, 2021 [90] | 2 | 2 | 2 | 2 | 0 | 2 | 0 | 2 | 2 | 0 | 2 | 2 | 2 | 2 | 2 | 2 | Low |
| Ulloque, 2023 [54] | 2 | 2 | 2 | 2 | 2 | 2 | 0 | 2 | 2 | 0 | 2 | 2 | 2 | 2 | 2 | 2 | Low |
| Verkleij, 2007 [83] | 2 | 1 | 2 | 2 | 0 | 0 | 0 | 2 | 0 | 0 | 2 | 0 | 0 | 2 | 2 | 0 | Critically low |
| Wang, 2021 [48] | 2 | 1 | 2 | 2 | 0 | 2 | 0 | 2 | 2 | 0 | 2 | 2 | 2 | 2 | 2 | 2 | Low |
| Wang, H, 2022 [21] | 2 | 2 | 2 | 2 | 0 | 2 | 0 | 2 | 2 | 0 | 2 | 2 | 2 | 2 | 2 | 2 | Low |
| Wu, 2018 [14] | 2 | 1 | 2 | 2 | 0 | 2 | 0 | 2 | 2 | 0 | 2 | 2 | 2 | 2 | 2 | 2 | Low |
| Wu, 2020 [3] | 2 | 1 | 2 | 2 | 0 | 2 | 0 | 2 | 2 | 0 | 2 | 2 | 2 | 2 | 2 | 2 | Low |
| Xu, 2012 [70] | 2 | 1 | 2 | 2 | 0 | 2 | 0 | 2 | 0 | 0 | 2 | 2 | 2 | 2 | 2 | 0 | Critically low |
| Xu, F, 2012 [69] | 2 | 1 | 2 | 2 | 0 | 2 | 0 | 2 | 0 | 0 | 2 | 2 | 2 | 2 | 0 | 0 | Critically low |
| Xu, J, 2018 [63] | 2 | 1 | 2 | 2 | 0 | 2 | 0 | 2 | 2 | 0 | 2 | 2 | 2 | 2 | 2 | 2 | Low |
| Yadav, 2021 [80] | 2 | 1 | 2 | 2 | 0 | 2 | 0 | 2 | 0 | 0 | 2 | 2 | 2 | 2 | 2 | 0 | Critically low |
| Yan, 2022 [43] | 2 | 2 | 2 | 2 | 2 | 2 | 0 | 2 | 2 | 0 | 2 | 2 | 2 | 2 | 2 | 0 | Low |
| Yang, 2012 [84] | 2 | 1 | 2 | 2 | 0 | 2 | 0 | 2 | 2 | 0 | 2 | 2 | 2 | 2 | 2 | 2 | Low |
| Yang, 2018 [62] | 2 | 2 | 2 | 2 | 0 | 2 | 0 | 2 | 2 | 0 | 2 | 2 | 2 | 2 | 2 | 2 | Low |
| Yuan, 2022 [67] | 2 | 2 | 2 | 2 | 2 | 2 | 0 | 2 | 2 | 0 | 2 | 2 | 2 | 2 | 2 | 0 | Low |
| Zhang S, 2020 [58] | 2 | 1 | 2 | 2 | 2 | 2 | 0 | 2 | 2 | 0 | 2 | 2 | 2 | 2 | 2 | 2 | Low |
| Zhang, 2018 [38] | 2 | 1 | 2 | 2 | 0 | 2 | 0 | 2 | 0 | 0 | 2 | 2 | 0 | 0 | 2 | 2 | Critically low |
| Zhang, 2020 [58] | 2 | 1 | 2 | 2 | 0 | 2 | 0 | 2 | 2 | 0 | 2 | 2 | 2 | 2 | 2 | 2 | Low |
| Zhang, C, 2022 [77] | 2 | 1 | 2 | 2 | 0 | 2 | 0 | 2 | 2 | 0 | 2 | 2 | 2 | 2 | 2 | 2 | Low |
| Zhang, D, 2015 [61] | 2 | 1 | 2 | 2 | 0 | 0 | 0 | 2 | 0 | 0 | 2 | 2 | 2 | 2 | 2 | 2 | Low |
| Zhao, 2021 [86] | 2 | 1 | 2 | 2 | 0 | 2 | 0 | 2 | 2 | 0 | 2 | 2 | 2 | 2 | 2 | 2 | Low |
| Zheng, 2015 [49] | 2 | 1 | 2 | 2 | 0 | 2 | 0 | 2 | 0 | 0 | 2 | 2 | 2 | 2 | 2 | 0 | Low |
| Zheng, 2021 [52] | 2 | 1 | 2 | 2 | 2 | 2 | 0 | 2 | 2 | 0 | 2 | 2 | 2 | 2 | 2 | 0 | Low |
| Zhong, 2017 [22] | 2 | 1 | 2 | 2 | 0 | 2 | 0 | 2 | 2 | 0 | 2 | 2 | 2 | 2 | 2 | 2 | Low |
| Zhong, 2019 [65] | 2 | 1 | 2 | 2 | 0 | 2 | 0 | 2 | 2 | 0 | 2 | 2 | 2 | 2 | 2 | 0 | Low |
| Zhou, 2018 [5] | 2 | 1 | 2 | 2 | 0 | 2 | 0 | 2 | 2 | 0 | 2 | 2 | 2 | 2 | 2 | 2 | Low |
| Zhou, 2019 [24] | 2 | 2 | 2 | 2 | 0 | 2 | 2 | 2 | 2 | 0 | 2 | 2 | 2 | 2 | 2 | 2 | Moderate |
| Zinellu, 2023 [93] | 2 | 2 | 2 | 2 | 0 | 2 | 0 | 2 | 2 | 0 | 2 | 2 | 2 | 2 | 2 | 2 | Low |
| Clarke, 2011 [138] | 2 | 1 | 2 | 2 | 0 | 0 | 0 | 2 | 0 | 0 | 2 | 0 | 0 | 2 | 0 | 0 | Critically low |
| Dai, G, 2017 [139] | 2 | 1 | 2 | 2 | 0 | 2 | 0 | 2 | 2 | 0 | 2 | 2 | 2 | 2 | 2 | 2 | Low |
| Fu, 2023 [140] | 2 | 1 | 2 | 2 | 0 | 2 | 0 | 2 | 2 | 0 | 2 | 2 | 2 | 2 | 2 | 2 | Low |
| Garcia, 2018 [141] | 2 | 1 | 2 | 2 | 0 | 0 | 0 | 2 | 2 | 0 | 2 | 2 | 2 | 2 | 2 | 0 | Low |
| Jardine, 2012 [142] | 2 | 1 | 2 | 2 | 2 | 2 | 0 | 2 | 2 | 0 | 2 | 2 | 2 | 2 | 2 | 2 | Low |
| Li, Y, 2016 [143] | 2 | 1 | 2 | 2 | 2 | 2 | 0 | 2 | 2 | 0 | 2 | 2 | 2 | 2 | 2 | 2 | Low |
| Miller, 2010 [144] | 2 | 1 | 2 | 0 | 0 | 2 | 0 | 2 | 0 | 0 | 2 | 2 | 2 | 2 | 2 | 0 | Critically low |
| Nigwekar, 2016 [145] | 2 | 2 | 2 | 2 | 2 | 2 | 2 | 2 | 2 | 0 | 2 | 2 | 2 | 2 | 2 | 2 | High |
| Park, JH, 2016 [146] | 2 | 1 | 2 | 2 | 0 | 2 | 0 | 2 | 0 | 0 | 2 | 0 | 0 | 2 | 0 | 2 | Critically low |
| Qin, 2011 [147] | 0 | 0 | 2 | 0 | 0 | 0 | 0 | 2 | 0 | 0 | 2 | 2 | 0 | 2 | 0 | 0 | Critically low |
| Qin, X, 2013 [148] | 2 | 1 | 2 | 2 | 0 | 2 | 0 | 2 | 2 | 0 | 2 | 2 | 2 | 2 | 2 | 2 | Low |
| Ruan, 2015 [149] | 2 | 1 | 2 | 2 | 0 | 2 | 0 | 2 | 2 | 0 | 2 | 2 | 2 | 2 | 0 | 0 | Critically low |
| Wang, Z, 2022 [150] | 2 | 2 | 2 | 2 | 0 | 2 | 2 | 2 | 2 | 0 | 2 | 2 | 2 | 2 | 2 | 2 | Moderate |
| Wang, L, 2015 [151] | 2 | 1 | 2 | 2 | 2 | 2 | 0 | 2 | 2 | 0 | 2 | 2 | 2 | 2 | 2 | 2 | Low |
| Zhang, C, 2013 [152] | 2 | 2 | 2 | 2 | 2 | 2 | 0 | 2 | 2 | 0 | 2 | 2 | 2 | 2 | 2 | 2 | Low |

**Note** shaded columns = AMSTART 2 critical domains

*1. Did the research questions and inclusion criteria for the review include the components of PICO?

*2. Did the report of the review contain an explicit statement that the review methods were established prior to the conduct of the review and did the report justify any significant deviations from the protocol?

*3. Did the review authors explain their selection of the study designs for inclusion in the review?

*4. Did the review authors use a comprehensive literature search strategy?

*5. Did the review authors perform study selection in duplicate?

*6. Did the review authors perform data extraction in duplicate?

*7. Did the review authors provide a list of excluded studies and justify the exclusions?
*8. Did the review authors describe the included studies in adequate detail?

*9. Did the review authors use a satisfactory technique for assessing the risk of bias in individual studies that were included in the review?

*10. Did the review authors report on the sources of funding for the studies included in the review?

*11. If meta-analysis was performed, did the review authors use appropriate methods for statistical combination of results?

*12. If meta-analysis was performed, did the review authors assess the potential impact of RoB in individual studies on the results of the meta-analysis or other evidence synthesis?

*13. Did the review authors account for RoB in primary studies when interpreting/discussing the results of the review?

*14. Did the review authors provide a satisfactory explanation for, and discussion of, any heterogeneity observed in the results of the review?

*15. If they performed quantitative synthesis did the review authors carry out an adequate investigation of publication bias (small study bias) and discuss its likely impact on the results of the review?

*16. Did the review authors report any potential sources of conflict of interest, including any funding they received for conducting the review?

**Supplementary Figure 1. Consistency between observational meta-analyses and MR studies for the same health outcome. The *P* value is for the test of the interaction between the 2 types of studies.**


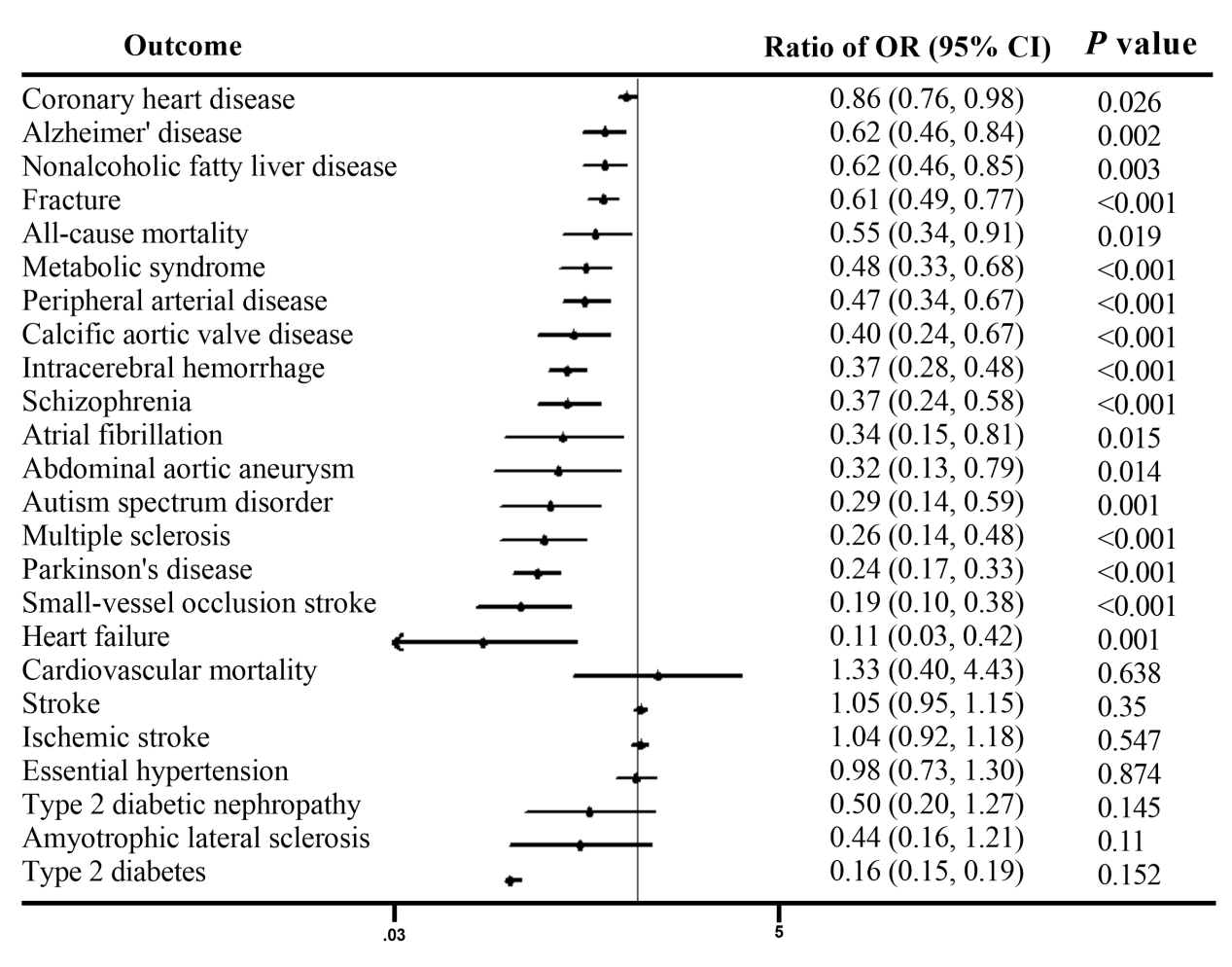


**References**

[1] H. Li, L. Shu, Q. Dai, T. Wu, Association between plasma total homocysteine (thcy) and strokes: a meta-analysis, Pteridines 33 (1) (2022) 58-68, https://doi.org/10.1515/pteridines-2022-0044.

[2] R. Clarke, R. Collins, S. Lewington, A. Donald, S.C. Homocysteine, Homocysteine and risk of ischemic heart disease and stroke: a meta-analysis, JAMA : the journal of the American Medical Association 288 (16) (2002) 2015-2022, https://doi.org/10.1001/jama.288.16.2015.

[3] X. Wu, Q. Zhou, Q. Chen, Q. Li, C. Guo, G. Tian, et al., Association of homocysteine level with risk of stroke: a dose-response meta-analysis of prospective cohort studies, Nutr. Metab. Carbiovasc. Dis. 30 (11) (2020) 1861-1869, https://doi.org/10.1016/j.numecd.2020.07.026.

[4] H. Zhang, J. Huang, Y. Zhou, Y. Fan, Association of homocysteine level with adverse outcomes in patients with acute ischemic stroke: a meta-analysis, Curr. Med. Chem. 28 (36) (2021) 7583-7591, https://doi.org/10.2174/0929867328666210419131016.

[5] Z. Zhou, Y. Liang, H. Qu, M. Zhao, F. Guo, C. Zhao, et al., Plasma homocysteine concentrations and risk of intracerebral hemorrhage: a systematic review and meta-analysis, Sci. Rep. 8 (1) (2018) 2568, https://doi.org/10.1038/s41598-018-21019-3.

[6] H.J. Fu, L.B. Zhao, J.J. Xue, Z.X. Wu, Y.P. Huang, W. Liu, et al., Elevated serum homocysteine (hcy) levels may contribute to the pathogenesis of cerebral infarction, J. Mol. Neurosci. 56 (3) (2015) 553-561, https://doi.org/10.1007/s12031-015-0497-6.

[7] M.N. Lauw, S. Barco, J.M. Coutinho, S. Middeldorp, Cerebral venous thrombosis and thrombophilia: a systematic review and meta-analysis, Semin. Thromb. Hemost. 39 (8) (2013) 913-927, https://doi.org/10.1055/s-0033-1357504.

[8] T. Zhang, Y. Jiang, S. Zhang, T. Tie, Y. Cheng, X. Su, et al., The association between homocysteine and ischemic stroke subtypes in chinese: a meta-analysis, Medicine. (Baltimore). 99 (12) (2020) e19467, https://doi.org/10.1097/MD.0000000000019467.

[9] R.T. Pinzon, V.O. Wijaya, V. Veronica, The role of homocysteine levels as a risk factor of ischemic stroke events: a systematic review and meta-analysis, Front. Neurol. 14 (2023) 1144584, https://doi.org/10.3389/fneur.2023.1144584.

[10] M. Holmen, A.M. Hvas, J. Arendt, Hyperhomocysteinemia and ischemic stroke: a potential dose-response association-a systematic review and meta-analysis, TH Open 5 (3) (2021) e420-e437, https://doi.org/10.1055/s-0041-1735978.

[11] X. Piao, G. Wu, P. Yang, J. Shen, De A, J. Wu, et al., Association between homocysteine and cerebral small vessel disease: a meta-analysis, J. Stroke Cerebrovasc. Dis. 27 (9) (2018) 2423-2430, https://doi.org/10.1016/j.jstrokecerebrovasdis.2018.04.035.

[12] X. Tian, L. Zheng, T.W. Leung, X. Leng, Associations of hematological and biochemical markers with intracranial atherosclerotic stenosis in stroke-free populations: a systematic review and meta-analysis of observational studies, Nutr. Metab. Carbiovasc. Dis. 33 (2) (2023) 287-298, https://doi.org/10.1016/j.numecd.2022.12.007.

[13] L.E. Bautista, I.A. Arenas, A. Peñuela, L.X. Martínez, Total plasma homocysteine level and risk of cardiovascular disease: a meta-analysis of prospective cohort studies, J. Clin. Epidemiol. 55 (9) (2002) 882-887, https://doi.org/10.1016/s0895-4356(02)00434-1.

[14] G. Wu, J. Xian, X. Yang, J. Li, J. Liu, W. Dong, et al., Association between homocysteine levels and calcific aortic valve disease: a systematic review and meta-analysis, Oncotarget 9 (9) (2018) 8665-8674, https://doi.org/10.18632/oncotarget.23938.

[15] H. Rong, L. Huang, N. Jin, J. Hong, J. Hu, S. Wang, et al., Elevated homocysteine levels associated with atrial fibrillation and recurrent atrial fibrillation, Int. Heart J. 61 (4) (2020) 705-712, https://doi.org/10.1536/ihj.20-099.

[16] S.V. Unadkat, B.K. Padhi, A.V. Bhongir, A.P. Gandhi, M.A. Shamim, N. Dahiya, et al., Association between homocysteine and coronary artery disease-trend over time and across the regions: a systematic review and meta-analysis, Egypt. Heart J. 76 (1) (2024) 29, https://doi.org/10.1186/s43044-024-00460-y.

[17] L.L. Humphrey, R. Fu, K. Rogers, M. Freeman, M. Helfand, Homocysteine level and coronary heart disease incidence: a systematic review and meta-analysis, Mayo. Clin. Proc. 83 (11) (2008) 1203-1212, https://doi.org/10.4065/83.11.1203.

[18] B. Wang, X. Mo, Z. Wu, X. Guan, Systematic review and meta-analysis of the correlation between plasma homocysteine levels and coronary heart disease, J. Thorac. Dis. 14 (3) (2022) 646-653, https://doi.org/10.21037/jtd-22-78.

[19] N. Jin, L. Huang, J. Hong, X. Zhao, Y. Chen, J. Hu, et al., Elevated homocysteine levels in patients with heart failure: a systematic review and meta-analysis, Medicine. (Baltimore). 100 (33) (2021) e26875, https://doi.org/10.1097/MD.0000000000026875.

[20] H. Cao, X. Hu, Q. Zhang, J. Li, J. Wang, Y. Shao, et al., Homocysteine level and risk of abdominal aortic aneurysm: a meta-analysis, PLoS One 9 (1) (2014) e85831, https://doi.org/10.1371/journal.pone.0085831.

[21] H. Wang, P. Wu, D. Jiang, H. Zhang, J. Zhang, Y. Zong, et al., Relationship between serum homocysteine, fibrinogen, lipoprotein-a level, and peripheral arterial disease: a dose-response meta-analysis, Eur. J. Med. Res. 27 (1) (2022) 261, https://doi.org/10.1186/s40001-022-00870-1.

[22] F. Zhong, L. Zhuang, Y. Wang, Y. Ma, Homocysteine levels and risk of essential hypertension: a meta-analysis of published epidemiological studies, Clin. Exp. Hypertens. 39 (2) (2017) 160-167, https://doi.org/10.1080/10641963.2016.1226888.

[23] L. Zhang, X. Xie, Y. Sun, F. Zhou, Blood and csf homocysteine levels in alzheimer’s disease: a meta-analysis and meta-regression of case–control studies, Neuropsychiatr. Dis. Treat. 18 (2022) 2391-2403, https://doi.org/10.2147/NDT.S383654.

[24] F. Zhou, S. Chen, Hyperhomocysteinemia and risk of incident cognitive outcomes: an updated dose-response meta-analysis of prospective cohort studies, Ageing Res. Rev. 51 (2019) 55-66, https://doi.org/10.1016/j.arr.2019.02.006.

[25] Y. Liu, M. Gou, X. Guo, Features of plasma homocysteine, vitamin b12, and folate in parkinson's disease: an updated meta-analysis, J. Integr. Neurosci. 22 (5) (2023) 115, https://doi.org/10.31083/j.jin2205115.

[26] B. Dong, R. Wu, Plasma homocysteine, folate and vitamin b12 levels in parkinson's disease in china: a meta-analysis, Clin. Neurol. Neurosurg. 188 (2020) 105587, https://doi.org/10.1016/j.clineuro.2019.105587.

[27] M.T. Periñán, D. Macías-García, S. Jesús, J.F. Martín-Rodríguez, L. Muñoz-Delgado, M.V. Jimenez-Jaraba, et al., Homocysteine levels, genetic background, and cognitive impairment in parkinson's disease, J. Neurol. 270 (1) (2023) 477-485, https://doi.org/10.1007/s00415-022-11361-y.

[28] C. Loures, R. Duarte, M. Silva, W.B. Cicarini, L.C. de Souza, P. Caramelli, et al., Hemostatic abnormalities in dementia: a systematic review and meta-analysis, Semin. Thromb. Hemost. 45 (5) (2019) 514-522, https://doi.org/10.1055/s-0039-1688444.

[29] T. Nie, T. Lu, L. Xie, P. Huang, Y. Lu, M. Jiang, Hyperhomocysteinemia and risk of cognitive decline: a meta-analysis of prospective cohort studies, Eur. Neurol. 72 (3-4) (2014) 241-248, https://doi.org/10.1159/000363054.

[30] K.Y. Kim, K.Y. Shin, K.A. Chang, Potential biomarkers for post-stroke cognitive impairment: a systematic review and meta-analysis, Int. J. Mol. Sci. 23 (2) (2022) https://doi.org/10.3390/ijms23020602.

[31] P. Huang, F. Wang, B.K. Sah, J. Jiang, Z. Ni, J. Wang, et al., Homocysteine and the risk of age-related macular degeneration: a systematic review and meta-analysis, Sci. Rep. 5 (2015) 10585, https://doi.org/10.1038/srep10585.

[32] N. Hu, X. Wang, The level of homocysteine in amyotrophic lateral sclerosis: a systematic review and meta-analysis, Neurol. Sci. 44 (4) (2023) 1185-1192, https://doi.org/10.1007/s10072-022-06518-6.

[33] J. Stein, J. Geisel, R. Obeid, Association between neuropathy and b-vitamins: a systematic review and meta-analysis, Eur. J. Neurol. 28 (6) (2021) 2054-2064, https://doi.org/10.1111/ene.14786.

[34] Y. Chen, H. Zou, M. Peng, Y. Chen, Association between homocysteine levels in acute stroke and poststroke depression: a systematic review and meta-analysis, Brain Behav. 12 (6) (2022) e2626, https://doi.org/10.1002/brb3.2626.

[35] V. Jaiswal, S.P. Ang, V. Suresh, A. Joshi, A. Halder, K. Rajak, et al., Association between baseline high-sensitive c-reactive protein, homocysteine levels, and post-stroke depression among stroke patients: a systematic review, meta-analysis, and meta-regression, Curr. Probl. Cardiol. 49 (3) (2024) 102338, https://doi.org/10.1016/j.cpcardiol.2023.102338.

[36] F. Moradi, K. Lotfi, M. Armin, C. Clark, G. Askari, M.H. Rouhani, The association between serum homocysteine and depression: a systematic review and meta-analysis of observational studies, Eur. J. Clin. Invest. 51 (5) (2021) e13486, https://doi.org/10.1111/eci.13486.

[37] X. Li, J. Yuan, J. Han, W. Hu, Serum levels of homocysteine, vitamin b12 and folate in patients with multiple sclerosis: an updated meta-analysis, Int. J. Med. Sci. 17 (6) (2020) 751-761, https://doi.org/10.7150/ijms.42058.

[38] Y.J. Zhang, L. Zhang, X.L. Huang, Y. Duan, L.J. Yang, J. Wang, The association between homocysteine and systemic sclerosis: a review of the literature and meta-analysis, Mod. Rheumatol. 28 (4) (2018) 681-689, https://doi.org/10.1080/14397595.2017.1386844.

[39] S. Cong, C. Xiang, H. Wang, S. Cong, Diagnostic utility of fluid biomarkers in multiple system atrophy: a systematic review and meta-analysis, J. Neurol. 268 (8) (2021) 2703-2712, https://doi.org/10.1007/s00415-020-09781-9.

[40] A. Nishi, S. Numata, A. Tajima, M. Kinoshita, K. Kikuchi, S. Shimodera, et al., Meta-analyses of blood homocysteine levels for gender and genetic association studies of the mthfr c677t polymorphism in schizophrenia, Schizophr. Bull. 40 (5) (2014) 1154-1163, https://doi.org/10.1093/schbul/sbt154.

[41] B.Q. Guo, H.B. Li, S.B. Ding, Blood homocysteine levels in children with autism spectrum disorder: an updated systematic review and meta-analysis, Psychiatry Res. 291 (2020) 113283, https://doi.org/10.1016/j.psychres.2020.113283.

[42] D. Fraguas, C.M. Díaz-Caneja, M. Ayora, F. Hernández-álvarez, A. Rodríguez-Quiroga, S. Recio, et al., Oxidative stress and inflammation in first-episode psychosis: a systematic review and meta-analysis, Schizophr. Bull. 45 (4) (2019) 742-751, https://doi.org/10.1093/schbul/sby125.

[43] S. Yan, H. Liu, Y. Yu, N. Han, Du W, Changes of serum homocysteine and vitamin b12, but not folate are correlated with obsessive-compulsive disorder: a systematic review and meta-analysis of case-control studies, Front. Psychiatry 13 (2022) 754165, https://doi.org/10.3389/fpsyt.2022.754165.

[44] I. Liampas, V. Siokas, A.A. Mentis, A.M. Aloizou, M. Dastamani, Z. Tsouris, et al., Serum homocysteine, pyridoxine, folate, and vitamin b12 levels in migraine: systematic review and meta-analysis, Headache 60 (8) (2020) 1508-1534, https://doi.org/10.1111/head.13892.

[45] K. Li, J. Zhang, Y. Qin, Y.X. Wei, Association between serum homocysteine level and obstructive sleep apnea: a meta-analysis, Biomed Res. Int. 2017 (2017) 7234528, https://doi.org/10.1155/2017/7234528.

[46] L. Fu, Y.N. Li, D. Luo, S. Deng, Y.Q. Hu, Plausible relationship between homocysteine and obesity risk via mthfr gene: a meta-analysis of 38,317 individuals implementing mendelian randomization, Diabetes Metab. Syndr. Obes. 12 (2019) 1201-1212, https://doi.org/10.2147/DMSO.S205379.

[47] J. Wang, D. You, H. Wang, Y. Yang, D. Zhang, J. Lv, et al., Association between homocysteine and obesity: a meta-analysis, J Evid.-Based Med. 14 (3) (2021) 208-217, https://doi.org/10.1111/jebm.12412.

[48] J.X. Wang, D.Y. You, H.P. Wang, C.G. Zou, Y.H. Yang, D. Zhang, et al., Association between homocysteine and type 2 diabetes mellitus: a systematic review and meta-analysis, Int. Diabetes Dev. Ctries. 41 (4) (2021) 553-562, https://doi.org/10.1007/s13410-021-00933-9.

[49] L.Q. Zheng, H.L. Zhang, Z.H. Guan, M.Y. Hu, T. Zhang, S.J. Ge, Elevated serum homocysteine level in the development of diabetic peripheral neuropathy, Genet. Mol. Res. 14 (4) (2015) 15365-15375, https://doi.org/10.4238/2015.November.30.14.

[50] H. Guo, X. Chen, H. Zhang, X. Zhang, Serum homocysteine levels and diabetic neuropathy in patients with type 2 diabetes mellitus: a systematic review and meta-analysis, Int. J. Clin. Exp. Med. 9 (10) (2016) 19588-19594,

[51] Y. Feng, M.Q. Shan, L. Bo, X.Y. Zhang, J. Hu, Association of homocysteine with type 1 diabetes mellitus: a meta-analysis, Int. J. Clin. Exp. Med. 8 (8) (2015) 12529-12538,

[52] Y. Zheng, H.Y. Deng, Z.Y. Qiao, F.X. Gong, Homocysteine level and gestational diabetes mellitus: a systematic review and meta-analysis, Gynecol. Endocrinol. 37 (11) (2021) 987-994, https://doi.org/10.1080/09513590.2021.1967314.

[53] X. Lei, G. Zeng, Y. Zhang, Q. Li, J. Zhang, Z. Bai, et al., Association between homocysteine level and the risk of diabetic retinopathy: a systematic review and meta-analysis, Diabetol. Metab. Syndr. 10 (2018) 61, https://doi.org/10.1186/s13098-018-0362-1.

[54] J.R. Ulloque-Badaracco, E.A. Hernandez-Bustamante, E.A. Alarcon-Braga, A. Al-Kassab-Córdova, J.C. Cabrera-Guzmán, P. Herrera-Añazco, et al., Vitamin b12, folate, and homocysteine in metabolic syndrome: a systematic review and meta-analysis, Front. Endocrinol. 14 (2023) 1221259, https://doi.org/10.3389/fendo.2023.1221259.

[55] M. Merashli, T. Bucci, D. Pastori, P. Pignatelli, A. Arcaro, F. Gentile, et al., Plasma homocysteine in behcet's disease: a systematic review and meta-analysis, Thromb. Haemost. 122 (7) (2022) 1209-1220, https://doi.org/10.1055/s-0041-1740637.

[56] M. La Regina, F. Orlandini, D. Prisco, F. Dentali, Homocysteine in vascular behcet disease: a meta-analysis, Arterioscler. Thromb. Vasc. Biol. 30 (10) (2010) 2067-2074, https://doi.org/10.1161/ATVBAHA.110.207068.

[57] M. Merashli, T. Bucci, D. Pastori, P. Pignatelli, A. Arcaro, F. Gentile, et al., A meta-analysis of plasma homocysteine in buerger's disease, Thromb. Haemost. 122 (7) (2022) 1243-1246, https://doi.org/10.1055/s-0041-1742165.

[58] S.F. Zhang, L.Z. Li, W. Zhang, J.R. Guo, F.F. Liu, K. Ma, et al., Association between plasma homocysteine levels and subclinical hypothyroidism in adult subjects: a meta-analysis, Horm. Metab. Res. 52 (9) (2020) 625-638, https://doi.org/10.1055/a-1199-2633.

[59] R. Fan, A. Zhang, F. Zhong, Association between homocysteine levels and all-cause mortality: a dose-response meta-analysis of prospective studies, Sci. Rep. 7 (1) (2017) 4769, https://doi.org/10.1038/s41598-017-05205-3.

[60] H.Y. Peng, C.F. Man, J. Xu, Y. Fan, Elevated homocysteine levels and risk of cardiovascular and all-cause mortality: a meta-analysis of prospective studies, J. Zhejiang Univ. Sci. B 16 (1) (2015) 78-86, https://doi.org/10.1631/jzus.B1400183.

[61] D. Zhang, X. Wen, W. Wu, Y. Guo, W. Cui, Elevated homocysteine level and folate deficiency associated with increased overall risk of carcinogenesis: meta-analysis of 83 case-control studies involving 35,758 individuals, PLoS One 10 (5) (2015) e123423, https://doi.org/10.1371/journal.pone.0123423.

[62] J. Yang, H. Li, H. Deng, Z. Wang, Association of one-carbon metabolism-related vitamins (folate, b6, b12), homocysteine and methionine with the risk of lung cancer: systematic review and meta-analysis, Front. Oncol. 8 (2018) 493, https://doi.org/10.3389/fonc.2018.00493.

[63] J. Xu, X. Zhao, S. Sun, P. Ni, C. Li, A. Ren, et al., Homocysteine and digestive tract cancer risk: a dose-response meta-analysis, J. Oncol. 2018 (2018) 3720684, https://doi.org/10.1155/2018/3720684.

[64] J. Heinz, S. Kropf, C. Luley, J. Dierkes, Homocysteine as a risk factor for cardiovascular disease in patients treated by dialysis: a meta-analysis, Am. J. Kidney. Dis. 54 (3) (2009) 478-489, https://doi.org/10.1053/j.ajkd.2009.01.266.

[65] Y. Zhong, F. Yan, W. Jie, Y. Zhou, F. Fang, Correlation between serum homocysteine level and ulcerative colitis: a meta-analysis, Pteridines 30 (1) (2019) 114-120, https://doi.org/10.1515/pteridines-2019-0013.

[66] A. Oussalah, J.L. Guéant, L. Peyrin-Biroulet, Meta-analysis: hyperhomocysteinaemia in inflammatory bowel diseases, Aliment. Pharmacol. Ther. 34 (10) (2011) 1173-1184, https://doi.org/10.1111/j.1365-2036.2011.04864.x.

[67] S. Yuan, J. Chen, L. Dan, Y. Xie, Y. Sun, X. Li, et al., Homocysteine, folate, and nonalcoholic fatty liver disease: a systematic review with meta-analysis and mendelian randomization investigation, Am. J. Clin. Nutr. 116 (6) (2022) 1595-1609, https://doi.org/10.1093/ajcn/nqac285.

[68] X. Qi, Z. Yang, V. De Stefano, D. Fan, Methylenetetrahydrofolate reductase c677t gene mutation and hyperhomocysteinemia in budd-chiari syndrome and portal vein thrombosis: a systematic review and meta-analysis of observational studies, Hepatol. Res. 44 (14) (2014) E480-E498, https://doi.org/10.1111/hepr.12348.

[69] F. Xu, X. Zhao, S.M. Zeng, L. Li, H.B. Zhong, M. Li, Homocysteine, b vitamins, methylenetetrahydrofolate reductase gene, and risk of primary open-angle glaucoma: a meta-analysis, Ophthalmology 119 (12) (2012) 2493-2499, https://doi.org/10.1016/j.ophtha.2012.06.025.

[70] F. Xu, L. Zhang, M. Li, Plasma homocysteine, serum folic acid, serum vitamin b12, serum vitamin b6, mthfr and risk of pseudoexfoliation glaucoma: a meta-analysis, Graefes. Arch. Clin. Exp. Ophthalmol. 250 (7) (2012) 1067-1074, https://doi.org/10.1007/s00417-011-1877-4.

[71] J. Li, F. Xu, R. Zeng, H. Gong, Y. Lan, Plasma homocysteine, serum folic acid, serum vitamin b12, serum vitamin b6, mthfr, and risk of normal-tension glaucoma, J. Glaucoma 25 (2) (2016) e94-e98, https://doi.org/10.1097/IJG.0000000000000269.

[72] X. Niu, Y. Chen, Y. Zhong, X. Xiao, The relationship between serum homocysteine levels and sudden sensorineural hearing loss: a meta-analysis, Eur. Arch. Otorhinolaryngol. 280 (5) (2023) 2091-2097, https://doi.org/10.1007/s00405-023-07829-w.

[73] D. Li, M. Zhou, X. Peng, H. Sun, Homocysteine, methylenetetrahydrofolate reductase c677t polymorphism, and risk of retinal vein occlusion: an updated meta-analysis, BMC Ophthalmol. 14 (2014) 147, https://doi.org/10.1186/1471-2415-14-147.

[74] X. Huang, Y. Yang, Y. Duan, Y.Q. Kuang, D. Lin, Homocysteine in retinal artery occlusive disease: a meta-analysis of cohort studies, Sci. Rep. 7 (1) (2017) 15708, https://doi.org/10.1038/s41598-017-16065-2.

[75] M. Murri, M. Luque-Ramírez, M. Insenser, M. Ojeda-Ojeda, H.F. Escobar-Morreale, Circulating markers of oxidative stress and polycystic ovary syndrome (pcos): a systematic review and meta-analysis, Hum. Reprod. Update 19 (3) (2013) 268-288, https://doi.org/10.1093/humupd/dms059.

[76] Y. Meng, X. Chen, Z. Peng, X. Liu, Y. Sun, S. Dai, Association between high serum homocysteine levels and biochemical characteristics in women with polycystic ovarian syndrome: a systematic review and meta-analysis, PLoS One 11 (6) (2016) e157389, https://doi.org/10.1371/journal.pone.0157389.

[77] C. Zhang, J. Hu, X. Wang, H. Gu, High level of homocysteine is associated with pre-eclampsia risk in pregnant woman: a meta-analysis, Gynecol. Endocrinol. 38 (9) (2022) 705-712, https://doi.org/10.1080/09513590.2022.2110233.

[78] R. Bala, R. Verma, P. Verma, V. Singh, N. Yadav, S. Rajender, et al., Hyperhomocysteinemia and low vitamin b12 are associated with the risk of early pregnancy loss: a clinical study and meta-analyses, Nutr. Res. 91 (2021) 57-66, https://doi.org/10.1016/j.nutres.2021.05.002.

[79] J. Diao, L. Luo, J. Li, S. Zhang, Y. Li, J. Qin, Maternal homocysteine and folate levels and risk of recurrent spontaneous abortion: a meta‐analysis of observational studies, J. Obstet. Gynaecol. Res. 46 (12) (2020) 2461-2473, https://doi.org/10.1111/jog.14500.

[80] U. Yadav, P. Kumar, V. Rai, Maternal biomarkers for early prediction of the neural tube defects pregnancies, Birth Defects Res. 113 (7) (2021) 589-600, https://doi.org/10.1002/bdr2.1842.

[81] A. Sansone, A. Cignarelli, M. Sansone, F. Romanelli, G. Corona, D. Gianfrilli, et al., Serum homocysteine levels in men with and without erectile dysfunction: a systematic review and meta-analysis, Int. J. Endocrinol. 2018 (2018) 7424792, https://doi.org/10.1155/2018/7424792.

[82] R. Blanco, A. Colombo, R. Pardo, J. Suazo, Maternal biomarkers of methylation status and non-syndromic orofacial cleft risk: a meta-analysis, Int. J. Oral. Maxillofac. Surg. 45 (11) (2016) 1323-1332, https://doi.org/10.1016/j.ijom.2016.06.011.

[83] A. Verkleij-Hagoort, J. Bliek, F. Sayed-Tabatabaei, N. Ursem, E. Steegers, R. Steegers-Theunissen, Hyperhomocysteinemia and mthfr polymorphisms in association with orofacial clefts and congenital heart defects: a meta-analysis, Am. J. Med. Genet. A 143A (9) (2007) 952-960, https://doi.org/10.1002/ajmg.a.31684.

[84] J. Yang, X. Hu, Q. Zhang, H. Cao, J. Wang, B. Liu, Homocysteine level and risk of fracture: a meta-analysis and systematic review, Bone 51 (3) (2012) 376-382, https://doi.org/10.1016/j.bone.2012.05.024.

[85] A.A. Mangoni, A. Zinellu, Transsulfuration and folate pathways in rheumatoid arthritis: a systematic review and meta-analysis, Eur. J. Clin. Invest. 54 (4) (2024) e14158, https://doi.org/10.1111/eci.14158.

[86] F. Zhao, L. Guo, X. Wang, Y. Zhang, Correlation of oxidative stress-related biomarkers with postmenopausal osteoporosis: a systematic review and meta-analysis, Arch. Osteoporos. 16 (1) (2021) 4, https://doi.org/10.1007/s11657-020-00854-w.

[87] P. Ames, A. Arcaro, M. Caruso, M. Graf, V. Marottoli, F. Gentile, Relevance of plasma homocysteine and methylenetetrahydrofolate reductase 677tt genotype in sickle cell disease: a systematic review and meta-analysis, Int. J. Mol. Sci. 23 (23) (2022) https://doi.org/10.3390/ijms232314641.

[88] T.Y. Tsai, C.Y. Kuo, Y.C. Huang, Serum homocysteine, folate, and vitamin b(12) levels in patients with vitiligo and their potential roles as disease activity biomarkers: a systematic review and meta-analysis, J. Am. Acad. Dermatol. 80 (3) (2019) 646-654, https://doi.org/10.1016/j.jaad.2018.08.029.

[89] T.Y. Tsai, H. Yen, Y.C. Huang, Serum homocysteine, folate and vitamin b(12) levels in patients with psoriasis: a systematic review and meta-analysis, Br. J. Dermatol. 180 (2) (2019) 382-389, https://doi.org/10.1111/bjd.17034.

[90] T.Y. Tsai, T.H. Lee, H.H. Wang, T.H. Yang, I.J. Chang, Y.C. Huang, Serum homocysteine, folate, and vitamin b(12) levels in patients with systemic lupus erythematosus: a meta-analysis and meta-regression, J. Am. Coll. Nutr. 40 (5) (2021) 443-453, https://doi.org/10.1080/07315724.2020.1788472.

[91] H.H. Li, X.Q. Li, L.T. Sai, Y. Cui, J.H. Xu, C. Zhou, et al., Association of homocysteine with ankylosing spondylitis: a systematic review and meta-analysis, Adv. Rheumatol. 61 (1) (2021) 17, https://doi.org/10.1186/s42358-021-00175-7.

[92] R. Deminice, T.C. Silva, V.H. de Oliveira, Elevated homocysteine levels in human immunodeficiency virus-infected patients under antiretroviral therapy: a meta-analysis, World J Virol 4 (2) (2015) 147-155, https://doi.org/10.5501/wjv.v4.i2.147.

[93] A. Zinellu, A.A. Mangoni, Arginine, transsulfuration, and folic acid pathway metabolomics in chronic obstructive pulmonary disease: a systematic review and meta-analysis, Cells 12 (17) (2023) https://doi.org/10.3390/cells12172180.

[94] W. Chen, J. Feng, P. Ji, Y. Liu, H. Wan, J. Zhang, Association of hyperhomocysteinemia and chronic kidney disease in the general population: a systematic review and meta-analysis, BMC Nephrol. 24 (1) (2023) 247, https://doi.org/10.1186/s12882-023-03295-y.

[95] S.C. Larsson, M. Traylor, H.S. Markus, Homocysteine and small vessel stroke: a mendelian randomization analysis, Ann. Neurol. 85 (4) (2019) 495-501, https://doi.org/10.1002/ana.25440.

[96] S. Yuan, A.M. Mason, P. Carter, S. Burgess, S.C. Larsson, Homocysteine, b vitamins, and cardiovascular disease: a mendelian randomization study, BMC Med. 19 (1) (2021) 97, https://doi.org/10.1186/s12916-021-01977-8.

[97] L. Miao, G.X. Deng, R.X. Yin, R.J. Nie, S. Yang, Y. Wang, et al., No causal effects of plasma homocysteine levels on the risk of coronary heart disease or acute myocardial infarction: a mendelian randomization study, Eur. J. Prev. Cardiol. 28 (2) (2021) 227-234, https://doi.org/10.1177/2047487319894679.

[98] J.B. van Meurs, G. Pare, S.M. Schwartz, A. Hazra, T. Tanaka, S.H. Vermeulen, et al., Common genetic loci influencing plasma homocysteine concentrations and their effect on risk of coronary artery disease, Am. J. Clin. Nutr. 98 (3) (2013) 668-676, https://doi.org/10.3945/ajcn.112.044545.

[99] T. Xu, S. Chen, F. Yang, Y. Wang, K. Zhang, G. Fu, et al., The impact of homocysteine on the risk of coronary artery diseases in individuals with diabetes: a mendelian randomization study, Acta Diabetol. 58 (3) (2021) 301-307, https://doi.org/10.1007/s00592-020-01608-3.

[100] X. Sun, Y. Lu, Z. Wang, Q. Wang, L. Zheng, No causal association between plasma homocysteine levels and atrial fibrillation: a mendelian randomization study, Nutr. Metab. Carbiovasc. Dis. 31 (2) (2021) 587-591, https://doi.org/10.1016/j.numecd.2020.10.012.

[101] S. Chen, F. Yang, T. Xu, Y. Wang, K. Zhang, G. Fu, et al., Appraising the causal association of plasma homocysteine levels with atrial fibrillation risk: a two-sample mendelian randomization study, Front. Genet. 12 (2021) 619536, https://doi.org/10.3389/fgene.2021.619536.

[102] X. Wang, Z. Chen, W. Tian, J. Zhang, Q. Li, J. Ju, et al., Plasma homocysteine levels and risk of congestive heart failure or cardiomyopathy: a mendelian randomization study, Front. Cardiovasc. Med. 10 (2023) 1030257, https://doi.org/10.3389/fcvm.2023.1030257.

[103] W. Liu, L. Zhang, S. Li, C. Liu, Y. Tong, H. Fang, et al., A mendelian randomization study of plasma homocysteine levels and cerebrovascular and neurodegenerative diseases, Front. Genet. 12 (2021) 653032, https://doi.org/10.3389/fgene.2021.653032.

[104] C. Ma, W. Zhang, L. Mao, G. Zhang, Y. Shen, H. Chang, et al., Hyperhomocysteinemia and intracranial aneurysm: a mendelian randomization study, Front. Neurol. 13 (2022) 948989, https://doi.org/10.3389/fneur.2022.948989.

[105] Y. Cao, N. Su, D. Zhang, L. Zhou, M. Yao, S. Zhang, et al., Correlation between total homocysteine and cerebral small vessel disease: a mendelian randomization study, Eur. J. Neurol. 28 (6) (2021) 1931-1938, https://doi.org/10.1111/ene.14708.

[106] Z. Wen, X. Feng, X. Tong, C. Peng, A. Xu, H. Fan, et al., A mendelian randomisation, propensity score matching study to investigate causal association between serum homocysteine and intracranial aneurysm, Stroke Vasc. Neurol. 2023) https://doi.org/10.1136/svn-2023-002414.

[107] L. Fu, Y.N. Li, D. Luo, S. Deng, B. Wu, Y.Q. Hu, Evidence on the causal link between homocysteine and hypertension from a meta-analysis of 40 173 individuals implementing mendelian randomization, J. Clin. Hypertens. 21 (12) (2019) 1879-1894, https://doi.org/10.1111/jch.13737.

[108] Y. Li, K. Wang, T. Duan, Plasma total homocysteine and risk of hypertension in pregnancy: a mendelian randomization study, Int. J. Clin. Exp. Med. 12 (6) (2019) 6561-6569,

[109] S.C. Larsson, M. Traylor, R. Malik, M. Dichgans, S. Burgess, H.S. Markus, Modifiable pathways in alzheimer's disease: mendelian randomisation analysis, BMJ. 359 (2017) j5375, https://doi.org/10.1136/bmj.j5375.

[110] Q. Hu, W. Teng, J. Li, F. Hao, N. Wang, Homocysteine and alzheimer's disease: evidence for a causal link from mendelian randomization, J. Alzheimers Dis. 52 (2) (2016) 747-756, https://doi.org/10.3233/JAD-150977.

[111] S.P. Wu, J.J. Ma, Y.W. Qi, J.W. Zhang, Plasma homocysteine levels and risk of vascular dementia: a mendelian randomization study, Int. J. Clin. Exp. Med. 10 (6) (2017) 9142-9151,

[112] Y. Zhao, D. Tian, N. Guo, C. Zhang, R. Zhu, X. Liu, et al., Investigating the causality of metabolites involved in one-carbon metabolism with the risk and age at onset of parkinson's disease: a two-sample mendelian randomization study, Neurobiol. Aging 108 (2021) 196-199, https://doi.org/10.1016/j.neurobiolaging.2021.06.023.

[113] H. Peng, X. Wu, J. Lin, W. Guan, Genetically predicted circulating homocysteine, vitamin b12, and folate levels and risk of multiple sclerosis: evidence from a two-sample mendelian randomization analysis, Mult. Scler. Relat. Disord. 56 (2021) 103255, https://doi.org/10.1016/j.msard.2021.103255.

[114] S. Numata, M. Kinoshita, A. Tajima, A. Nishi, I. Imoto, T. Ohmori, Evaluation of an association between plasma total homocysteine and schizophrenia by a mendelian randomization analysis, BMC Med. Genet. 16 (2015) 54, https://doi.org/10.1186/s12881-015-0197-7.

[115] J. Yu, R. Xue, Q. Wang, H. Yu, X. Liu, The effects of plasma homocysteine level on the risk of three major psychiatric disorders: a mendelian randomization study, Front. Psychiatry 13 (2022) 841429, https://doi.org/10.3389/fpsyt.2022.841429.

[116] T. Jin, W. Huang, Q. Pang, Z. He, L. Yuan, H. Zhang, et al., Inferring the genetic effects of serum homocysteine and vitamin b levels on autism spectral disorder through mendelian randomization, Eur. J. Nutr. 63 (3) (2024) 977-986, https://doi.org/10.1007/s00394-024-03329-7.

[117] W. Gao, W.W. Zhu, Y.H. Yu, J. Wang, Plasma homocysteine level, estradiol level, and brain atrophy: a mendelian randomization study, Cereb. Cortex. 34 (3) (2024) https://doi.org/10.1093/cercor/bhae112.

[118] J. Kumar, E. Ingelsson, L. Lind, T. Fall, No evidence of a causal relationship between plasma homocysteine and type 2 diabetes: a mendelian randomization study, Front. Cardiovasc. Med. 2 (2015) 11, https://doi.org/10.3389/fcvm.2015.00011.

[119] Y. Cheng, C. Wang, X. Zhang, Y. Zhao, B. Jin, C. Wang, et al., Circulating homocysteine and folate concentrations and risk of type 2 diabetes: a retrospective observational study in chinese adults and a mendelian randomization analysis, Front. Cardiovasc. Med. 9 (2022) 978998, https://doi.org/10.3389/fcvm.2022.978998.

[120] L. Ma, Q. Liu, Y. Jiang, H. Zhao, T. Zhao, Y. Cao, et al., Genetically elevated circulating homocysteine concentrations increase the risk of diabetic kidney disease in chinese diabetic patients, J. Cell. Mol. Med. 23 (4) (2019) 2794-2800, https://doi.org/10.1111/jcmm.14187.

[121] H.S. Lee, S. In, T. Park, The homocysteine and metabolic syndrome: a mendelian randomization study, Nutrients 13 (7) (2021) https://doi.org/10.3390/nu13072440.

[122] L. Fu, Y. Wang, Y.Q. Hu, Association between homocysteine and nonalcoholic fatty liver disease: mendelian randomisation study, Eur. J. Clin. Invest. 53 (3) (2023) e13895, https://doi.org/10.1111/eci.13895.

[123] P. Chen, Z. Yang, L. Guo, Y. Huang, J. Li, X. Chen, Effects of homocysteine on nonalcoholic fatty liver related disease: a mendelian randomization study, Front. Mol. Biosci. 9 (2022) 1083855, https://doi.org/10.3389/fmolb.2022.1083855.

[124] T. Wang, C. Ren, J. Ni, H. Ding, Q. Qi, C. Yan, et al., Genetic association of plasma homocysteine levels with gastric cancer risk: a two-sample mendelian randomization study, Cancer. Epidemiol. Biomarkers. Prev. 29 (2) (2020) 487-492, https://doi.org/10.1158/1055-9965.EPI-19-0724.

[125] W. Xu, Y. Cheng, H. Zhu, Evaluation of an association of blood homocysteine levels with gastric cancer risk from 27 case-control studies, Medicine. (Baltimore). 95 (20) (2016) e3700, https://doi.org/10.1097/MD.0000000000003700.

[126] Q. He, Z. Yang, Y. Sun, Z. Qu, X. Jia, J. Li, et al., The impact of homocysteine on the risk of hormone-related cancers: a mendelian randomization study, Front. Nutr. 8 (2021) 645371, https://doi.org/10.3389/fnut.2021.645371.

[127] Y. Xuan, X.H. Li, Z.Q. Hu, Z.M. Teng, D.J. Hu, A mendelian randomization study of plasma homocysteine and multiple myeloma, Sci. Rep. 6 (2016) 25204, https://doi.org/10.1038/srep25204.

[128] C.K. Choi, S.S. Kweon, Y.H. Lee, H.S. Nam, S.W. Choi, H.Y. Kim, et al., Association between plasma homocysteine level and mortality: a mendelian randomization study, Korean Circ. J. 53 (10) (2023) 710-719, https://doi.org/10.4070/kcj.2023.0089.

[129] P. Wang, L. Liu, S.F. Lei, Causal effects of homocysteine levels on the changes of bone mineral density and risk for bone fracture: a two-sample mendelian randomization study, Clin. Nutr. 40 (4) (2021) 1588-1595, https://doi.org/10.1016/j.clnu.2021.02.045.

[130] H. Hong, L. Chen, Y. Zhong, Z. Yang, W. Li, C. Song, et al., Associations of homocysteine, folate, and vitamin b12 with osteoarthritis: a mendelian randomization study, Nutrients 15 (7) (2023) https://doi.org/10.3390/nu15071636.

[131] L. Fu, Y. Wang, Y.Q. Hu, Causal effects of b vitamins and homocysteine on obesity and musculoskeletal diseases: a mendelian randomization study, Front. Nutr. 9 (2022) 1048122, https://doi.org/10.3389/fnut.2022.1048122.

[132] C. Wang, X. Zhang, B. Qiu, Genetically predicted circulating serum homocysteine levels on osteoporosis: a two-sample mendelian randomization study, Sci. Rep. 13 (1) (2023) 9063, https://doi.org/10.1038/s41598-023-35472-2.

[133] Y. Hu, P. Tan, J. Wang, J. Zeng, Q. Li, S. Yan, et al., Mendelian randomization study to investigate the causal relationship between plasma homocysteine and chronic obstructive pulmonary disease, World J. Emerg. Med. 14 (5) (2023) 367-371, https://doi.org/10.5847/wjem.j.1920-8642.2023.078.

[134] L. Wang, X. Li, A. Montazeri, A.J. Macfarlane, F. Momoli, S. Duthie, et al., Phenome-wide association study of genetically predicted b vitamins and homocysteine biomarkers with multiple health and disease outcomes: analysis of the uk biobank, Am. J. Clin. Nutr. 117 (3) (2023) 564-575, https://doi.org/10.1016/j.ajcnut.2023.01.005.

[135] Y. Xiong, Y. Zhang, F. Zhang, C. Wu, P. Luo, F. Qin, et al., Genetic evidence supporting the causal role of homocysteine in chronic kidney disease: a mendelian randomization study, Front. Nutr. 9 (2022) 843534, https://doi.org/10.3389/fnut.2022.843534.

[136] A.D. Kjaergaard, Y. Wu, W.K. Ming, Z. Wang, M.N. Kjaergaard, C. Ellervik, Homocysteine and female fertility, pregnancy loss and offspring birthweight: a two-sample mendelian randomization study, Eur. J. Clin. Nutr. 76 (1) (2022) 40-47, https://doi.org/10.1038/s41430-021-00898-2.

[137] C. Chen, S. Liu, J. Liu, Z. Zheng, Y. Zheng, Z. Lin, et al., No causal effect of genetically determined circulating homocysteine levels on psoriasis in the european population: evidence from a mendelian randomization study, Front. Immunol. 14 (2023) 1288632, https://doi.org/10.3389/fimmu.2023.1288632.

[138] R. Clarke, J. Halsey, D. Bennett, S. Lewington, Homocysteine and vascular disease: review of published results of the homocysteine-lowering trials, J. Inherit. Metab. Dis. 34 (1) (2011) 83-91, https://doi.org/10.1007/s10545-010-9235-y.

[139] G. Dai, Du H, H. Wang, Y. Wei, Y. Xie, Z. Li, [Preventive effect of vitamin b supplementation on recurrent stroke: a meta-analysis], Zhonghua Wei Zhong Bing Ji Jiu Yi Xue 29 (5) (2017) 419-424, https://doi.org/10.3760/cma.j.issn.2095-4352.2017.05.007.

[140] H. Fu, J. He, C. Li, Z. Deng, H. Chang, Folate intake and risk of colorectal cancer: a systematic review and up-to-date meta-analysis of prospective studies, Eur. J. Cancer. Prev. 32 (2) (2023) 103-112, https://doi.org/10.1097/CEJ.0000000000000744.

[141] L.M. Garcia, Baron JA, T.K. Omsland, A.J. Søgaard, H.E. Meyer, Homocysteine-lowering treatment and the risk of fracture: secondary analysis of a randomized controlled trial and an updated meta-analysis, JBMR Plus 2 (5) (2018) 295-303, https://doi.org/10.1002/jbm4.10045.

[142] M.J. Jardine, A. Kang, S. Zoungas, S.D. Navaneethan, T. Ninomiya, S.U. Nigwekar, et al., The effect of folic acid based homocysteine lowering on cardiovascular events in people with kidney disease: systematic review and meta-analysis, BMJ. 344 (2012) e3533, https://doi.org/10.1136/bmj.e3533.

[143] Y. Li, T. Huang, Y. Zheng, T. Muka, J. Troup, F.B. Hu, Folic acid supplementation and the risk of cardiovascular diseases: a meta-analysis of randomized controlled trials, J. Am. Heart Assoc. 5 (8) (2016) https://doi.org/10.1161/JAHA.116.003768.

[144] E.R. Miller, S. Juraschek, R. Pastor-Barriuso, L.A. Bazzano, L.J. Appel, E. Guallar, Meta-analysis of folic acid supplementation trials on risk of cardiovascular disease and risk interaction with baseline homocysteine levels, Am. J. Cardiol. 106 (4) (2010) 517-527, https://doi.org/10.1016/j.amjcard.2010.03.064.

[145] S.U. Nigwekar, A. Kang, S. Zoungas, A. Cass, M.P. Gallagher, S. Kulshrestha, et al., Interventions for lowering plasma homocysteine levels in dialysis patients, Cochrane Database Syst Rev. 2016 (5) (2016) CD4683, https://doi.org/10.1002/14651858.CD004683.pub4.

[146] J. Park, G. Saposnik, B. Ovbiagele, D. Markovic, A. Towfighi, Effect of b-vitamins on stroke risk among individuals with vascular disease who are not on antiplatelets: a meta-analysis, Int. J. Stroke 11 (2) (2016) 206-211, https://doi.org/10.1177/1747493015616512.

[147] X. Qin, Y. Huo, C.B. Langman, F. Hou, Y. Chen, D. Matossian, et al., Folic acid therapy and cardiovascular disease in esrd or advanced chronic kidney disease: a meta-analysis, Clin. J. Am. Soc. Nephrol. 6 (3) (2011) 482-488, https://doi.org/10.2215/CJN.05310610.

[148] X. Qin, Y. Huo, D. Xie, F. Hou, X. Xu, X. Wang, Homocysteine-lowering therapy with folic acid is effective in cardiovascular disease prevention in patients with kidney disease: a meta-analysis of randomized controlled trials, Clin. Nutr. 32 (5) (2013) 722-727, https://doi.org/10.1016/j.clnu.2012.12.009.

[149] J. Ruan, X. Gong, J. Kong, H. Wang, X. Zheng, T. Chen, Effect of b vitamin (folate, b6, and b12) supplementation on osteoporotic fracture and bone turnover markers: a meta-analysis, Med. Sci. Monitor 21 (2015) 875-881, https://doi.org/10.12659/MSM.893310.

[150] Z. Wang, W. Zhu, Y. Xing, J. Jia, Y. Tang, B vitamins and prevention of cognitive decline and incident dementia: a systematic review and meta-analysis, Nutr. Rev. 80 (4) (2022) 931-949, https://doi.org/10.1093/nutrit/nuab057.

[151] L. Wang, W. Cui, G. Nan, Y. Yu, Meta-analysis reveals protective effects of vitamin b on stroke patients, Transl. Neurosci. 6 (1) (2015) 150-156, https://doi.org/10.1515/tnsci-2015-0014.

[152] C. Zhang, F.L. Chi, T.H. Xie, Y.H. Zhou, Effect of b-vitamin supplementation on stroke: a meta-analysis of randomized controlled trials, PLoS One 8 (11) (2013) e81577, https://doi.org/10.1371/journal.pone.0081577.
